# Improving taxonomic inference from ancient environmental metagenomes by masking microbial-like regions in reference genomes

**DOI:** 10.1093/gigascience/giaf108

**Published:** 2025-10-03

**Authors:** Nikolay Oskolkov, Chenyu Jin, Samantha López Clinton, Benjamin Guinet, Flore Wijnands, Ernst Johnson, Verena E Kutschera, Cormac M Kinsella, Peter D Heintzman, Tom van der Valk

**Affiliations:** Department of Biology, National Bioinformatics Infrastructure Sweden, Science for Life Laboratory, Lund University, SE-223 62 Lund, Sweden; Centre for Palaeogenetics, Svante Arrhenius väg 20C, SE-10691 Stockholm, Sweden; Department of Bioinformatics and Genetics, Swedish Museum of Natural History, SE-104 05 Stockholm, Sweden; Department of Zoology, Stockholm University, SE-106 91 Stockholm, Sweden; Centre for Palaeogenetics, Svante Arrhenius väg 20C, SE-10691 Stockholm, Sweden; Department of Bioinformatics and Genetics, Swedish Museum of Natural History, SE-104 05 Stockholm, Sweden; Department of Zoology, Stockholm University, SE-106 91 Stockholm, Sweden; Centre for Palaeogenetics, Svante Arrhenius väg 20C, SE-10691 Stockholm, Sweden; Department of Bioinformatics and Genetics, Swedish Museum of Natural History, SE-104 05 Stockholm, Sweden; Centre for Palaeogenetics, Svante Arrhenius väg 20C, SE-10691 Stockholm, Sweden; Department of Geological Sciences, Stockholm University, SE-106 91 Stockholm, Sweden; Centre for Palaeogenetics, Svante Arrhenius väg 20C, SE-10691 Stockholm, Sweden; Department of Geological Sciences, Stockholm University, SE-106 91 Stockholm, Sweden; Department of Biochemistry and Biophysics, National Bioinformatics Infrastructure Sweden, Science for Life Laboratory, Stockholm University, Solna, SE-106 91 Stockholm, Sweden; Department of Bioinformatics and Genetics, Swedish Museum of Natural History, SE-104 05 Stockholm, Sweden; Department of Cell and Molecular Biology, National Bioinformatics Infrastructure Sweden, Science for Life Laboratory, Uppsala University, SE-751 24 Uppsala, Sweden; Centre for Palaeogenetics, Svante Arrhenius väg 20C, SE-10691 Stockholm, Sweden; Department of Geological Sciences, Stockholm University, SE-106 91 Stockholm, Sweden; Centre for Palaeogenetics, Svante Arrhenius väg 20C, SE-10691 Stockholm, Sweden; Department of Bioinformatics and Genetics, Swedish Museum of Natural History, SE-104 05 Stockholm, Sweden; Scilifelab, Tomtebodavägen 23, SE-171 65 Solna, Stockholm, Sweden

**Keywords:** environmental DNA, ancient metagenomics, microbial-like regions

## Abstract

Ancient environmental DNA is increasingly vital for reconstructing past ecosystems, particularly when paleontological and archaeological tissue remains are absent. Detecting ancient plant and animal DNA in environmental samples relies on using extensive eukaryotic reference genome databases for profiling metagenomics data. However, many eukaryotic genomes contain regions with high sequence similarity to microbial DNA, which can lead to the misclassification of bacterial and archaeal reads as eukaryotic. This issue is especially problematic in ancient eDNA datasets, where plant and animal DNA is typically present at very low abundance. In this study, we present a method for identifying bacterial- and archaeal-like sequences in eukaryotic genomes and apply it to nearly 3,000 reference genomes from NCBI RefSeq and GenBank (vertebrates, invertebrates, plants) as well as the 1,323 PhyloNorway plant genome assemblies from herbarium material from northern high-latitude regions. We find that microbial-like regions are widespread across eukaryotic genomes and provide a comprehensive resource of their genomic coordinates and taxonomic annotations. This resource enables the masking of microbial-like regions during profiling analyses, thereby improving the reliability of ancient environmental metagenomic datasets for downstream analyses.

## Introduction

Ancient environmental DNA (aeDNA) is a tool for studying past ecosystems, especially in contexts where traditional archaeological and paleontological tissue remains, such as bones and seeds, are absent [1–4]. It consists of genetic traces left by organisms in the environment, such as soil, sediments, or ice, and allows for the reconstruction of past biodiversity and ecological communities to provide insight into species extinction, vegetation changes, and ecosystem responses to climatic shifts and anthropogenic impacts.

The often limited amount of DNA that can be isolated from ancient environmental samples imposes constraints on analytical methods. Coupled with the often low relative abundance of plant and animal DNA preserved in most environments, as compared to microbes, aeDNA analysis primarily relies on a reference-based approach for taxonomic profiling, which assumes similarity between the aeDNA query and the reference genome sequences. Therefore, robust aeDNA-derived community reconstructions are dependent on the accuracy of read identification by comparison to genomic reference databases. Consequently, the quality of both the aeDNA data and the reference databases is crucial for reliable inferences. Microbial-like sequences in reference genomic databases, originating either from nonendogenous sources (contamination) or from evolutionary similarity to microbial genomes (e.g., due to ancient horizontal gene transfer or the endosymbiotic origins of plastids), can be a potential source of false-positive taxonomic identifications. In such cases, microbial sequences present in aeDNA data may be mistakenly classified as belonging to a eukaryotic reference genome due to sequence similarity.

The existence of contaminant-like sequences is a pervasive issue in reference genomes with multiple examples reported in the literature [5–7]. For instance, contaminated reference sequences, such as the presence of a hippopotamus-like sequence in the alpaca mitochondrial reference genome [8] and human sequences in parasitic worm genomes [9], have led to inaccurate inferences of evolutionary relationships [10], divergence times [8], and horizontal gene transfer events [11]. The inclusion of such eukaryotic reference genome contamination, most commonly originating from microbial or human sources, can occur at any stage throughout the genome assembly process [12].

Several analytical approaches have been proposed to address the issue of microbial contamination in reference genomes. For instance, Lu and Salzberg [13] suggested a computational method for masking erroneous sequences from draft genomes of eukaryotic pathogens. This is implemented by splitting the draft pathogenic references into pseudo-reads and filtering them using *k*-mer–based Kraken classification [14, 15] and Bowtie2 alignment [16] against the human genome and National Center for Biotechnological Information Reference Sequence Database (NCBI RefSeq) microbial references. Conterminator is another program for contamination detection in the NCBI GenBank, RefSeq, and nonredundant (NR) reference databases proposed by Steinegger and Salzberg [17]. The program operates by an exhaustive all-against-all sequence comparison across kingdoms by splitting reference sequences into short segments, extracting their *k*-mers, grouping the *k*-mers, and then performing cross-kingdom alignments of the representative sequences in order to predict the contaminating sequences. This approach identified over 2,000,000 contaminated entries in the GenBank database [18]. Furthermore, the Physeter [19] and CheckM [20] tools have also been used to estimate contamination levels in NCBI RefSeq bacterial genomes. Lastly, ongoing efforts by NCBI, such as introducing the FCS-GX tool [21], which uses hashed *k*-mer matches and a curated reference database, in addition to more traditional VecScreen [22] and BLAST [23], are retroactively reducing the prevalence of contaminant-like sequences within the NCBI RefSeq and GenBank databases.

However, these efforts do not address alternative databases, such as those comprising genome-wide data (e.g., PhyloNorway, PhyloAlps [24]) or legacy versions of the NCBI RefSeq database [25, 26], that are commonly used in workflows for large-scale metagenomics analysis (e.g., Kraken [14, 15]). Therefore, there is a need for a generic tool that identifies and removes these problematic sequences, particularly those similar to bacteria and archaea, from any genomic datasets that will be used as reference sequences for ancient environmental metagenomics analysis. In addition, although the microbial NCBI RefSeq is one of the largest available reference databases that has previously been used for estimating the amount of contamination in eukaryotic reference genomes [13, 17], the advent of the more diverse and comprehensive microbial Genome Taxonomy Database (GTDB) [27], allows for greater sensitivity in identifying regions in genome assemblies that are characterized by containing microbial-like sequences.

The aim of this study was therefore threefold. First, we developed a workflow applicable to any eukaryotic reference genome in FASTA format, which outputs exact genomic coordinates of microbial-like sequences in BED format. The coordinate file can then be used to mask the eukaryotic reference genome for various applications, including taxonomic profiling from ancient metagenomics data. Second, we sought higher identification accuracy of microbial-like regions by using the curated and nonredundant microbial GTDB database, the most comprehensive of its type at present. Lastly, to allow for future investigation of the sources and mechanisms of contamination, we annotated and summarized each genomic region identified as microbial-like by the relative contribution of each microbial taxon.

To demonstrate our approach, we aligned all microbial sequences from the GTDB database to 6 panels of eukaryotic reference databases and identified the genomic regions that are similar to bacterial and archaeal sequences. We show that up to 70% of a taxon’s reference genome assembly can have shared similarity with bacteria and archaea (microbial-like). After masking microbial-like regions from the reference genomes, we reanalyzed 2 empirical ancient metagenomic datasets and showed that some eukaryotic species detections can be driven by alignments of reads to the microbial-like regions. We anticipate that masking reference genomes for these microbial-like sequences will greatly reduce reference genome–derived false-positive taxonomic assignments in ancient and modern environmental metagenomic studies.

## Methods

We selected 4,294 reference genomes of varying degrees of completeness and from a broad spectrum of taxonomic groups. This included (i) chromosome-level reference genome assemblies for 96 plants, (ii) 114 invertebrates and (iii) 162 nonmammalian vertebrate species available from NCBI RefSeq, release 213; (iv) 566 chromosome- and scaffold-level mammalian genome assemblies from NCBI GenBank, release 254 (if a species had multiple assemblies, we selected the one with highest N50 value); (v) all 2,033 chromosome- and scaffold-level arthropod reference genomes available in NCBI GenBank, release 256; and (vi) 1,323 genome-skimmed contig-level plant assemblies from the PhyloNorway project (DataverseNO, V1) [28]. We individually constructed Bowtie2 [16] indices for all 4,294 reference genomes in the 6 genome groups.

Next, we fragmented all microbial (bacterial + archaea) reference genomes present in the GTDB dataset ([27]; release 214) into 60-bp-long segments using a sliding window with a 10-bp step. The fragmentation length of 60 bp was chosen to ensure sufficient specificity when matching microbial sequences to eukaryotic references, as it is twice the commonly accepted ∼30-bp minimum threshold for organism-level specificity across the tree of life [14, 15] and matches the average fragment length often obtained from aeDNA datasets. This resulted in a collection of 2.6 * 10^10^ sequences representing microbial sequencing data (reads), which we refer to as “pseudo-reads” in this study. In addition, microbial RefSeq and human hg38 reference genome pseudo-reads were prepared in a similar manner, and we provide these together with the workflow (see also Data Availability). Their use is discussed in Supplementary Material S4 and S5. These reads were aligned to each indexed eukaryotic reference genome using Bowtie2 on the –very-sensitive and –end-to-end settings and allowing up to 10 multimappers to be retained per read. The retention of multimappers ensured that multicopy microbial-like regions from the same microbe within a reference were also detected. In our testing, keeping multimappers greatly improved the detection sensitivity for microbial-like regions in the eukaryotic reference genomes, with this gain saturating after retaining approximately 10 multimapped positions (Supplementary Fig. S1). We considered genomic regions covered by at least 1 microbial pseudo-read as microbial-like. We visually validated a set of the microbial-like regions using the Integrative Genomics Viewer (IGV) [29] and confirmed their coverage by microbial pseudo-reads (Supplementary Fig. S2). For additional details about the alignment procedure, see Supplementary Material S1.

**Figure 1:**
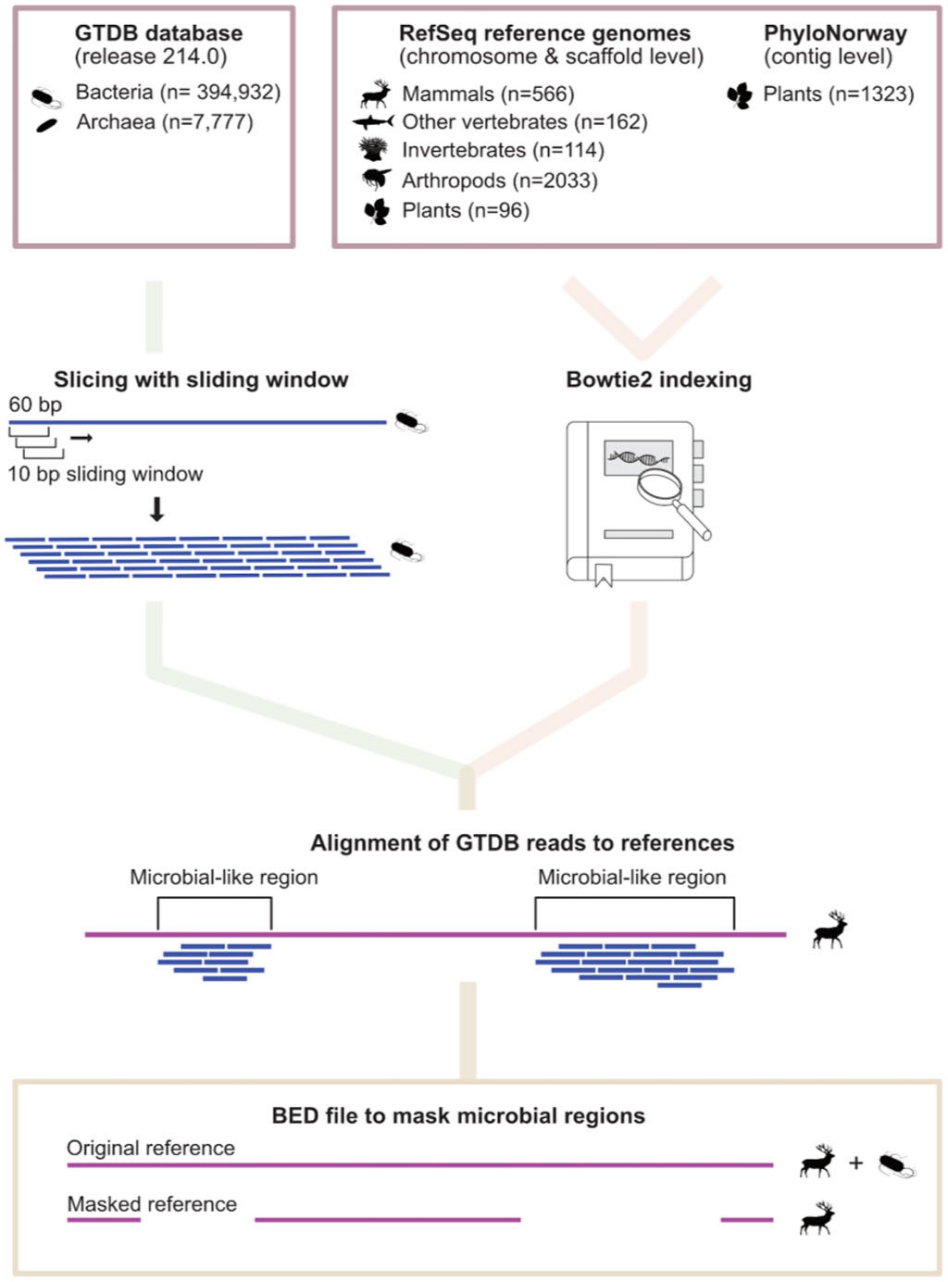
The workflow for detection of microbial-like sequences in eukaryotic reference genomes. Assemblies at different levels from different databases were subjected to the workflow. Microbial reference genomes from GTDB were fragmented into pseudo-reads (60 bp) and aligned to these genomes, retaining up to 10 multimappers to increase detection sensitivity. Microbial-like regions were identified, annotated, and visualized, with microbial abundance summarized in BED files and validated using IGV.

Next, we used *samtools depth* [30] and *bedtools merge* [31] to detect and extract the coordinates of regions in the eukaryotic reference genomes that were covered by microbial pseudo-reads in BED format (Table 1, which also includes data on the abundance of the most prevalent microbes in each identified genomic region). The breadth of coverage of microbial-like sequences was computed as the fraction of reference genome nucleotides covered at least once by microbial pseudo-reads. We validated the successful preparation of GTDB pseudo-reads by aligning them to over 820 randomly selected GTDB reference sequences and observing a median breadth of coverage of 99.1%. This result supports our expectation that the GTDB reference sequences themselves should look to be almost entirely composed of microbial-like sequences. We used *samtools* [30] and custom bash and R scripts for annotating the reference genomes with the most abundant source microbial species. The annotation was done both genome-wide and for each individual microbial-like region in the BED file. In the latter case, only the top 5 most abundant microbial taxa per region were recorded. The entire workflow is schematically presented in Fig. 1 (see also Data Availability).

**Table 1:** Example of BED file with coordinates of microbial-like regions of *Arctocephalus gazella*, reference genome GCA_900500725.1. The columns of the BED file have the following notations: ORGANISM—scientific name of the organism, REFID—identification code of the reference genome, CONTIG—identification code of chromosome/scaffold/contig, START—start position of the segment of the microbial-like region, END—end position of the segment of the microbial-like region, LENGTH—length of the segment of the microbial-like region, NREADS—number of pseudo-reads supporting the microbial-like segment, MICR1–3—top 3 most abundant microbes contributing to the microbial-like segment; for example, the element “56_reads_Moritella_sp018219455” within the MICR1 column denotes that *Moritella* sp018219455 was the most abundant microbe from that segment with 56 pseudo-reads

ORGANISM	REFID	CONTIG	START	END	LENGTH	NREADS	MICR1	MICR2	MICR3
*Arctocephalus gazella*	GCA_900500725.1	UIRR01000886.1	127	246	119	49,387	56_reads_*Moritella*_sp018219455	52_reads_*Moritella*_sp018219155	47_reads_*Moritella_marina*
*Arctocephalus gazella*	GCA_900500725.1	UIRR01000886.1	252	895	643	657,342	535_reads_*Moritella*_sp018219455	519_reads_*Vibrio_echinoideorum*	499_reads_*Photobacterium_profundum*_A
*Arctocephalus gazella*	GCA_900500725.1	UIRR01000886.1	1,017	1,222	205	42,914	132_reads_*Moritella*_sp018219455	127_reads_*Photobacterium_swingsii*	124_reads_*Photobacterium_toruni*
*Arctocephalus gazella*	GCA_900500725.1	UIRR01000886.1	1,268	2493	1,225	754,795	945_reads_*Vibrio_parahaemolyticus*	922_reads_*Photobacterium_toruni*	921_reads_*Photobacterium_swingsii*
*Arctocephalus gazella*	GCA_900500725.1	UIRR01000886.1	2,719	5,781	3,062	2,079,142	1,534_reads_*Kosakonia*_sp000410515	1,482_reads_*Photobacterium_toruni*	1,476_reads_*Moritella*_sp018219455
*Arctocephalus gazella*	GCA_900500725.1	UIRR01000886.1	5,878	6,190	312	1,695	24_reads_*Escherichia*_sp004211955	22_reads_*Escherichia_ruysiae*	22_reads_*Escherichia_albertii*

Due to the nature of the PhyloNorway dataset, being the only included dataset with genome-skim assemblies from museum specimens and the potential secondary microbial growth, it exhibited the highest proportions of microbial-like sequences of the genome groups. To validate our method, we therefore extracted the microbial-like (presumed exogenous) and remaining (presumed endogenous) segments from the PhyloNorway reference genomes with *bedtools getfasta* and *bedtools complement* [31] using their coordinates in the BED file. Next, we applied the Mash algorithm [32] (*mash dist* function was used) to construct a matrix of pairwise distances based on their *k*-mer composition among all species, separating the endogenous and exogenous segments. We then computed a principal component analysis (PCA) on the obtained matrix using the *scikit-learn* module in Python. Because microbes and plants have distinct *k*-mer profiles, we used PCA to compare the *k*-mer composition of microbial-like segments identified by our method with that of endogenous segments, thereby aiming to confirm that these groups indeed form distinct clusters.

Finally, our workflow was verified against 2 empirical aeDNA datasets, which capture the flora and fauna from across the Arctic and the Kap Kobenhavn Formation in Greenland, respectively [28, 33]. We used 1 sample from each study: cr9_67 from [28] (further referred to as the “Arctic sample”) and 69_B2_100_L0_KapK-12-1-35 [33] (further referred to as the “Greenland sample”). Adapter-removed reads from these samples were aligned with Bowtie2 [16] to the PhyloNorway reference genome assemblies, together with the Asian elephant as a proxy for woolly mammoths (EleMax1, GCF_024166365.1) and human (GRCH38, GCF_000001405.40) reference genomes. These 2 latter mammalian references were added as decoys to attract mammalian reads via competitive mapping, since mammals were also reported in these samples in the original studies [28, 33]. Next, we used *bedtools closest* [31] to compute the number of intersections of the aligned reads with the microbial-like sequences detected by our workflow in the PhyloNorway reference genomes. A custom R script was used to compute the null distribution of such intersections corresponding to random placement of the reads within the reference genomes.

## Results

**Figure 2:**
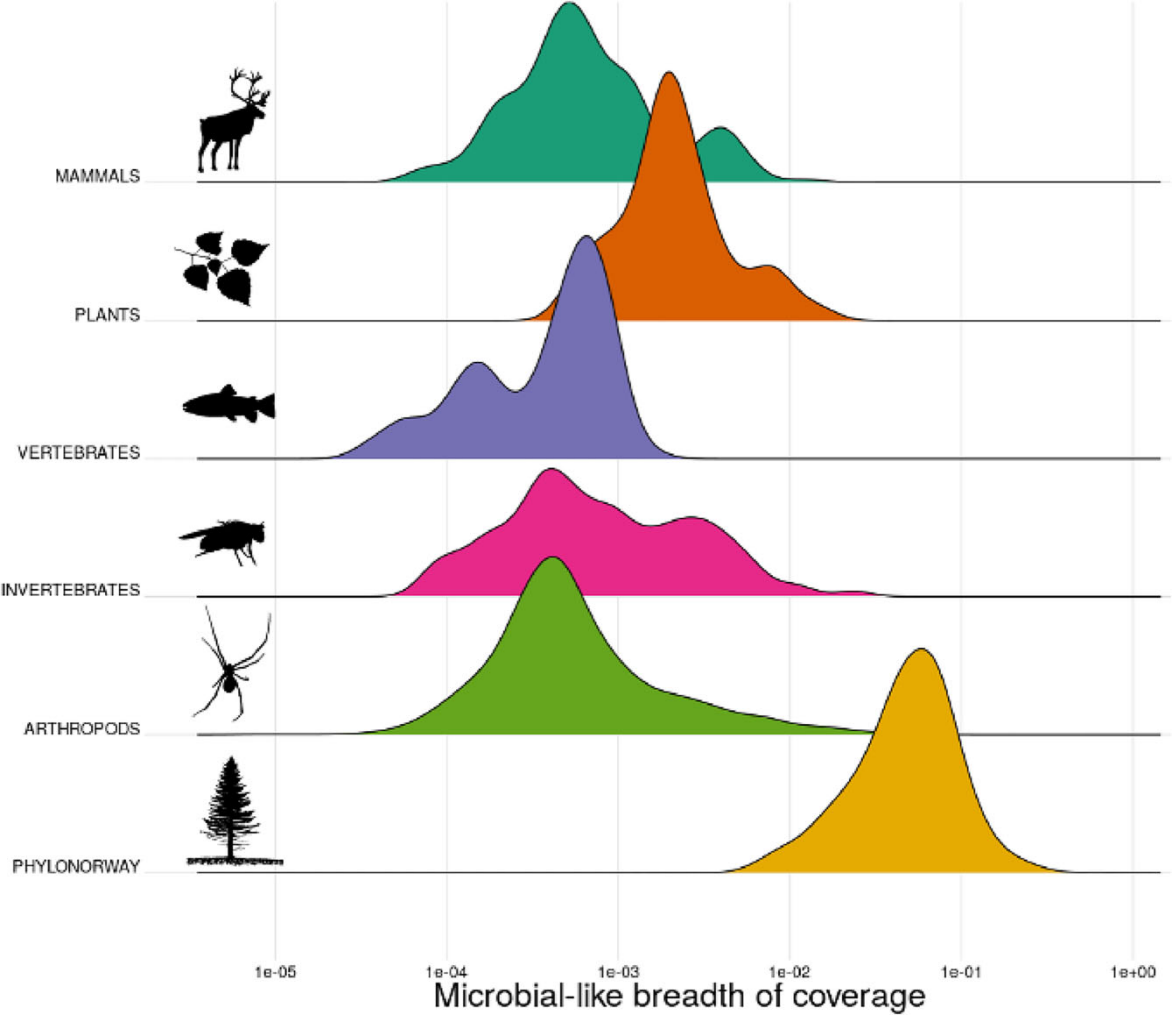
Distribution of microbial-like breadth of coverage (fraction of covered reference nucleotides) across the 6 reference genome groups from PhyloNorway or NCBI RefSeq. Mammalian genomes are represented by the genome with the highest contig N50 for each species sourced from the NCBI assembly database, which includes but is not limited to genomes from Refseq. The x-axis of the plot is on a log-scale. The y-axis represents the density estimates of the 6 datasets.

**Figure 3:**
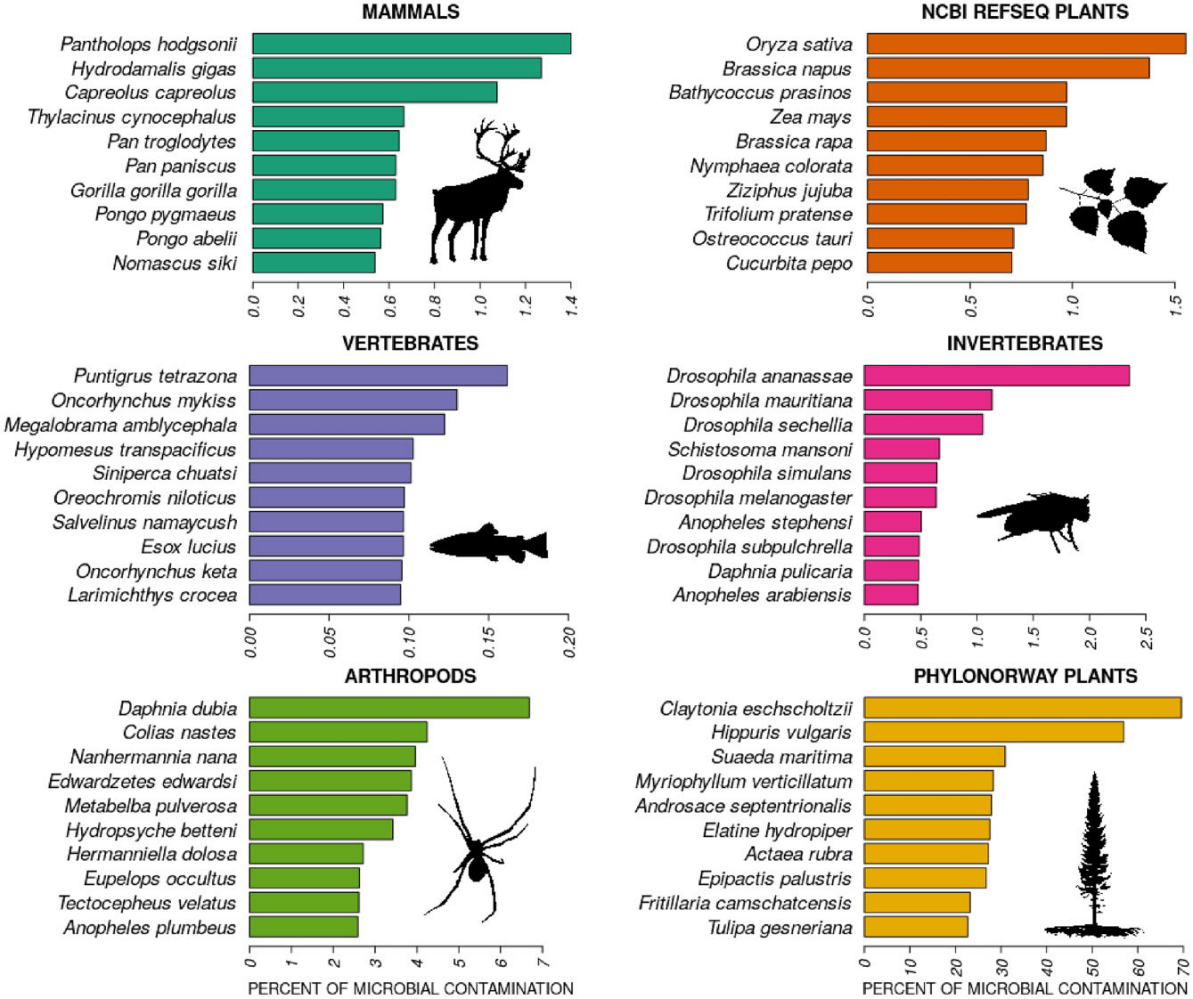
Reference genomes with the highest levels of microbial-like sequences for each genome group. Complete information is available in Supplementary Tables S1–S6.

After applying our workflow to a diverse set of eukaryotic reference genomes, we ranked the results by the percentage of the genome flagged as microbial-like sequence separately for each genome group (Figs. 2 and 3, Supplementary Tables S1–S6).

The nonmammalian vertebrate reference genomes exhibit the lowest overall levels of microbial-like sequence, comprising <0.2% of the reference, as compared to other genome groups, with the Tiger barb fish (*Puntigrus tetrazona*; NCBI id: GCF_018831695.1) having the greatest amount (0.16%). In contrast, mammals, plants, and invertebrate genomes often contained moderate degrees of microbial-like sequence up to ∼2%, where Tibetan antelope (*Pantholops hodgsonii*; GCF_000400835.1; 1.4% microbial-like sequence), rice (*Oryza sativa*; GCF_001433935.1; 1.6%), and fruit-fly (*Drosophila ananassae*; GCA_017639315.2; 2.3%) contain the most microbial-like sequence in each respective group.

During the course of this study, NCBI RefSeq flagged the version of the Tibetan antelope reference genome used here (GCF_000400835.1) as containing a high level of contamination and replaced it with an improved version (GCA_040182635.1). Using our workflow, we found that this reduced the percentage of microbial-like inserts in the Tibetan antelope genome from 1.4% to 0.12%, thereby indirectly validating the accuracy of our approach. Although the improved version of the Tibetan antelope reference genome contains an order of magnitude less microbial-like sequences, we suggest that the remaining microbial-like sequences detected here are likely due to the broader scope of the GTDB microbial dataset we used for the detections. We also found that the only 2 reference genomes available for extinct organisms, Steller’s sea cow (*Hydrodamalis gigas*; GCA_013391785.1; 1.3%) and thylacine (*Thylacinus cynocephalus*; GCA_007646695.3; 0.7%), are among the top 5 mammalian genomes with the most microbial-like sequences (Fig. 3, Supplementary Table S1). This highlights the challenging aspect of high-quality genome assembly from historical samples, as degraded samples have preservation conditions amenable to microbial contamination [34].

Among the mammalian genomes with the most microbial-like sequences, there is a significant overrepresentation of primates. There are 81 primate genomes in the 566 mammalian genomes we assessed (i.e., 14% of the total). Yet, 37 of these contain 0.4–0.6% of microbial-like regions and are within the top 45 genomes (i.e., 82%; Fisher exact test, *P* = 2.6 * 10^−11^), ranked by the fraction of microbial-like sequences (Supplementary Table S1). Similarly, bovids, including cattle (*Bos taurus*; GCA_947034695.1; 0.3%), wild yak (*Bos mutus*; GCA_027580195.1; 0.3%), and American bison (*Bison bison*; GCF_000754665.1; 0.2%), which are common organisms of interest in aeDNA studies, were among the top mammalian organisms with the most microbial-like inserts with up to 9 Mb of their genomes deemed microbial-like.

Among plant reference genomes, rice (*O. sativa*; GCF_001433935.1; 1.6% microbial-like sequences), rapeseed (*Brassica napus*; GCF_020379485.1; 1.4%), corn (*Zea mays*; GCF_902167145.1; 1%), and pumpkin (*Cucurbita pepo*; GCF_002806865.1; 0.7%) have the highest fractions of microbial-like inserts, corresponding to genomic lengths of 2–6 Mb (Fig. 3, Supplementary Table S2). During the period of this study, the rice (*O. sativa*; GCF_001433935.1) reference genome, which we found to have the highest levels of microbial-like sequences, was suppressed by NCBI as a result of standard genome annotation processing, further serving as an independent validation of our microbial-like detection workflow. Invertebrates demonstrate similar levels (i.e., 0.5%–2%), corresponding to genomic lengths of 1–5 Mb, with several *Drosophila* references among the invertebrates with most potentially contaminated reference genomes (Fig. 3 and Supplementary Table S4).

GenBank arthropod reference genomes, which mostly comprise scaffold-level assemblies, on average demonstrate a comparable degree of microbial-like sequences to NCBI RefSeq vertebrates and invertebrates (Fig. 2), although with some outliers, such as the water flea (*Daphnia dubia*; GCA_013387435.1), with ∼7% of microbial-like sequences (7 Mb of genomic length), and the Labrador sulfur butterfly (*Colias nastes*; GCA_907164665.1; 4%; 20 Mb) (Fig. 3 and Supplementary Table S5).

**Figure 4:**
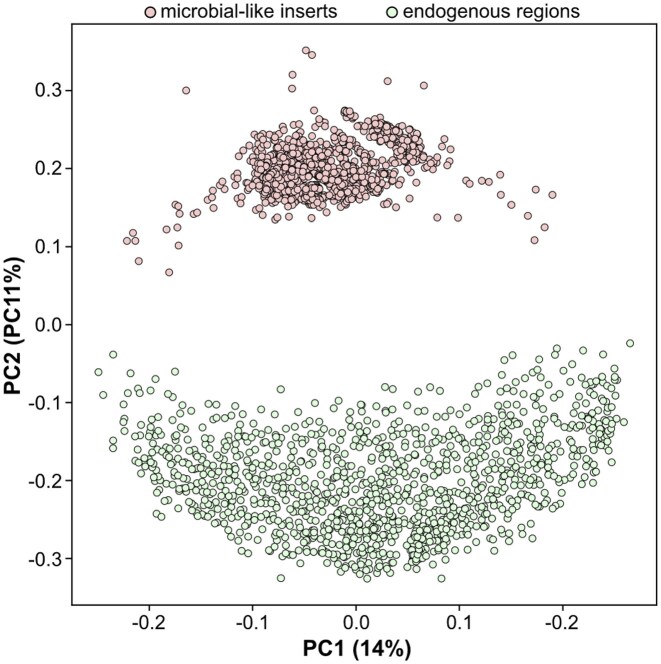
Principal Component Analysis (PCA) visualization of genomic pairwise distances between presumed endogenous (plant DNA) and exogenous (microbial-like) regions in the PhyloNorway dataset detected in this study. Each dot represents a single genome, with the light red dots representing regions identified as microbial-like and green dots as endogenous. The distinct clustering of endogenous and exogenous genomic segments suggests differentiation in their *k*-mer composition.

The PhyloNorway dataset, an extensive collection of high-latitude skimmed plant genomes assembled from herbarium voucher specimens that is currently necessary for ancient environmental metagenomics studies [24, 28, 33], demonstrated particularly high levels of microbial-like sequences (Fig. 2). For instance, the PhyloNorway genomes, with the highest proportions of microbial-like sequences, such as grassleaf spring beauty flower (*Claytonia eschscholtzii*; 70%), common mare’s tail (*Hippuris vulgaris*; 57%), and herbaceous seepweed (*Suaeda maritima*; 31%), were well above the levels observed in other datasets (Fig. 3 and Supplementary Table S6). To further assess the difference in nucleotide composition between endogenous eukaryotic and microbial-like sequences in the PhyloNorway dataset, we performed a PCA based on pairwise *k*-mer matching distances and visualized the 2 leading principal components (Fig. 4). We observed distinct clustering of the microbial-like and endogenous regions, supporting the inference that the identified microbial-like sequences are derived from nonplant sources. In addition, reference sequences of *H. vulgaris*, projected on a hierarchical dendrogram based on pairwise *k*-mer distances between NCBI RefSeq plants and bacteria, demonstrated that microbial-like sequences cluster together with bacterial genomes, whereas endogenous sequences cluster with plant genomes (Supplementary Fig. S3).

The aquatic plant genus *Hippuris* was previously reported as one of the most abundant taxa in ancient sediments from northern Siberia (Arctic sample) [28] and Greenland (Greenland sample) [33]. These identifications were based on alignments to the PhyloNorway reference genome assemblies, in which *H. vulgaris* was the sole representative of the genus. Our analysis revealed that *H. vulgaris* contains one of the highest proportions of microbial-like sequences among the surveyed species. We therefore assessed the extent to which the findings in [28] and [33] may have been influenced by the presence of microbial-like sequences in the *H. vulgaris* reference genome.

The PhyloNorway reference genome assembly of *H. vulgaris* consists of 433,631 contigs, which have a bimodal breadth of coverage distribution for the microbial-like sequence fraction in our analysis, with modes at approximately 0% and 100% (Supplementary Fig. S4). This indicates that a substantial proportion of *H. vulgaris* contigs appear to be free from microbial-like sequences (the zero mode). In the Arctic sample, however, a unimodal distribution of microbial-like fractions from the 20,213 *H. vulgaris* contigs with at least 1 read mapped demonstrates that most of these contigs had close to 100% breadth of coverage of microbial-like sequences (Supplementary Fig. S5A). This implies that the *Hippuris*-identified reads from the Arctic sample have a much higher affinity to the microbial-like *H. vulgaris* contigs, suggesting these reads originated from a microbial source. In contrast, the reads attributed to *H. vulgaris* in the Greenland sample [33] mapped to 73,911 contigs that included both “endogenous” (to a larger extent) and “microbial-like” (to a lesser extent) contigs (Supplementary Fig. S5B). Nevertheless, the peak at 100% of microbial-like fraction is not negligible, implying that the number of endogenous DNA sequences of *H. vulgaris* in the Greenland sample was likely overestimated.

Of the 119,854 reads mapped to the *H. vulgaris* reference for the Arctic sample, 116,483 (i.e., 97%) intersected with regions identified as microbial-like. As our method predicted that the *H. vulgaris* reference comprises 57% microbial-like sequences, we investigated whether the 97% intersect represents a statistically significant enrichment by performing 300 random assignments of the 119,854 reads to the *H. vulgaris* reference within the length limits of each contig. This showed that approximately 58.8% ± 0.3% would be a by-chance expectation if the intersection of mapped reads with the microbial-like regions was random. The observed 97% intersection is beyond the expected percentage (*P* < 0.0033) (Supplementary Fig. S6A). For the Greenland sample, where *Hippuris* was reported to be one of the most abundant genera [33], 1,014,237 of 1,367,627 reads, or 74%, mapped to microbial-like regions of the *H. vulgaris* reference, which was also significantly higher than the null expectation (*P* < 0.0033) (Supplementary Fig. S6B). Therefore, for both the Arctic and Greenland samples, the majority of their reads assigned to *H. vulgaris* are of likely microbial origin. For more details about the *H. vulgaris* analyses, see Supplementary Material S2.

**Figure 5:**
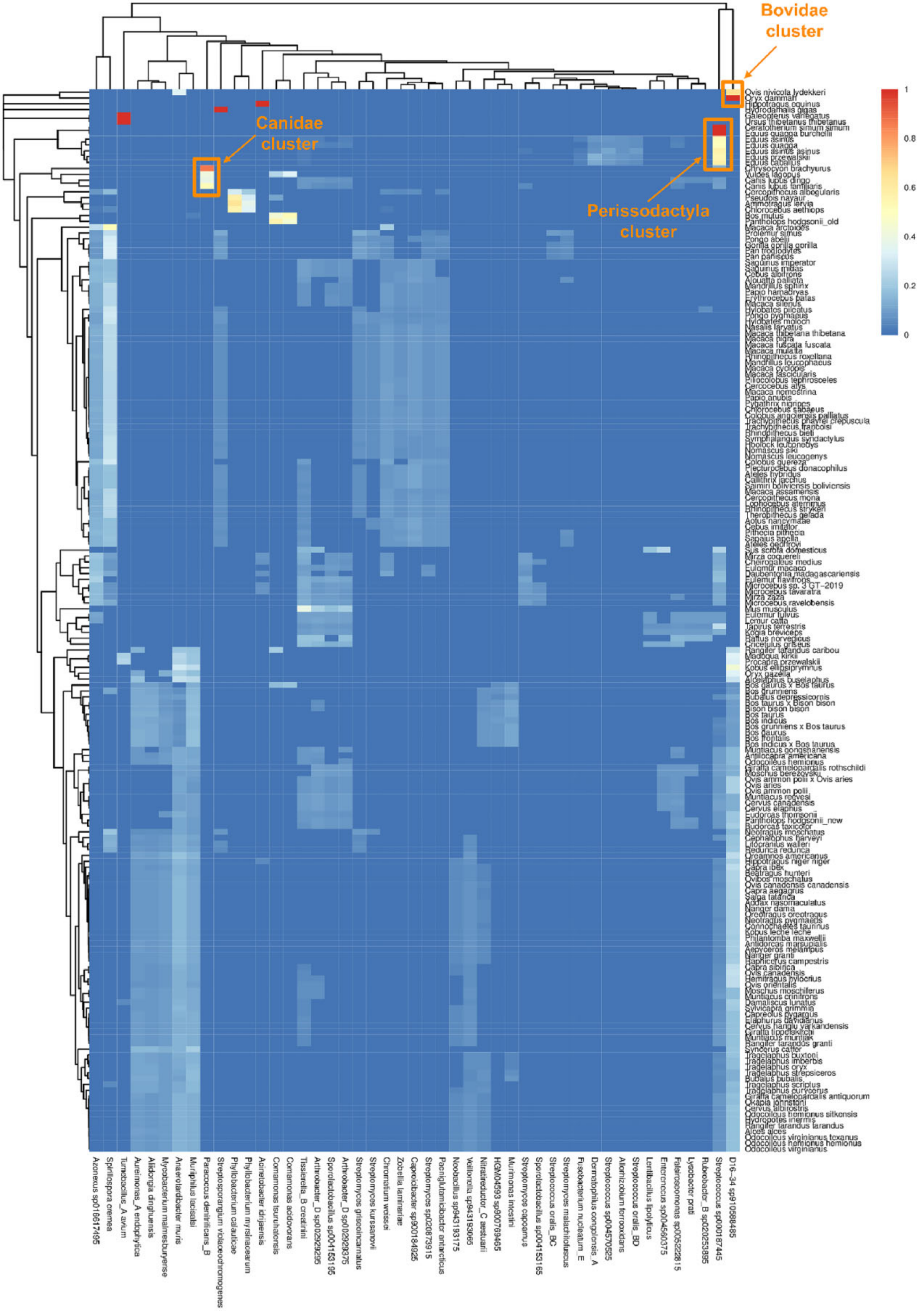
Abundance heatmap of microbial-like sequences across mammalian reference genomes. The columns represent microbial taxa contributing to the reference genomes of mammals displayed as rows. The color gradient indicates normalized abundance of microbial-like sequences (0, lowest; 1, highest).

We next sought to explore the potential mechanisms for the origins of microbial-like sequences in mammalian reference genomes. To achieve this, we quantified the abundance of the most common microbe matches in each eukaryotic reference genome and compared the reference genomes based on the patterns of microbial genus/species presence observed. The most common microbe matches across the mammalian reference genomes with the highest levels of microbial-like sequences form several clusters (Fig. 5). First, the highly abundant *Streptococcus* sp000187445 bacterium is shared across 6 equid reference genomes (*Equus quagga burchellii*, GCA_026770645.1; *Equus przewalskii*, GCF_000696695.1; *Equus caballus*, GCF_002863925.1; *Equus asinus*, GCF_016077325.2; *Equus quagga*, GCF_021613505.1; *Equus asinus asinus*, GCA_003033725.1) and the white rhinoceros (*Ceratotherium simum simum*, GCA_023653735.1). Since these 7 reference genomes were submitted to NCBI by different sequencing centers, lab contamination as a source for the microbial-like sequences is unlikely. The co-occurrence of *Streptococcus* sp000187445 in equids and rhinos is intriguing, as these taxa all comprise part of the odd-toed ungulates, order Perissodactyla. The remaining perissodactyl in the dataset, South American tapir (*Tapirus terrestris*), had the next highest abundance of *Streptococcus* sp000187445 but falls outside of the perissodactyl cluster. This suggests that either *Streptococcus* sp000187445 could be a probiotic microbe endogenous to the perissodactyl microbiome and is erroneously integrated into the genome assemblies, or part of the ancestral perissodactyl genome was evolutionarily convergent with *Streptococcus* sp000187445. Second, the D16-34 sp910588485 bacterium (belonging to genus *Adlercreutzia*) is highly abundant and shared by snow sheep (*Ovis nivicola lydekkeri*, GCA_903231385.1) and scimitar oryx (*Oryx dammah*, GCF_014754425.2) reference genomes, both produced by different centers. These 2 mammalian species belonging to the Bovidae family again suggest some plausible similarity in their microbiomes or alternatively evolutionary convergence. Analogously, reference genomes produced by different centers, of 4 mammalian species belonging to family Canidae—that is, maned wolf (*Chrysocyon brachyurus*, GCA_028533335.1), arctic fox (*Vulpes lagopus*, GCF_018345385.1), dingo (*Canis lupus dingo*, GCF_003254725.2), and domestic dog (*Canis lupus familiaris*, GCF_013276365.1)—all share highly abundant microbial-like sequences from *Paracoccus denitrificans B*, which is a soil-associated bacterium not previously shown to be related to the canid microbiome. Therefore, evolutionary convergence could be an explanation for the co-occurrence of *P. denitrificans B*–like sequences in the reference genomes of Canidae mammals. In addition, at least 2 more large clusters, including broad groups of both mammalian and microbial organisms, can be distinguished: (i) an ungulate cluster driven by intermediately abundant *Aureimonas A endophytica, Aliidongia dinghuensis, Mycobacterium malmesburyense, Anaerotardibacter muris*, and *Muriiphilus lacisalsi* and (ii) a nonhuman primates cluster driven by moderately abundant *Streptomyces griseoincarnatus, Streptomyces kurssanovii, Chromatium weissei, Zobellia laminariae, Caproicibacter* sp900184925, *Streptomyces* sp020873915, and *Paeniglutamicibacter antarcticus*. These 2 clusters suggest that microbial-like sequences from multiple microbes contributed to reference genomes of evolutionarily related organisms, possibly due to shared ecological environments and hence similarities of their microbiomes or evolutionary convergence. In contrast, there are a few clusters that likely point at some commonalities that are not strongly host-associated. For example, *Tumebacillus A avium* is shared at high abundance between Sunda flying lemur (*Galeopterus variegatus*, GCA_004027255.2) and Asian black bear (*Ursus thibetanus thibetanus*, GCA_009660055.1), which are not closely related species, and the reference genomes were produced by different research institutes. Figure 5 also demonstrates that many microbial species, such as *Spirillospora cremea, Azonexus* sp016617495, D16-34 sp910588485, *Anaerotardibacter muris, Chromatium weissei*, and *Aliidongia dinghuensis*, are moderately abundant across a wide range of distinct mammals. Since these microbes are also typical inhabitants of soil and aquatic environments, we hypothesize that they represent environmental or shared lab-reagent contamination, which was incorporated during the sampling, sequencing, and genome assembly process, or can also be due to evolutionary convergence. For further discussion of microbial-like sequence composition within NCBI RefSeq/GenBank plants, invertebrates, nonmammalian vertebrates, arthropods, and PhyloNorway plants, please see Supplementary Material S3 and Supplementary Figs. S7–S11.

## Discussion

Microbial-like sequences present in reference genome databases represent an ongoing problem [35]. While human contamination was recognized some time ago to be one of the major challenges in ancient microbial genomics [9, 36], the opposite scenario of microbial contamination in animal and plant reference genomes became particularly evident in the rapidly developing ancient environmental DNA field [1–4], where reference-based organism discovery is commonplace. Microbial contamination can occur at different steps of reference database generation [19, 20] and subsequently poses a risk of false-positive discovery, which, if unaccounted for, can lead to erroneous results and interpretations in downstream analyses. Previous attempts to address this [12, 13, 17, 19, 20] have concentrated on flagging contaminated eukaryotic references without more comprehensive and quantitative information about specific locations and origins of microbial-like regions. Here, we aimed to mitigate biases introduced by microbial-like sequences with higher precision and mechanistic understanding, concentrating on reference genomes that are particularly important in the field of ancient environmental DNA.

We present a workflow for detecting microbial-like sequences within eukaryotic reference genomes and a collection of BED files (see example in Table 1) with coordinates of microbial-like sequences from a large custom dataset of mammalian, nonmammalian vertebrate, invertebrate, arthropod, and plant reference genomes (*N* = ∼4,300). The application of this workflow allows researchers within the aeDNA field to mask the portions of the genome that match microbial-like sequences. Therefore, rather than merely marking entire reference genomes as unsuitable, our approach seeks to retrieve specific contigs and regions annotated with potentially underlying microbial taxa. The method also enables more precise microbial-like detections by utilizing the largest available microbial genome database (GTDB [27]), which includes both archaeal and bacterial reference genomes.

Although our approach follows a similar strategy put forward by Lu and Salzberg [13] and Steinegger and Salzberg [17], there are a few conceptual and technical differences. Lu and Salzberg [13] implemented splitting of eukaryotic reference genomes into pseudo-reads, screening them with Kraken [14, 15], and aligning them with Bowtie2 [16] against human and microbial references, while Steinegger and Salzberg [17] applied cross-kingdom *k*-mer matching across the NCBI RefSeq, GenBank, and NR databases. In contrast, we follow the opposite approach of splitting microbial (bacterial, archaeal) reference genomes into pseudo-reads and aligning them against eukaryotic references, resulting in precise coordinates of microbial-like regions within eukaryotic reference genomes. The conceptual difference is that only eukaryotic pathogens were used in [13], while we utilize all NCBI RefSeq plant and animal references and the PhyloNorway dataset of skimmed plant genome assemblies. Therefore, our method is not specific to the NCBI databases but applicable to any custom nucleotide sequence in FASTA format. Another conceptual difference is that both [13] and [17] used the microbial NCBI RefSeq database, which has limited size and diversity compared to the nonredundant GTDB database [27] used in our testing (see Supplementary Material S4), which increased detection sensitivity to microbial-like sequences in eukaryotic reference genomes.

As microbial databases like NCBI RefSeq and GTDB are continually updated with new assemblies, masking of eukaryotic reference genomes with BED files from this study should not be considered an exhaustive solution. There will be a need to update the microbial-like regions presented here as microbial databases continue to grow.

The importance of microbial database coverage can be seen from the study by Kjær et al. [33], who used a previous version of GTDB (release 95) as a decoy, in order to ensure that animal and plant hits were not originating from microbial reads. Nevertheless, we report in this study that a substantial amount of sequences attributed to the plant findings in the original work [33] are microbial-like. It is likely that a proportion of microbial-like reads remained in [33] after filtering the data with the GTDB release 95, and further microbial-like sequence discovery became possible with the substantially larger GTDB release 214 database used here.

A potential limitation of our proposed method is the assumption that the GTDB database used in this study represents a microbial “ground truth” and is free from eukaryotic contamination, as prior work raised concerns about the quality of GTDB [37]. However, when we aligned human pseudo-reads (prepared using the same procedure as the microbial pseudo-reads in this study) to over 820 randomly selected GTDB and 25 RefSeq microbial reference genomes, only 3 human reads aligned to any microbial reference, corresponding to a negligible breadth of coverage with a median of 0%, thereby supporting the assumption that it is highly unlikely that the microbial pseudo-reads used in our study contain eukaryotic contamination. In contrast, screening eukaryotic reference genomes revealed substantially higher levels of human-like sequences. For example, the *Spirometra erinaceieuropaei* (parasitic tapeworm) reference genome (GCA_000951995.1) contained over 8 million aligned human pseudo-reads, covering more than 0.1% of the genome with a total of 1.4 Mb of human-like sequences (Supplementary Fig. S15). Similarly, analysis of the *Bathycoccus prasinos* (green algae) reference genome (GCF_002220235.1) revealed over 236,000 aligned human pseudo-reads, covering approximately 0.2% of the genome and spanning a total of 37 kb of human-like regions. This confirms the significantly higher similarity of the eukaryotic pseudo-reads to eukaryotic references compared to bacterial references, which in turn supports the assumption of negligible eukaryotic contamination within GTDB.

To further evaluate the sensitivity and specificity of our workflow, we applied it to a random subset of 6.5 million GTDB pseudo-reads. The screened reference genome consisted of the concatenated hg38 human reference genome and 16 microbial reference genomes (corresponding to 726 reference sequences at the chromosome and scaffold levels) as used in [38]. We observed that only a single microbial pseudo-read aligned to 1 human chromosome (chr 12) out of the 24 canonical and 432 decoy chromosomes in the hg38 reference genome, whereas 202,762 pseudo-reads aligned to the microbial reference sequences. These results highlight the high specificity of the method and indicate a low likelihood of nonspecific alignments by Bowtie2.

There is currently no ultimate bioinformatic solution for distinguishing microbial contamination from true taxonomic hits in aeDNA studies. Here, we emphasize that we can only classify certain regions of eukaryotic reference genomes as “microbial-like,” as there is no guarantee they are of microbial origin and could instead be due to sequence conservation or convergence, or from potential *in vivo* insertion of microbial sequences into eukaryotic genomes.

This challenge is particularly evident when identifying microbial-like sequences in plant reference genomes, such as those in the PhyloNorway dataset. As previously demonstrated [39], the evolutionary relationship between certain bacteria and plant organelles (e.g., chloroplasts) often results in genuine sequence similarities. This overlap can lead to ambiguous classifications and misannotations within databases such as NCBI GenBank. Because the GTDB database includes a subset of cyanobacterial genomes, some of which are among the closest known relatives to plants, we aimed to assess the potential for overestimating microbial-like regions in the PhyloNorway plant reference genomes by our method. First, GTDB version r214, used in our study, contains 3,846 reference genomes from the phylum Cyanobacteriota, approximately 1% of the total 394,932 reference genomes. Second, we specifically evaluated whether cyanobacterial pseudo-reads were overrepresented in the predicted microbial-like regions of 2 plant species from the PhyloNorway dataset, *H. vulgaris* and *C. eschscholtzii*, which had the highest predicted proportions of microbial-like regions (57% and 70%, respectively). According to GTDB r214 annotations, cyanobacterial pseudo-reads accounted for 622,336,764 of 26,089,195,106 total pseudo-reads (2.4%). However, only 2,739,015 cyanobacterial pseudo-reads (0.6%) aligned to *H. vulgaris* and 1,688,444 (0.4%) to *C. eschscholtzii* out of 481,618,468 and 408,824,087 total aligned pseudo-reads, respectively. These results suggest that the cyanobacterial pseudo-reads do not align to the plant reference genomes more frequently than expected by chance. Therefore, while our analysis does not indicate that over-masking plant references due to sequence similarity is a major concern in our workflow, the inherent ambiguity means some microbial-like regions in plant genomes may still be overestimated. However, if such over-masking occurs and these regions represent genuine host genome sequences, microbial sequences in aeDNA samples can still align to them and potentially lead to erroneous taxonomic assignments. In this context, masking these regions remains beneficial, as it promotes a more conservative approach and thus a more reliable detection of true species present in aeDNA data.

Our study highlights the need to avoid using sequencing data mapped to publicly available genomes, without also accounting for microbial-like regions within the reference genome assemblies. When working with only a handful of reference genomes, it is possible to evaluate the contamination of each individually, either through bioinformatic methods or by consulting the methods used to construct each assembly. However, this quickly becomes unfeasible in metagenomic studies, where data are often mapped against hundreds or thousands of different reference genomes. Mapping sequence reads against potentially contaminated reference genomes can lead to spurious detections of animal and plant organisms. We therefore suggest, as a preventive measure, to either mask the microbial-like regions in the reference genomes before performing mapping or add a validation step after mapping to confirm that the detection signal does not derive from the microbial-like regions.

## Availability of Source Code and Requirements

Project name: GENome EXogenous (GENEX) sequence detection

Project homepage: https://github.com/NikolayOskolkov/MCWorkflow

Operating system(s): UNIX

Programming language: bash, R

License: CC0

RRID:SCR_027305 (https://scicrunch.org/resolver/RRID:SCR_027305)

bio.tools ID: genex_workflow (https://bio.tools/genex_workflow)

The source codes of the workflow, together with a comprehensive vignette covering the workflow usage and interpretation of the output, are available at the GitHub repository [40]. Custom scripts used for performing the analysis and computing the figures for the manuscript are explained in detail in Supplementary Material S6 and are available in the GitHub repository [41]. Snapshots of our GitHub repositories are available in Software Heritage [42, 43], and our workflow is also archived in Workflow Hub [44]. The workflow, together with the prebuilt datasets of microbial and human pseudo-reads and other helping files, is also available via the SciLifeLab Figshare repository [45], as well as Zenodo repository [46].

## Additional Files

**Supplementary Fig. S1**. Sensitivity of discovery of microbial-like regions when aligning microbial pseudo-reads to gray short-tailed opossum (*Monodelphis domestica*, GCA_027887165.1) and African elephant (*Loxodonta africana*, GCF_000001905.1) reference genomes with different numbers of multimapping pseudo-reads to retain.

**Supplementary Fig. S2**. Example of coverage of detected exogenous regions by mapped bacterial pseudo-reads to the *Hippuris vulgaris* reference genome from the PhyloNorway dataset. The visualization is performed using the Integrative Genomics Viewer (IGV).

**Supplementary Fig. S3**. *Hippuris vulgaris* microbial-like (presumed exogenous) and remaining (presumed endogenous) segments from the PhyloNorway dataset projected on the hierarchical clustering dendrogram of NCBI RefSeq plants and bacteria computed using Mash [32] pairwise distances based on the *k*-mer composition of their reference genomes.

**Supplementary Fig. S4**. Distribution of microbial-like fractions of 433,631 contigs of *Hippuris vulgaris* from the PhyloNorway dataset profiled in our analysis.

**Supplementary Fig. S5**. Distribution of microbial-like fractions of *Hippuris vulgaris* contigs with aligned reads for (A) Arctic sample cr9_67 [28] (20,213 contigs) and (B) Greenland sample 69_B2_100_L0_KapK-12-1-35 [33] (73,911 contigs).

**Supplementary Fig. S6**. Verification of *Hippuris* hit from [28] and [33]. Intersection fraction of randomly assigned reads from (A) the Arctic sample cr9_67 [28] and (B) the Greenland sample 69_B2_100_L0_KapK-12-1-35 [33], with microbial-like regions in the *Hippuris vulgaris* reference genome.

**Supplementary Fig. S7**. Abundance heatmap of microbial-like sequences across NCBI RefSeq plants. The columns represent microbial taxa contributing to the reference genomes of plants displayed as rows. The color gradient indicates normalized abundance of microbial-like sequences (0, lowest; 1, highest).

**Supplementary Fig. S8**. Abundance heatmap of microbial-like sequences across NCBI RefSeq invertebrates. The columns represent microbial taxa contributing to the reference genomes of invertebrates displayed as rows. The color gradient indicates normalized abundance of microbial-like sequences (0, lowest; 1, highest).

**Supplementary Fig. S9**. Abundance heatmap of microbial-like sequences across NCBI RefSeq nonmammalian vertebrate taxa. The columns represent microbial taxa contributing to the reference genomes of vertebrates displayed as rows. The color gradient indicates normalized abundance of microbial-like sequences (0, lowest; 1, highest).

**Supplementary Fig. S10**. Abundance heatmap of microbial-like sequences across NCBI RefSeq arthropods. The columns represent microbial taxa contributing to the reference genomes of arthropods displayed as rows. The color gradient indicates normalized abundance of microbial-like sequences (0, lowest; 1, highest).

**Supplementary Fig. S11**. Abundance heatmap of microbial-like sequences across PhyloNorway plants. The columns represent microbial taxa contributing to the reference genomes of PhyloNorway plants displayed as rows. The color gradient indicates normalized abundance of microbial-like sequences (0, lowest; 1, highest).

**Supplementary Fig. S12**. Comparison of coverage of a 10-kb region of the sperm whale (*Physeter catodon*, GCA_900411695.1) reference genome by microbial pseudo-reads produced from the microbial RefSeq (top) and microbial GTDB (bottom) databases. The visualization is performed using the Integrative Genome Viewer (IGV).

**Supplementary Fig. S13**. Comparison of coverage of a 1.3-kb region of the African bush elephant (*Loxodonta africana*, GCF_000001905.1) reference genome by microbial pseudo-reads produced from the microbial GTDB (top) and microbial RefSeq (bottom) databases. The visualization is performed using the Integrative Genome Viewer (IGV). The visualization demonstrates that microbial GTDB pseudo-reads are capable of discovering more microbial-like regions within the eukaryotic reference genome compared to microbial RefSeq pseudo-reads.

**Supplementary Fig. S14**. Comparison of numbers of microbial-like regions in mammalian reference genomes detected by using microbial GTDB and RefSeq pseudo-reads. One point represents 1 mammalian reference genome. Red diagonal line highlights equal counts for RefSeq and GTDB.

**Supplementary Fig. S15**. Example of coverage of detected exogenous regions by mapped human pseudo-reads to the *Spirometra erinaceieuropaei* (parasitic tapeworm) reference genome GCA_000951995.1, scaffold LN203398.1 that has 100% breadth of coverage by human pseudo-reads. The visualization is performed using the Integrative Genomics Viewer (IGV).

**Supplementary Table S1**. Mammals.xlsx: Fractions of microbial-like regions in mammalian RefSeq reference genomes.

**Supplementary Table S2**. Plants.xlsx: Fractions of microbial-like regions in plant RefSeq reference genomes.

**Supplementary Table S3**. Vertebrates.xlsx: Fractions of microbial-like regions in non-mammalian vertebrate RefSeq reference genomes.

**Supplementary Table S4**. Invertebrates.xlsx: Fractions of microbial-like regions in invertebrate RefSeq reference genomes.

**Supplementary Table S5**. Arthropods.xlsx: Fractions of microbial-like regions in arthropod RefSeq and GenBank reference genomes.

**Supplementary Table S6**. PhyloNorway.xlsx: Fractions of microbial-like regions in PhyloNorway reference genomes.

giaf108_Supplemental_Files

giaf108_Authors_Response_To_Reviewer_Comments_original_submission

giaf108_Authors_Response_To_Reviewer_Comments_Revision_1

giaf108_GIGA-D-25-00115_original_submission

giaf108_GIGA-D-25-00115_Revision_1

giaf108_GIGA-D-25-00115_Revision_2

giaf108_Reviewer_1_Report_Original_Submission Lucas Czech -- 4/15/2025

giaf108_Reviewer_1_Report_Revision_1 Lucas Czech -- 8/14/2025

giaf108_Reviewer_2_Report_Original_Submission Mikkel Winther Pedersen -- 5/9/2025

giaf108_Reviewer_2_Report_Revision_1 Mikkel Winther Pedersen -- 8/15/2025

## Abbreviations

aeDNA: ancient environmental DNA; GenBank: National Institutes of Health genetic sequence database; GTDB: Genome Taxonomy Database; IGV: Integrative Genomics Viewer; NCBI: National Center for Biotechnology Information; NR: nonredundant; PCA: principal component analysis; RefSeq: Reference Sequence database.

## Data Availability

NCBI RefSeq reference genomes, release 213 (from 23 July 2022), were obtained from [47], and NCBI GenBank reference genomes were downloaded from [48]. The NCBI accession IDs of the reference genomes used for the analysis are available in Supplementary Tables S1–S6. GTDB dataset of microbial reference genome assemblies (bacterial and archaeal), release 214, can be accessed at [49], and the PhyloNorway project DataverseNO V1 Nordic plant contig-level reference genomes are available at [50]. The empirical datasets [28, 32] were obtained from the EMBL-ENA under project accession PRJEB43822 and PRJEB55522, respectively. The adapter-removed reads for the Arctic sample were downloaded from [51], and the adapter-removed reads for the Greenland sample were downloaded from the ftp-address: [52]. The BED-files with coordinates of microbial-like sequences for each group of eukaryotic organisms can be downloaded from the SciLifeLab Figshare repository [53].
