# Supplementary material for: Improving taxonomic inference from ancient environmental metagenomes by masking microbial-like regions in reference genomes
Source: Gigascience. 2025 Oct 3;14:giaf108. doi: 10.1093/gigascience/giaf108 (PMC12491943; doi:10.1093/gigascience/giaf108)
Supplement: giaf108_Supplemental_Files [file giaf108_supplemental_files.zip › Reference_Contamination_Supplementary.docx]

## **Supplementary Material**

### **S1. Alignment of microbial pseudo-reads to eukaryotic reference genomes**

To generate microbial pseudo-reads, we utilized 394,932 bacterial and 7,777 archaeal reference genome sequences from the Genome Taxonomy Database (GTDB) release 214 [27] downloaded from [https://data.gtdb.ecogenomic.org/releases/release214/214.0/](https://data.gtdb.ecogenomic.org/releases/release207/207.0/). Each of the microbial references was fragmented into 60 bp long segments using a sliding window with a 10 bp step. The 60 bp length of microbial pseudo-reads was chosen to provide sufficient specificity of matching to eukaryotic references as it is twice as long as the conventional 30 bp lower threshold of specificity across organisms in the tree of life [14, 15]. As a result, we generated a set of 2.6*10^10^ microbial pseudo-reads. The eukaryotic reference genomes were individually indexed with Bowtie2 [16] using the following command line:

*bowtie2-build --large-index reference_genome.fna.gz reference_genome.fna.gz --threads 20*

Afterwords, microbial pseudo-reads were aligned to each indexed eukaryotic reference genome, and the alignments were sorted and indexed with *samtools* [30] using the following command lines:

*bowtie2 --large-index -f -k 10 -x reference_genome.fna.gz --end-to-end --threads 20 --very-sensitive -U microbial_reads.fna.gz | samtools view -bS -F 4 -h -@ 20 - | samtools sort -@ 20 - > MicrReads_aligned_to_reference_genome.bam*

*samtools index -c MicrReads_aligned_to_reference_genome.bam*

It is reasonable to assume that some microbial sequences can map to multiple loci in eukaryotic reference genomes. Therefore, in order to increase sensitivity of discovery of microbial-like regions, we allowed up to 10 multi-mapping pseudo-reads to be kept in the alignments (the flag *-k 10* in the Bowtie2 command line above). For estimating the optimal number of multi-mappers to keep, we performed alignments of microbial pseudo-reads to Gray short-tailed opossum (*Monodelphis domestica*, GCA_027887165.1) and African elephant (*Loxodonta africana*, GCF_000001905.1) reference genomes while varying the maximum number of multi-mapped positions to retain for a read (0, 5, 10, 25, or 50 positions). We recorded the total number of both mapped reads and discovered regions of microbial-like sequences (Supplementary Figure 1). We observed that both sensitivity metrics for both organisms saturated at ~5-10 multi-mappers. We therefore decided to allow up to 10 multi-mapping pseudo-reads to be kept when performing alignments.

The microbial-like regions were detected by computing the breadth of coverage (boc) from the alignments with *samtools depth* [30] as

*samtools depth -g 0x100 -a MicrReads_aligned_to_reference_genome.bam > boc.txt*

Here, we used the *-g 0x100* flag to account for contributions from multi-mapping microbial pseudo-reads to the total coverage.

It is important to mention that Bowtie2 has a special non-trivial scoring system to determine whether a read will be mapped to a reference. The scoring system is not solely based on the exact number of mismatches but includes multiple other metrics such as base quality, gaps, clipping etc. Nevertheless, Bowtie2 prioritizes high-scoring (i.e. more similar) alignments, and heavily penalizes divergence. Empirically, we can see that a 60 bp read with more than ~6 mismatches (average nucleotide identity ANI=90%) will usually fail to align, even under --very-sensitive –end-to-end. For example, tested on RefSeq plants and PhyloNorway references with aligned GTDB pseudo-reads, we observe on average 4 ± 0.4 and 4 ± 0.2 mismatches per read, respectively, i.e. the similarity of ANI=93%. Taking into account that a typical ANI threshold for ancient metagenomics projects is set much lower, i.e. down to 85%, due to DNA damage [43, 44], we assume the risk of non-specific alignments with the Bowtie2 mapping parameters used in this study is low.

### **S2. Following up the microbial-like and endogenous regions within *Hippuris vulgaris* reference genome assembly from the PhyloNorway dataset**

We used the annotation file *PhyloNorwayContigs_acc2TaxaID.txt* provided together with the PhyloNorway dataset *merged_PhyloNorway.fna* (merged individual FASTA-files) available at <https://doi.org/10.18710/3CVQAG> for retrieving 433,631 contig ids corresponding to the taxid of 39321 of the *Hippuris vulgaris* species. The corresponding reference sequences for each contig id of *Hippuris vulgaris* were extracted with *seqtk subseq* function from the seqtk toolkit <https://github.com/lh3/seqtk>, and saved as *39321.fna* FASTA-file using the following command lines:

*grep -w 39321 PhyloNorwayContigs_acc2TaxaID.txt | cut -f2 > contig_ids_39321.txt*

*seqtk subseq merged_PhyloNorway.fna contig_ids_39321.txt > 39321.fna*

Further, after we have inferred the coordinates of microbial-like regions of *Hippuris vulgaris* with our method, and generated the *micr_coords_39321.bed* BED-file, which can be retrieved from the integrated BED-file for all PhyloNorway reference genomes at SciLifeLab Figshare <https://doi.org/10.17044/scilifelab.28380476>, we proceeded with *bedtools getfasta* [31], and extracted the *Hippuris vulgaris* reference sequences corresponding to the microbial-like regions:

*bedtools getfasta -fi 39321.fna -bed micr_coords_39321.bed -fo micr_seqs_39321.fna*

Next, we applied *samtools* [30], *bedtools complement* [31] and *bedtools getfasta* to group the remaining (presumed endogenous) reference sequences of *Hippuris vulgaris* in a separate FASTA-file:

*samtools faidx 39321.fna && cut -f1,2 39321.fna.fai > 39321.fai*

*bedtools complement -i micr_coords_39321.bed -g 39321.fai > endo_coords_39321.bed*

*bedtools getfasta -fi 39321.fna -bed endo_coords_39321.bed -fo endo_seqs_39321.fna*

In order to explore whether the microbial-like reference sequences of *Hippuris vulgaris* cluster together with bacterial or plant reference genomes, we computed the *k*-mer pairwise distances with Mash [32] using 91 NCBI RefSeq plant and 100 random bacterial NCBI RefSeq reference genomes as well as the two additional *Hippuris vulgaris* FASTA-files corresponding to endogenous and microbial-like sequences. We performed hierarchical clustering with the *hclust* function in R using the Ward method (Supplementary Figure 3). We observed that the inferred microbial-like sequences of *Hippuris vulgaris* were clustering together with bacterial NCBI RefSeq reference genomes while endogenous sequences grouped with plant reference genomes.

Next, for each of 433,631 contigs of *Hippuris vulgaris* we computed the fraction of microbial-like sequences using the coordinates, *micr_coords_39321.bed*, of microbial-like regions. We plotted the histogram, Supplementary Figure 4, of microbial-like fractions with *plot_hist.R* available at <https://github.com/NikolayOskolkov/MCManuscript>.

After we have explored the microbial-like content of the *Hippuris vulgaris* reference genome assembly from the PhyloNorway dataset, we aimed at investigating how this could affect the read assignment in [28] and [33] studies reporting *Hippuris* prevalence at certain periods of history. We downloaded adapter-removed reads in the form of FASTQ-files corresponding to two samples from [28] (“Arctic sample”) and [33] (“Greenland sample”), where high *Hippuris* abundance was reported in the original studies:

*wget* [*ftp://ftp.sra.ebi.ac.uk/vol1/run/ERR645/ERR6458938/cr9_67.truncated.fastq.gz*](http://ftp.sra.ebi.ac.uk/vol1/run/ERR645/ERR6458938/cr9_67.truncated.fastq.gz)

*wget* [*ftp://ftp.sra.ebi.ac.uk/vol1/run/ERR104/ERR10493316/69_B2_100_L0_KapK-12-1-35_Ext-12_Lib-12.pair1.truncated.gz*](http://ftp.sra.ebi.ac.uk/vol1/run/ERR104/ERR10493316/69_B2_100_L0_KapK-12-1-35_Ext-12_Lib-12.pair1.truncated.gz)

Since both mammalian and plant organisms were reported for those two samples in the original studies [28] and [33], we implemented the competitive mapping approach to disentangle the mammalian and plant reads, and proceeded with the reads that align uniquely to the *Hippuris vulgaris* reference. To perform the competitive mapping, we built Bowtie2 [16] index of the *Hippuris vulgaris* reference genome concatenated with Asian Elephant (EleMax1, GCF_024166365.1) and Human (GRCH38, GCF_000001405.40) reference genome. Next, we performed Bowtie2 alignment of the downloaded reads to the indexed composite reference, and extracted only the reads mapping uniquely to the *Hippuris vulgaris* reference genome:

*cat EleMax1.fna Human38.fna 39321.fna > EleMax_Human_Hippuris.fna*

*bowtie2-build --large-index EleMax_Human_Hippuris.fna EleMax_Human_Hippuris.fna --threads 20*

*bowtie2 --large-index -x EleMax_Human_Hippuris.fna --end-to-end --very-sensitive --threads 20 -U* [*cr9_67.truncated.fastq.gz*](http://ftp.sra.ebi.ac.uk/vol1/run/ERR645/ERR6458938/cr9_67.truncated.fastq.gz) *| samtools view -bS -q 1 -h -@ 20 - | samtools sort -@ 20 - > cr9_67.aligned_to_EleMax_Human_Hippuris.bam*

*awk '{print $1, 1, $2}' OFS='\t' genome_39321.fna.fai > genome_39321.fna.bed*

*samtools view -L genome_39321.fna.bed -q 1 -h -@ 20 -o cr9_67.aligned_to_39321.bam cr9_67.aligned_to_EleMax_Human_Hippuris.bam*

From the alignment BAM-file, we retrieved the ids of contigs with at least one read aligned, and using the BED-coordinates, *micr_coords_39321.bed*, of microbial-like regions for *Hippuris vulgaris*, we computed the fraction of microbial-like sequences corresponding to each contig with at least one aligned read (Supplementary Figure 5).

To understand how often the aligned reads overlap with the inferred microbial-like regions of *Hippuris vulgaris*, we extracted the coordinates of aligned reads with *bedtools bamtobed*:

*bedtools bamtobed -i cr9_67.aligned_to_39321.bam > cr9_67.coords_aligned_reads.bed*

and calculated the number of intersections between the coordinates of the aligned reads and the coordinates of inferred microbial-like regions using *bedtools closest* with the *-d* (report distance) flag and custom bash / awk command lines:

*bedtools closest -a cr9_67.coords_aligned_reads.bed -b micr_coords_39321.bed -d > cr9_67.coords_aligned_reads_annotated_with_closest_micr_like_region.bed*

*cut -f7 cr9_67.coords_aligned_reads_annotated_with_closest_micr_like_region.bed | awk '{if($1==0)print $0}' | wc -l >> number_of_observed_intersects.txt*

We discovered that the vast majority of aligned reads, i.e. 116,483 out of 119,854 reads mapped in the Arctic sample (i.e. 97%) and 1,014,237 out of 1,367,627 reads (i.e. 74%) in the Greenland sample, intersected with the regions previously identified as microbial-like in the *Hippuris vulgaris* reference. To check whether this represents a significant enrichment compared to random read positioning, we performed 300 random replacements of the aligned reads, and every time counted the number of their intersects with the coordinates of microbial-like regions using a custom R script, please see the whole procedure in the R script *shuffle_reads.R* available at <https://github.com/NikolayOskolkov/MCManuscript>. We produced the Supplementary Figure 6 using the recorded numbers of intersects between the randomly placed reads and microbial-like regions and plotted them with *plot_hist.R* script.

### **S3. Microbial-like sequence composition of reference genomes from NCBI RefSeq plants, invertebrates, non-mammalian vertebrates, arthropods and PhyloNorway plants**

We used samtools [30] and custom bash and R scripts for annotating the eukaryotic reference genomes with microbial taxonomic names corresponding to the most abundant microbial-like sequences. The most abundant (top 10 for each organism) microbes and eukaryotic references with the highest levels (top 200) of microbial-like regions were summarized via a heatmap computed by the *pheatmap* R package, demonstrating microbial co-occurrence in some groups of mammalian organisms (Figure 5). By analogy with the mammalian microbial-like sequences abundance heatmap, similar clustering patterns can be observed in microbial-like sequence composition of NCBI RefSeq plants, invertebrates, non-mammalian vertebrates, arthropods and PhyloNorway plants, shown respectively in Supplementary Figures 7-11.

For example, *Stenotrophomonas* sp003504055 is shared at high and moderately high abundance across two clusters comprising the fruit fly genus *Drosophila* (Supplementary Figure 8). Similarly, for non-mammalian vertebrate taxa, *Methylocystis* sp011058845 is highly abundant and shared across freshwater fishes such as northern pike (*Esox lucius*, GCF_011004845.1), lake whitefish (*Coregonus clupeaformis*, GCF_020615455.1), lake trout (*Salvelinus namaycush*, GCF_016432855.1), Atlantic salmon (*Salmo salar*, GCF_905237065.1), brown trout (*Salmo trutta*, GCF_901001165.1), chum salmon (*Oncorhynchus keta*, GCF_012931545.1), rainbow trout (*Oncorhynchus mykiss*, GCF_013265735.2), coho salmon (*Oncorhynchus kisutch*, GCF_002021735.2), sockeye salmon (*Oncorhynchus nerka*, GCF_006149115.2), pink salmon (*Oncorhynchus gorbuscha*, GCF_021184085.1) and chinook salmon (*Oncorhynchus tshawytscha*, GCF_018296145.1) (Supplementary Figure 9).

There are also a few clear clusters of arthropod reference genomes that share common microbial-like sequences. For instance, *Enterobacter* sp000493015 is commonly present among reference genomes of butterflies, moths and wasps such as Labrador sulphur (*Colias nastes*, GCA_907164665.1), Asiatic rice borer (*Chilo suppressalis*, GCA_902850365.2), parasitic wasp (*Cotesia vestalis*, GCA_000956155.1), and queen butterfly (*Danaus gilippus*, GCA_018231785.1), whereas *Sphingomonas* sp017418975 is prevalent and shared in reference genomes of soil and leaf associated arthropods such as beetle mite (*Nanhermannia comitalis*, GCA_034697665.1), oribatid mites (*Nothrus palustris*, GCA_034697745.1; *Malaconothrus monodactylus*, GCA_034697245.1), terrestrial cave isopod (*Haplophthalmus danicus*, GCA_034700045.1) and springtail (*Isotomurus plumosus*, GCA_034696705.1) (Supplementary Figure 10).

In contrast to the NCBI reference genomes, the PhyloNorway dataset does not demonstrate obvious commonalities in terms of co-occurrence of microbial-like sequences. Instead, there is at least one group of microbes including *JC017* sp004296775, *Solirubrobacter* sp003344625, *Frankia californiensis*, *Frankia* sp917627385, *Frankia meridionalis*, *Geodermatophilus endophyticus_A*, *Spirillospora cremea*, *Modestobacter lapidis*, *Geodermatophilus* sp019799925, *Streptomyces capoamus*, *SACZ01* sp023369685, *Ancylomarina* sp009669305, which is shared across nearly all plant genome assemblies in the PhyloNorway dataset (Supplementary Figure 11). This reflects, in our opinion, the common sample storage, processing, and sequencing routines used for generating these genome assemblies rather than shared ecological or evolutionary factors.

### **S4. Discovering microbial-like regions with microbial RefSeq pseudo-reads**

In addition to the microbial pseudo-reads produced from the GTDB database, which included only bacterial and archaeal reference genomes, we have also generated a set of 1.1*10^10^ nucleotide sequences using the NCBI RefSeq microbial database, release 213. The latter contained 39,760 microbial reference genomes including 28,044 bacteria, 11,220 viruses, 459 archaea, 33 fungi and 4 protozoa. The RefSeq microbial pseudo-reads were prepared in the same way as described in the Methods section. The accuracy of RefSeq pseudo-reads preparation was validated by aligning them to 25 randomly selected RefSeq reference sequences which yielded a median breadth of coverage of 97.2%, which supports our expectation that the RefSeq reference sequences looked composed almost entirely of microbial-like sequences. Despite the potential redundancy (e.g. some bacteria such as *Escherichia coli* may have multiple versions of a reference genome), the RefSeq microbial pseudo-reads may be useful for discovering viral-like sequences in eukaryotic reference genomes. This analysis can be used complementary to the detection of microbial-like sequences with the GTDB pseudo-reads within the main workflow. Both GTDB and RefSeq microbial pseudo-reads are publicly available together with the workflow files via the SciLifeLab Figshare <https://doi.org/10.17044/scilifelab.28380476>. We found that in most cases, either the coverage by GTDB and RefSeq pseudo-reads had good agreement (Supplementary Figure 12), or the GTDB pseudo-reads provided higher resolution of discovery of microbial-like sequences (Supplementary Figures 13 and 14). Nevertheless, viral-like regions within eukaryotic genomes can only be inferred using the RefSeq microbial pseudo-reads.

When using this workflow with RefSeq (viral) pseudo-reads, it is important to carefully assess genomic fragments classified as viral-like sequences, as they may not represent free-living viral contaminants, but rather endogenous viral elements (EVEs), which are "fossilised" viral sequences integrated into the host genome. Establishing EVEs is a challenging problem and requires careful analysis to confirm that these sequences are not of exogenous viral origin [37]. Our approach can be used for detecting only recent EVEs, as our workflow relies on a mapping tool that performs poorly with highly divergent DNA sequences [38], a common feature of EVEs. Homology-based methods therefore offer a more effective alternative for detecting distant viral relationships due to their greater flexibility and sensitivity [37, 39, 40].

### **S5. Discovering human-like regions with human hg38 pseudo-reads**

We have pre-computed human hg38 pseudo-reads, which resulted in a set of 3.2*10^8^ nucleotide sequences, and made them publicly available together with the workflow (see also Data and Code Availability). The workflow parameters have been updated to include an option for using these pre-computed human pseudo-reads, enabling users to detect “human-like” regions in prokaryotic or eukaryotic reference genomes. As a proof of concept, we applied the workflow to the *Spirometra erinaceieuropaei* (parasitic tapeworm) reference genome GCA_000951995.1, previously suspected of containing human contamination [9]. Our analysis revealed that more than 0.1% of the genome contains human-like sequences, including over 50 scaffolds—some up to 1.7 kbp in length—with 100% breadth of coverage by human pseudo-reads. The total length of detected human-like sequences amounts to 1.4 Mbp. An IGV visualization of one of the fully covered scaffolds is shown in the Supplementary Figure 15. In addition, screening the *Bathycoccus prasinos* (green algae) reference genome GCF_002220235.1 revealed over 236,000 aligned human pseudo-reads, covering approximately 0.2% of the genome. The total length of these potentially exogenous regions amounts to 37 kbp. This testing demonstrates that the workflow can be extended beyond discovering only microbial-like sequences and serve as a tool for detecting exogenous regions within a given reference in general.

### **S6. Scripts used for computing main and supplementary figures**

All scripts and input files used in this study for computing main and supplementary figures are available at the GitHub repository <https://github.com/NikolayOskolkov/MCManuscript>. Main Figures 2, 3, 4 and 5 were plotted in R using *ridgeline.R*, *make_cont_barplots.R*, *plotPCA.py* and *micr_abund_heatmap.R* scripts, respectively. Supplementary Figures 1 and 3 were produced using *multimappers.R* and *cluster_plants_plus_bacteria_plus_hippuris.R*, respectively. The output of the latter script, i.e. the dendrogram in Newick format, is available at the GitHub as *dendrogram.nwk* file. Supplementary Figures 4-6 were plotted in R using *plot_hist.R* script. The heatmaps for Supplementary Figures 7-11 were computed with *micr_abund_heatmap.R* script. The input files for computing the heatmaps are available in the *micr_abundance* folder in the GitHub repository. Finally, the Supplementary Figure 14 was calculated in R with *RefSeq_vs_GTDB_discovered_regions.R* script.

### **Supplementary References**

[37] Brait N, Hackl T, Lequime S., detectEVE: Fast, Sensitive and Precise Detection of Endogenous Viral Elements in Genomic Data. Mol Ecol Resour. 2025 Feb 12:e14083. doi: 10.1111/1755-0998.14083. Epub ahead of print. PMID: 39936183.

[38] Stephanie Dolenz, Tom van der Valk, Chenyu Jin, Jonas Oppenheimer, Muhammad Bilal Sharif, Ludovic Orlando, Beth Shapiro, Love Dalén, Peter D Heintzman, Unravelling reference bias in ancient DNA datasets, *Bioinformatics*, Volume 40, Issue 7, July 2024, btae436, <https://doi.org/10.1093/bioinformatics/btae436>

[39] Blanco-Melo D, Campbell MA, Zhu H, Dennis TPW, Modha S, Lytras S, Hughes J, Gatseva A, Gifford RJ. A novel approach to exploring the dark genome and its application to mapping of the vertebrate virus fossil record. Genome Biol. 2024 May 13;25(1):120. doi: 10.1186/s13059-024-03258-y. PMID: 38741126; PMCID: PMC11089739.

[40] Palatini U, Alfano N, Carballar-Lejarazu R, Chen XG, Delatte H, Bonizzoni M. Virome and nrEVEome diversity of Aedes albopictus mosquitoes from La Reunion Island and China. Virol J. 2022 Nov 18;19(1):190. doi: 10.1186/s12985-022-01918-8. Erratum in: Virol J. 2022 Dec 9;19(1):211. doi: 10.1186/s12985-022-01950-8.

[44] Hübler R, Key FM, Warinner C, Bos KI, Krause J, Herbig A. HOPS: automated detection and authentication of pathogen DNA in archaeological remains. Genome Biol. 2019 Dec 16;20(1):280. doi: 10.1186/s13059-019-1903-0. PMID: 31842945; PMCID: PMC6913047.
