# Supplementary material for: Improving taxonomic inference from ancient environmental metagenomes by masking microbial-like regions in reference genomes
Source: Gigascience. 2025 Oct 3;14:giaf108. doi: 10.1093/gigascience/giaf108 (PMC12491943; doi:10.1093/gigascience/giaf108)
Supplement: giaf108_Supplemental_Files [file giaf108_supplemental_files.zip › Supplementary Material.pdf]

Supplementary Figures

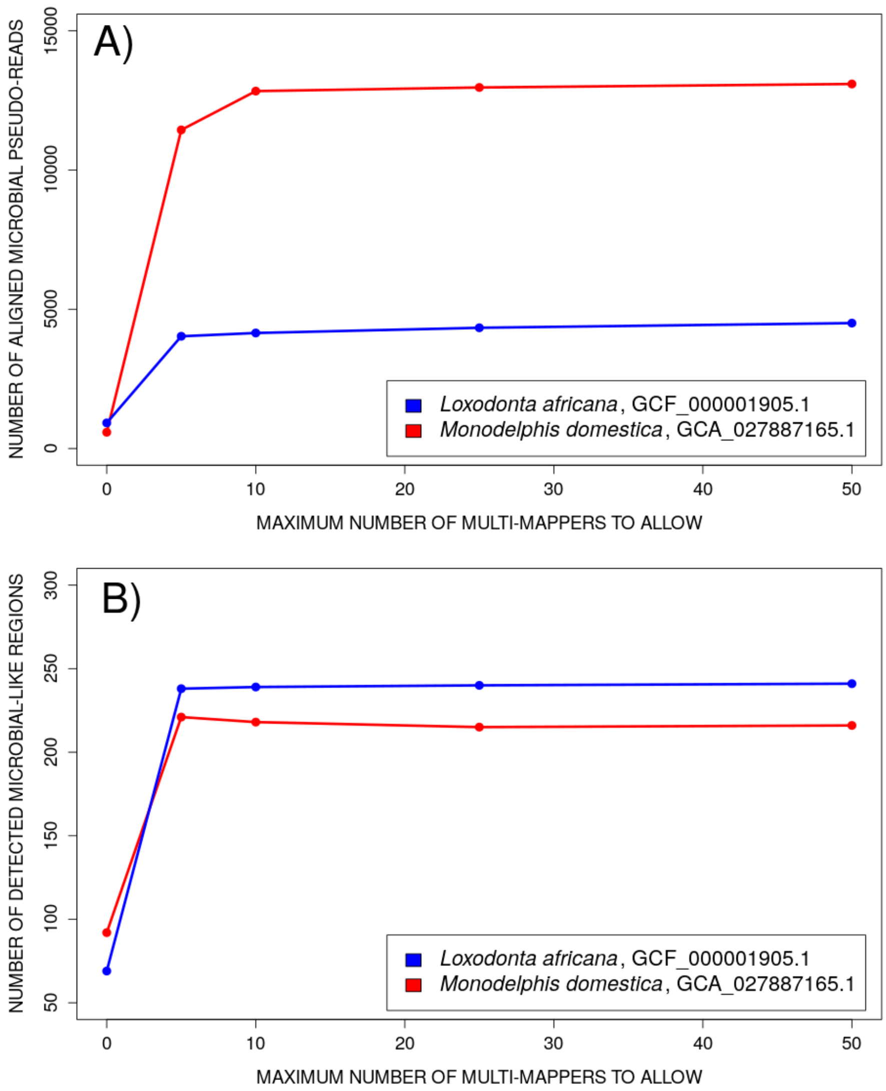

Supplementary Figure 1. Sensitivity of discovery of microbial-like regions when aligning microbial pseudo-reads to Gray short-tailed opossum (*Monodelphis domestica*, GCA_027887165.1) and African elephant (*Loxodonta africana*, GCF_000001905.1) reference genomes with different numbers of multi-mapping pseudo-reads to retain.

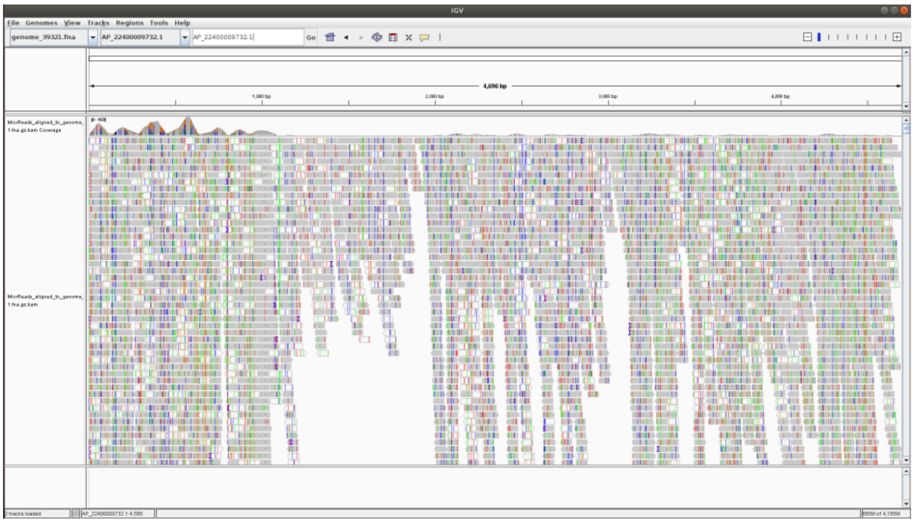

Supplementary Figure 2. Example of coverage of detected exogenous regions by mapped bacterial pseudo-reads to the *Hippuris vulgaris* reference genome from the PhyloNorway dataset. The visualization is performed using the Integrative Genomics Viewer (IGV).

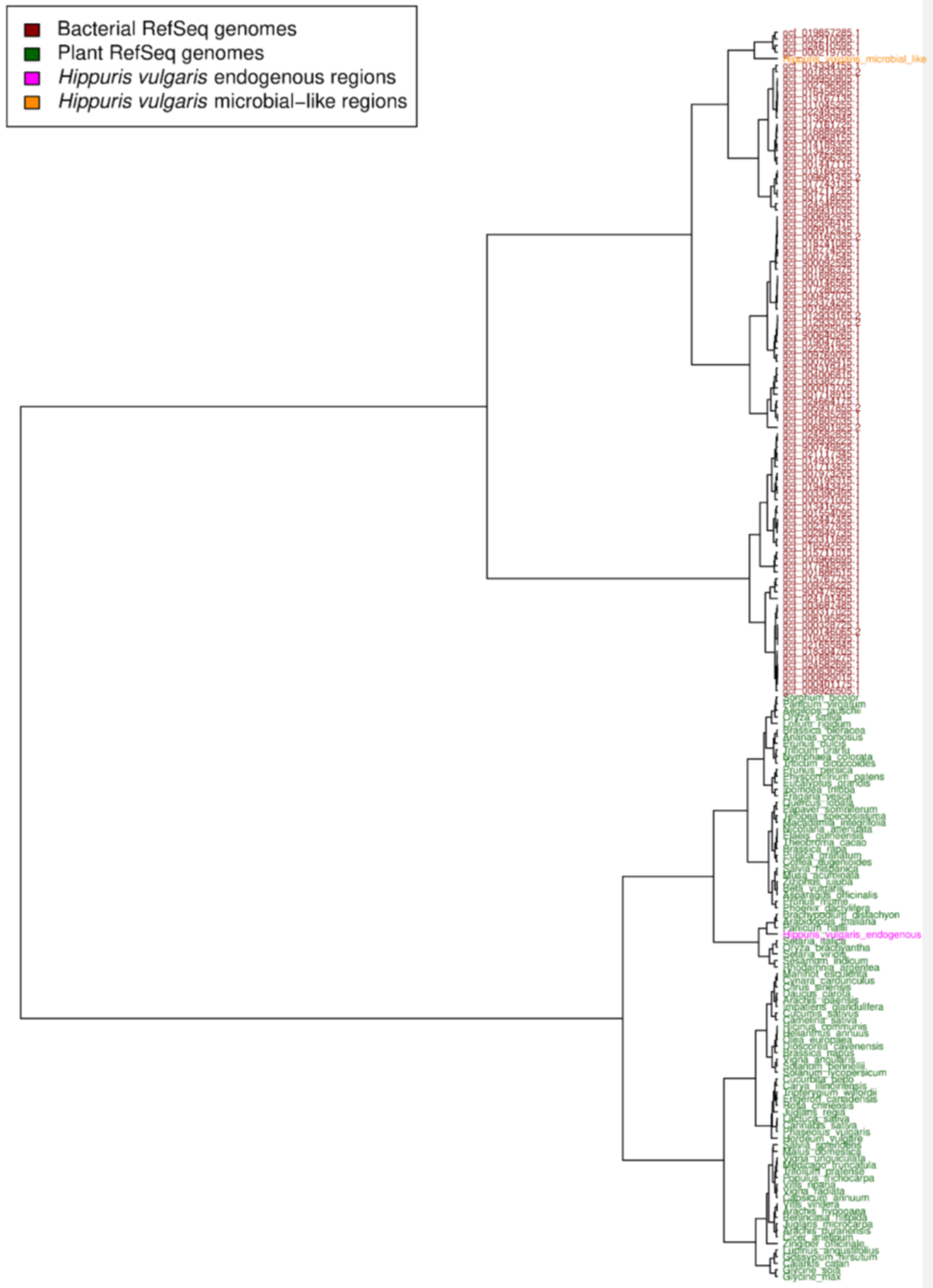

Supplementary Figure 3. *Hippuris vulgaris* microbial-like (presumed exogenous) and remaining (presumed endogenous) segments from the PhyloNorway dataset projected on the hierarchical clustering dendrogram of NCBI RefSeq plants and bacteria computed using Mash [32] pairwise distances based on the *k*-mer composition of their reference genomes.

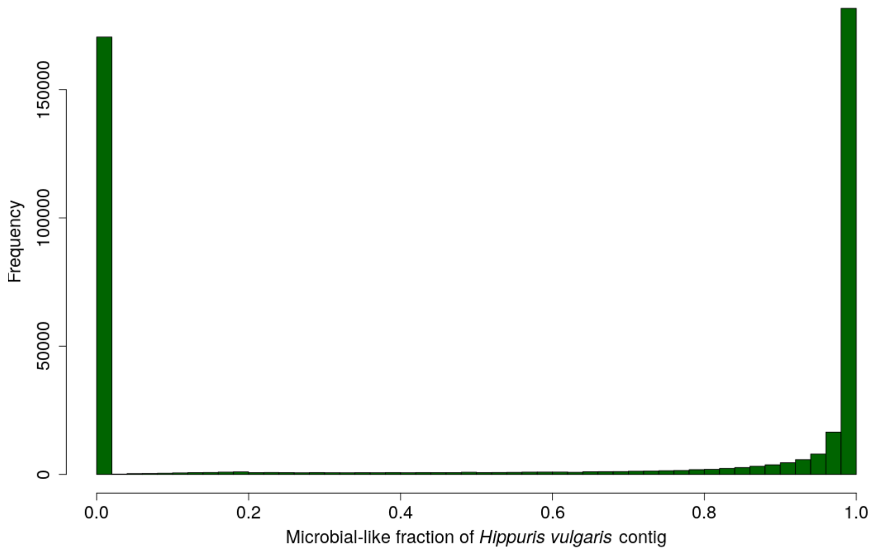

Supplementary Figure 4. Distribution of microbial-like fractions of 433,631 contigs of *Hippuris vulgaris* from the PhyloNorway dataset profiled ~~for microbial contamination~~ in our analysis.

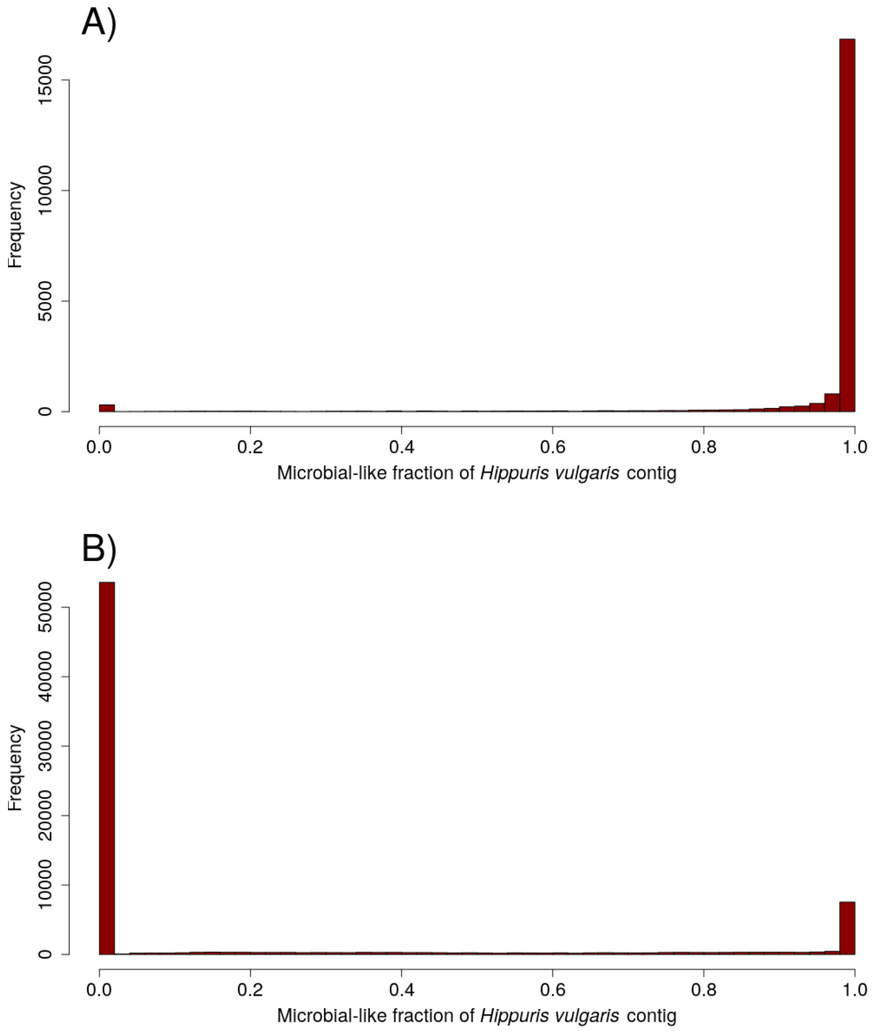

Supplementary Figure 5. Distribution of microbial-like fractions of *Hippuris vulgaris* contigs with aligned reads for: A) Arctic sample cr9_67 [28] (20,213 contigs), and B) Greenland sample 69_B2_100_L0_KapK-12-1-35 [33] (73,911 contigs).

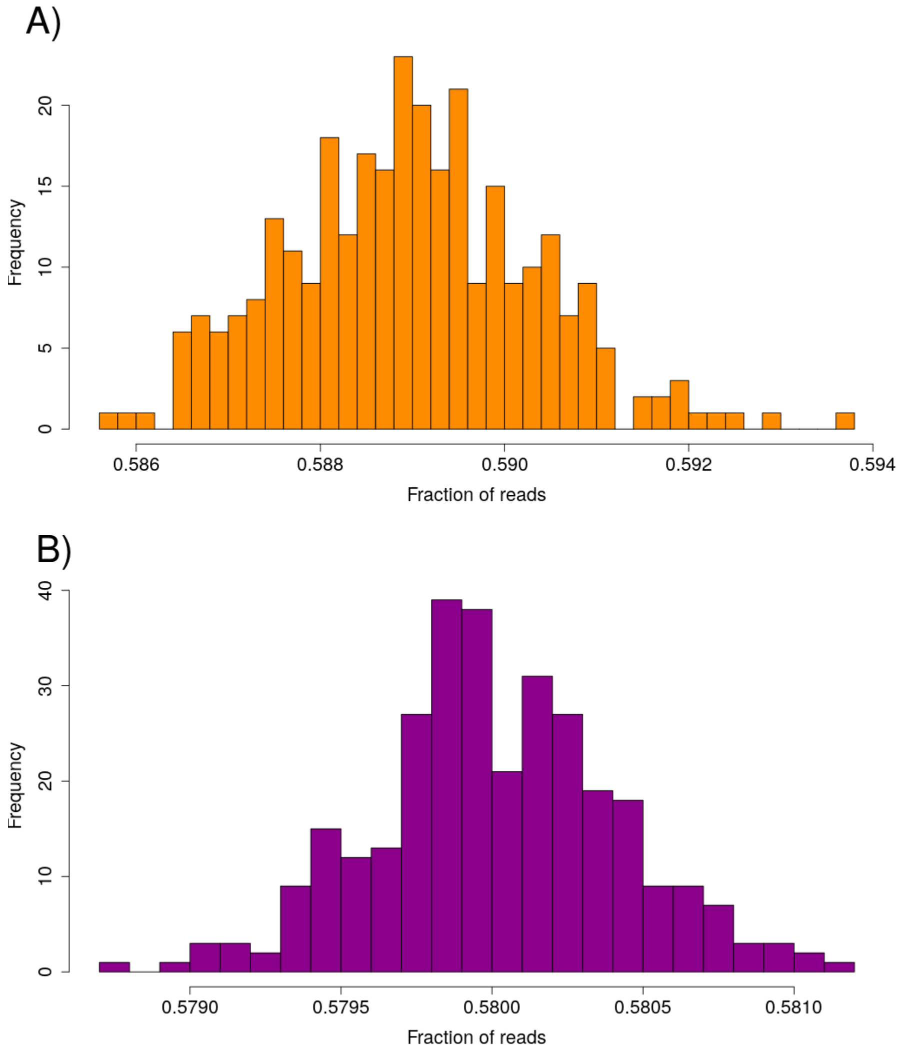

Supplementary Figure 6. Verification of *Hippuris* hit from [28] and [33]. Intersection fraction of randomly assigned reads from: A) the Arctic sample cr9_67 [28], and B) the Greenland sample 69_B2_100_L0_KapK-12-1-35 [33], with microbial-like regions in the *Hippuris vulgaris* reference genome.

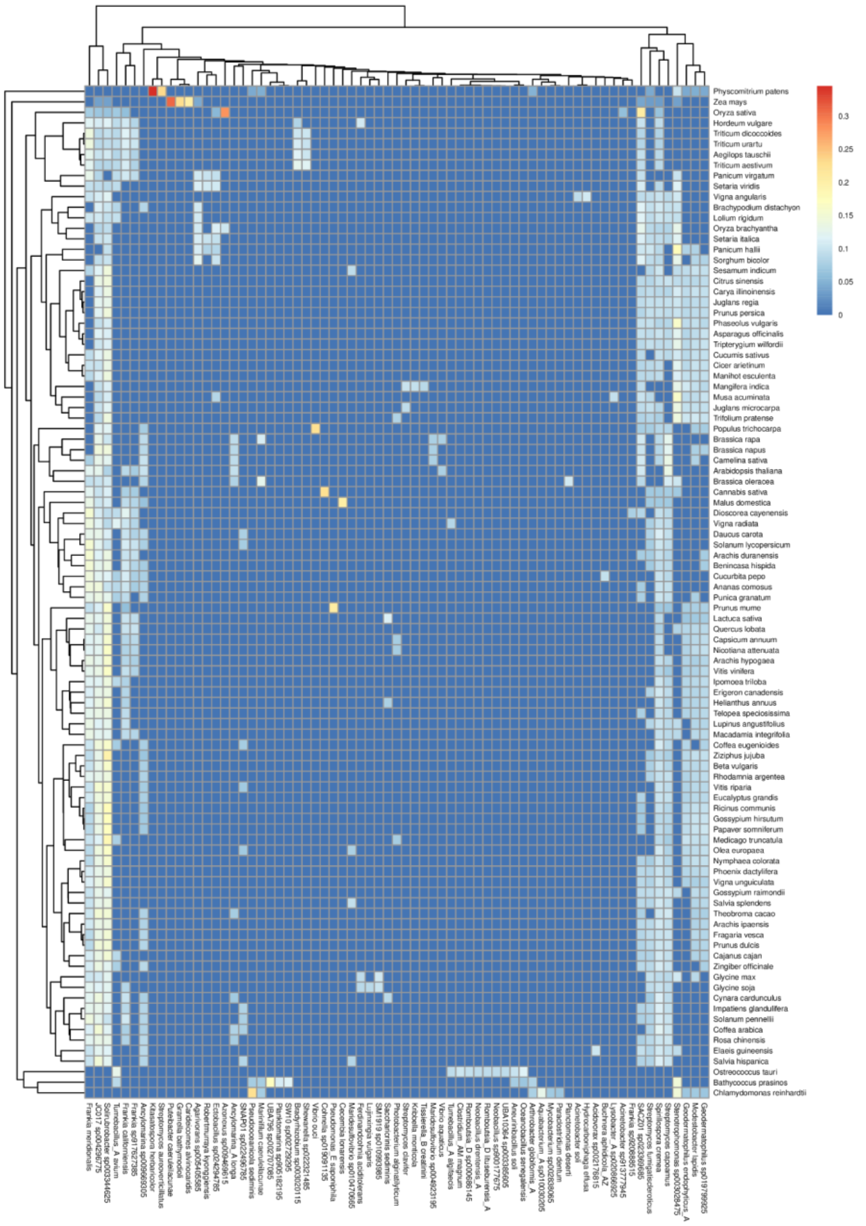

Supplementary Figure 7. Abundance heatmap of microbial-like sequences across NCBI RefSeq plants. The columns represent microbial taxa contributing to the reference genomes of plants displayed as rows. The color gradient indicates normalized abundance of microbial-like sequences (0-lowest, 1-highest).

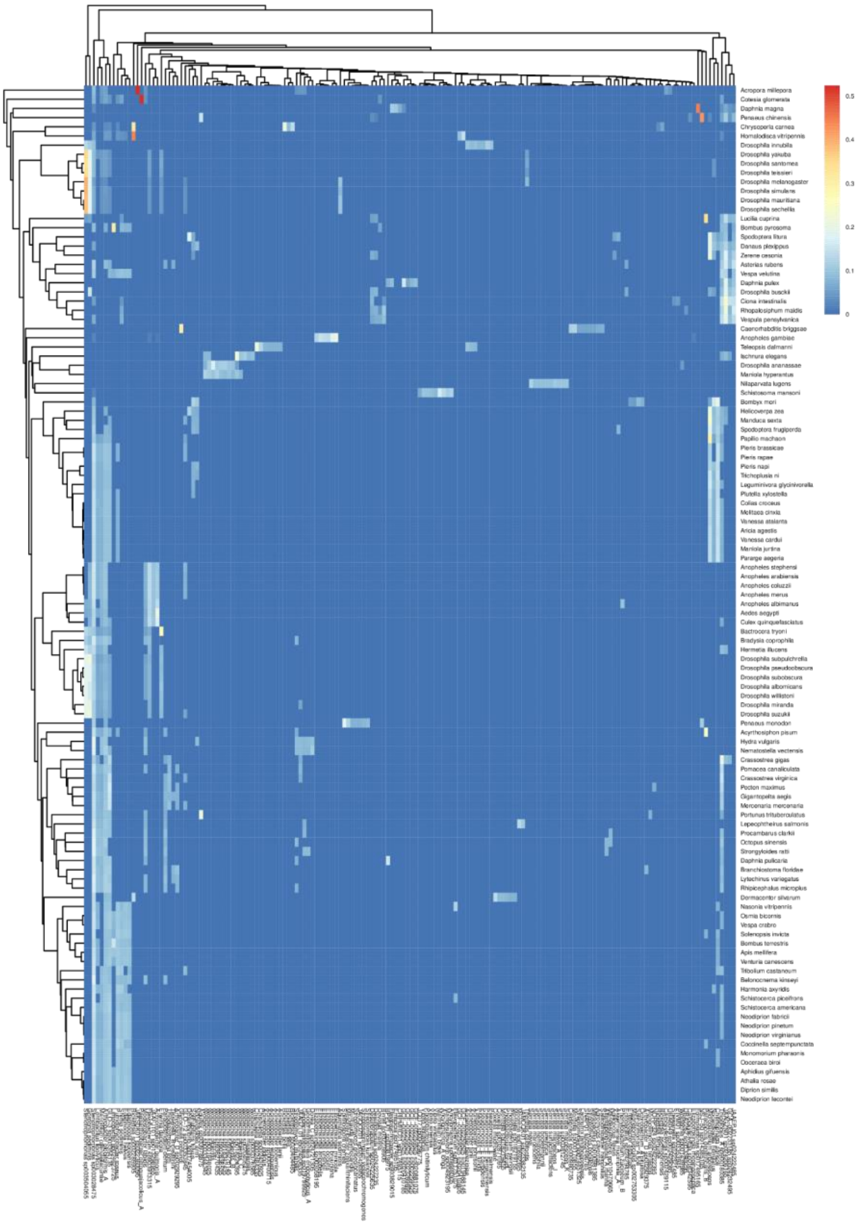

Supplementary Figure 8. Abundance heatmap of microbial-like sequences across NCBI RefSeq invertebrates. The columns represent microbial taxa contributing to the reference genomes of invertebrates displayed as rows. The color gradient indicates normalized abundance of microbial-like sequences (0-lowest, 1-highest).

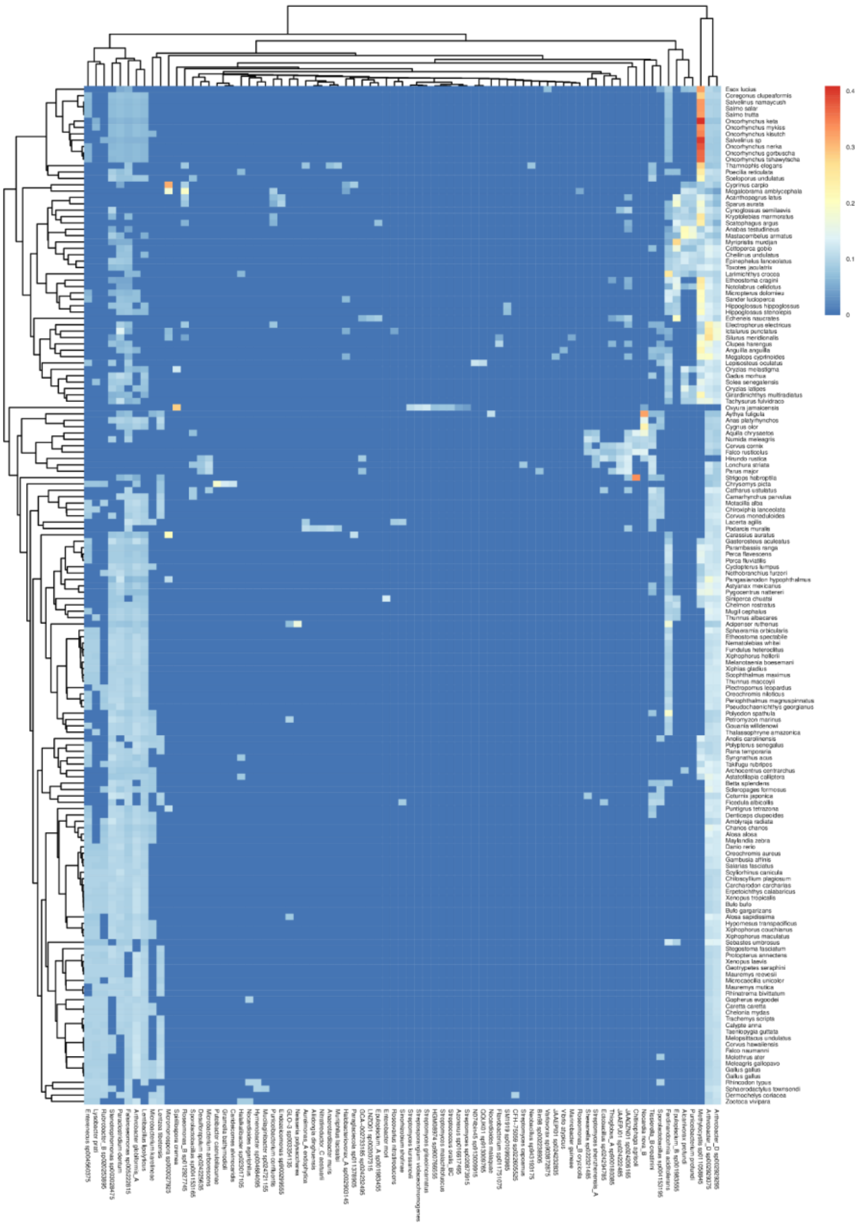

Supplementary Figure 9. Abundance heatmap of microbial-like sequences across NCBI RefSeq non-mammalian vertebrate taxa. The columns represent microbial taxa contributing to the reference genomes of vertebrates displayed as rows. The color gradient indicates normalized abundance of microbial-like sequences (0-lowest, 1-highest).

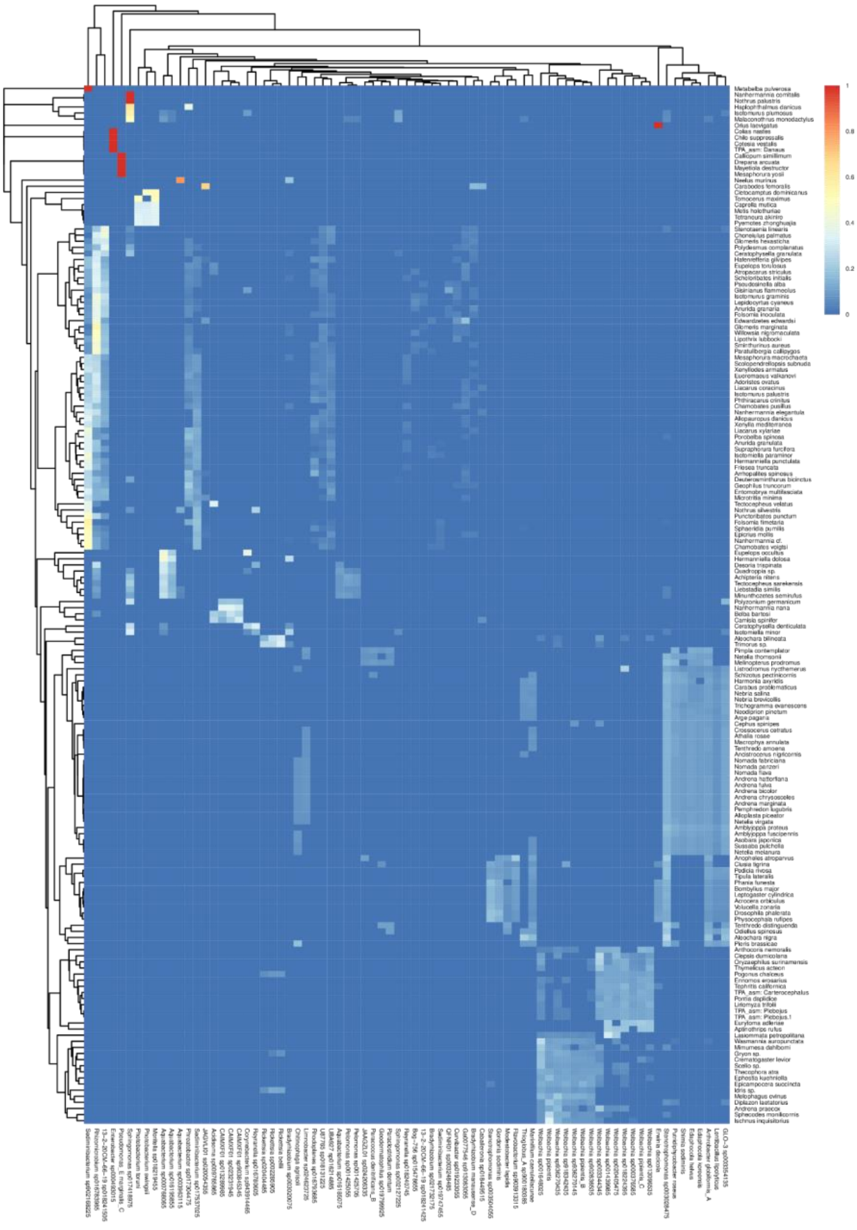

Supplementary Figure 10. Abundance heatmap of microbial-like sequences across NCBI RefSeq arthropods. The columns represent microbial taxa contributing to the reference genomes of arthropods displayed as rows. The color gradient indicates normalized abundance of microbial-like sequences (0-lowest, 1-highest).

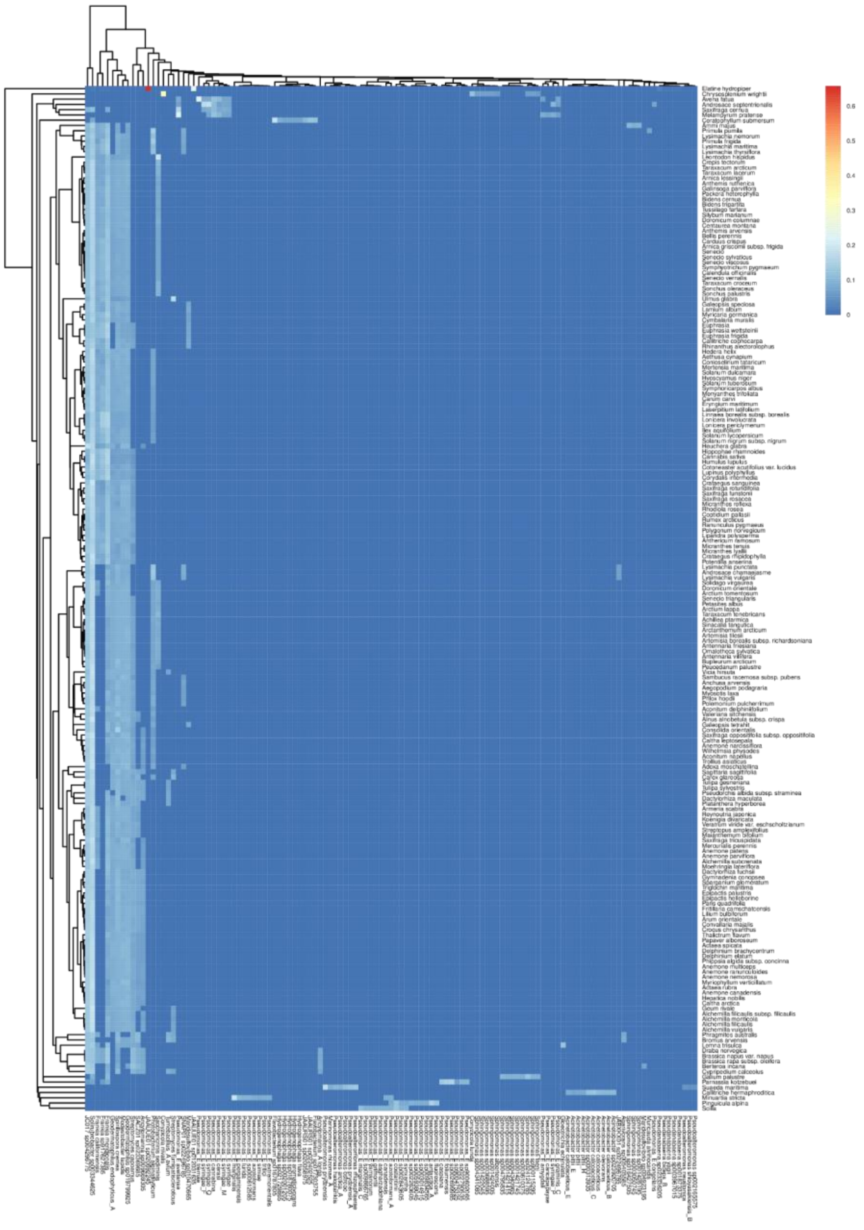

Supplementary Figure 11. Abundance heatmap of microbial-like sequences across PhyloNorway plants. The columns represent microbial taxa contributing to the reference genomes of PhyloNorway plants displayed as rows. The color gradient indicates normalized abundance of microbial-like sequences (0-lowest, 1-highest).

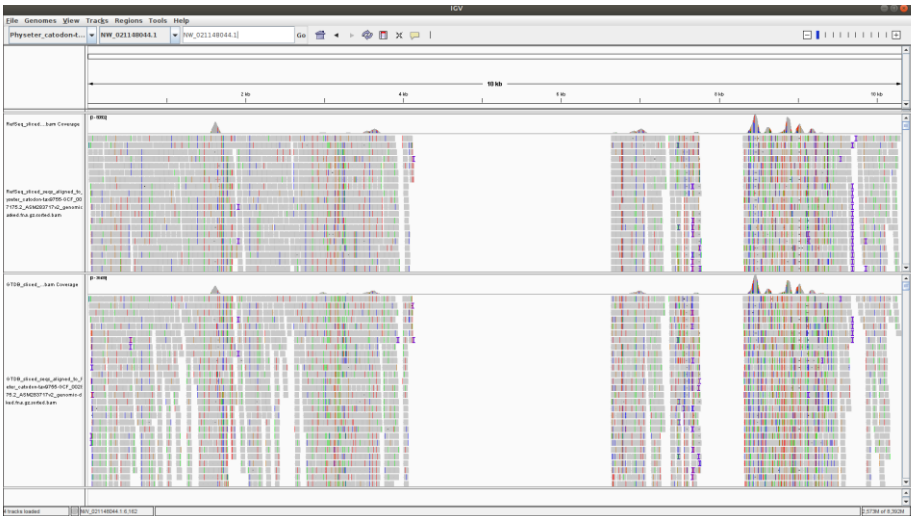

Supplementary Figure 12. Comparison of coverage of a 10 kb region of the sperm whale (*Physeter catodon*, GCA_900411695.1) reference genome by microbial pseudo-reads produced from the microbial RefSeq (top) and microbial GTDB (bottom) databases. The visualization is performed using the Integrative Genome Viewer (IGV).

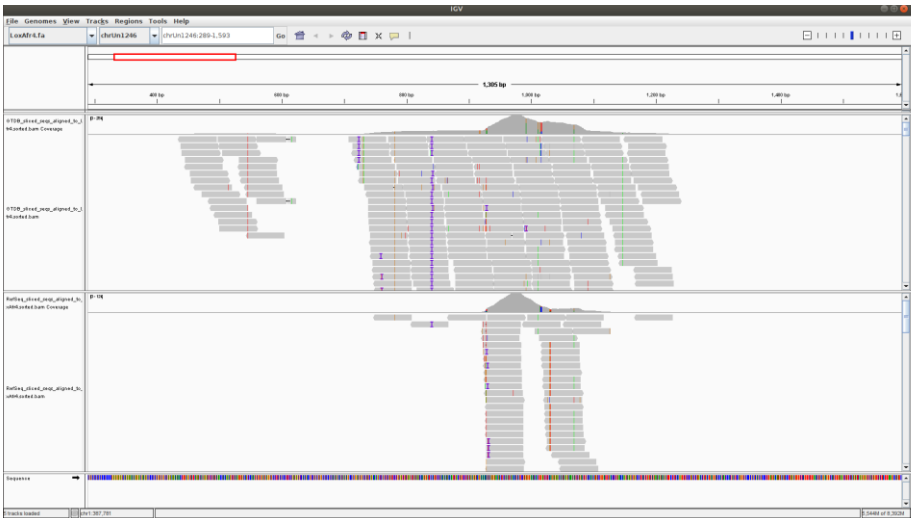

Supplementary Figure 13. Comparison of coverage of a 1.3 kb region of the African bush elephant (*Loxodonta africana*, GCF_000001905.1) reference genome by microbial pseudo-reads produced from the microbial GTDB (top) and microbial RefSeq (bottom) databases. The visualization is performed using the Integrative Genome Viewer (IGV). The visualization demonstrates that microbial GTDB pseudo-reads are capable of discovering more microbial-like regions within the eukaryotic reference genome compared to microbial RefSeq pseudo-reads.

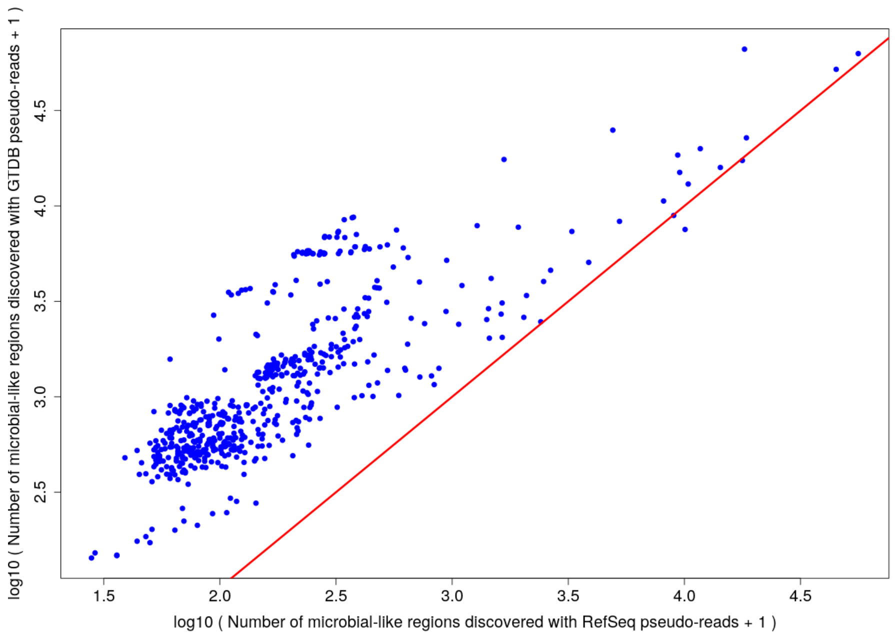

Supplementary Figure 14. Comparison of numbers of microbial-like regions in mammalian reference genomes detected by using microbial GTDB and RefSeq pseudo-reads. One point represents one mammalian reference genome. Red diagonal line highlights equal counts for RefSeq and GTDB.

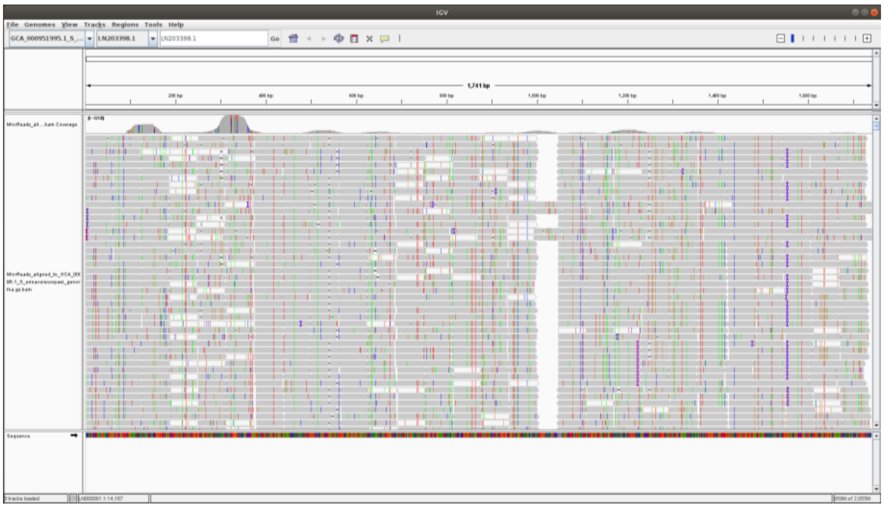

Supplementary Figure 15. Example of coverage of detected exogenous regions by mapped human pseudo-reads to the *Spirometra erinaceieuropaei* (parasitic tapeworm) reference genome GCA_000951995.1, scaffold LN203398.1 that has 100% breadth of coverage by human pseudo-reads. The visualization is performed using the Integrative Genomics Viewer (IGV).
