# Supplementary material for: Improving taxonomic inference from ancient environmental metagenomes by masking microbial-like regions in reference genomes
Source: Gigascience. 2025 Oct 3;14:giaf108. doi: 10.1093/gigascience/giaf108 (PMC12491943; doi:10.1093/gigascience/giaf108)
Supplement: giaf108_GIGA-D-25-00115_Revision_1 [file giaf108_giga-d-25-00115_revision_1.pdf]

GigaScience

Disinfecting eukaryotic reference genomes to improve taxonomic inference from ancient environmental metagenomic data

--Manuscript Draft--

**Manuscript Number:**	GIGA-D-25-00115R1
**Full Title:**	Disinfecting eukaryotic reference genomes to improve taxonomic inference from ancient environmental metagenomic data
**Article Type:**	Technical Note
**Funding Information:**	Knut och Alice Wallenbergs Stiftelse: Dr. Nikolay Oskolkov, Mrs. Chenyu Jin, Mrs. Samantha López Clinton, Dr. Benjamin Guinet, Mrs. Flore Wijnands, Dr. Verena E. Kutschera, Dr. Cormac M. Kinsella, Dr. Peter D. Heintzman, Dr. Tom van der Valk<br>Vetenskapsrådet: Mr. Ernst Johnson
**Abstract:**	Ancient environmental DNA is increasingly essential for reconstructing past ecosystems, particularly when palaeontological and archaeological tissue remains are absent. Detecting ancient plant and animal DNA in environmental samples often relies on using extensive eukaryotic reference genome databases for profiling shotgun metagenomics data. However, microbial contamination in these references can introduce substantial biases in taxonomic assignments, especially given the typical low abundance of plant and animal DNA in such samples. In this study, we present a method for identifying bacterial and archaeal-like sequences in eukaryotic genomes and apply it to nearly 3,000 reference genomes from NCBI RefSeq and GenBank (vertebrates, invertebrates, plants) as well as the 1,323 PhyloNorway plant genome assemblies from herbarium material from northern high-latitude regions. Our analysis reveals microbial-like sequences in many eukaryotic reference genomes, which are most pronounced in the PhyloNorway dataset. We provide a detailed map of the microbial-like regions, including genomic coordinates and taxonomic annotations. This resource enables the masking of microbial-like regions during profiling analyses, thereby improving the reliability of ancient environmental metagenomic datasets for downstream analyses.
**Corresponding Author:**	Nikolay Oskolkov, PhD<br>Lund University: Lunds Universitet<br>Lund, SWEDEN
**Corresponding Author Secondary Information:**	
**Corresponding Author's Institution:**	Lund University: Lunds Universitet
**Corresponding Author's Secondary Institution:**	
**First Author:**	Nikolay Oskolkov, PhD
**First Author Secondary Information:**	
**Order of Authors:**	Nikolay Oskolkov, PhD<br>Chenyu Jin<br>Samantha López Clinton<br>Benjamin Guinet<br>Flore Wijnands

Powered by Editorial Manager® and ProduXion Manager® from Aries Systems Corporation

	Ernst Johnson
	Verena E. Kutschera
	Cormac M. Kinsella
	Peter D. Heintzman
	Tom van der Valk
**Order of Authors Secondary Information:**	
**Response to Reviewers:**	Dear Nikolay Oskolkov,<br><br>Your manuscript "Disinfecting eukaryotic reference genomes to improve taxonomic inference from ancient environmental metagenomic data" (GIGA-D-25-00115) has been assessed by our reviewers. Although it is of interest, we are unable to consider it for publication in its current form. The reviewers have raised a number of points which we believe would improve the manuscript and may allow a revised version to be published in GigaScience. Their reports, together with any other comments, are below. Please also take a moment to check our website at https://www.editorialmanager.com/giga/ for any additional comments that were saved as attachments.<br><br>- I'd like to highlight the suggestion of reviewer #1 "to develop this into a proper workflow (e.g., Snakemake), taking a set of reference fasta files and a set of contaminant fasta files as input, and running through all steps automatically." As also reviewer #2 encountered errors while running the script, implementing the method in easily deployable form will greatly improve uptake and reproducibility. Computational workflows should also be registered in workflowhub.eu and the DOIs cited in the relevant places in the manuscript.<br><br>Authors' comment: Dear Dr. Zauner, we thank you for handling our manuscript and coordinating the review process. We are thankful for the insightful comments from the reviewers, and have now revised our manuscript according to their suggestion. Among the revisions we have improved our workflow, including correcting the workflow running errors encountered by the reviewer #2. We now also provide the conda-environment with the tools used, and rewrote the workflow into a Nextflow pipeline for scalability and reproducibility. Currently, the Nextflow pipeline, as suggested by reviewer #1, accepts a set of reference fasta files and potential exogenous (contaminant) fasta-files as input, and runs through all steps automatically. In addition to having the workflow registered at the SciLifeLab Figshare portal https://doi.org/10.17044/scilifelab.28491956, we have now registered the workflow at Zenodo https://doi.org/10.5281/zenodo.16788411, as well as, as advised by GigaScience, at workflowhub.eu https://doi.org/10.48546/workflowhub.workflow.1846.1. We now provide the corresponding DOI-numbers in the "Data Availability" and "Availability of source code and requirements" sections of the manuscript.<br><br>- As your manuscript presents a method, we will consider it for the "Technical Note" section of the journal. Please have a look at our guidelines for this article type and format the manuscript accordingly (however, we are quite flexible - e.g. you can keep a section titled "discussion", if you feel it's useful.https://academic.oup.com/gigascience/pages/technical_note)<br><br>Authors' comment: Thank you for the suggestion. We have reformatted the manuscript in line with the Technical Note guidelines, and we would like to retain the revised Discussion section, as we believe it provides important context and interpretation of our results.<br><br>- In addition, please register any new software application in the bio.tools and SciCrunch.org databases to receive RRID (Research Resource Identification Initiative ID) and biotoolsID identifiers, and include these in your manuscript. These will facilitate tracking, reproducibility and re-use of your tool.<br><br>Authors' comment: We have now registered the workflow at SciCrunch.org and received the Research Resource Identification Initiative ID (RRID) SCR_027305. We

also registered the workflow at bio.tools and obtained biotools:genex_workflow as the biotools unique resource ID. We included these IDs to the “Data Availability” and “Availability of source code and requirements” sections of the manuscript.

If you are able to fully address these points, we would encourage you to submit a revised manuscript to GigaScience. Once you have made the necessary corrections, please submit online at:

https://www.editorialmanager.com/giga/

If you have forgotten your username or password please use the "Send Login Details" link to get your login information. For security reasons, your password will be reset.

Please include a point-by-point within the 'Response to Reviewers' box in the submission system. Please ensure you describe additional experiments that were carried out and include a detailed rebuttal of any criticisms or requested revisions that you disagreed with. Please also ensure that your revised manuscript conforms to the journal style, which can be found in the Instructions for Authors on the journal homepage. If the data and code has been modified in the revision process please be sure to update the public versions of this too.

The due date for submitting the revised version of your article is 11 Aug 2025.

I look forward to receiving your revised manuscript soon.

Best wishes,
Hans

Dr Hans Zauner
Editor, GigaScience
www.gigasciencejournal.com

Reviewer reports:

Reviewer #1:

Summary
==================

Oskolkov et al present an approach to identify microbial contamination in reference sequence databases. They use this approach to scrutinize two previous publications in the ancient environmental DNA field, and show that some species found in these papers are likely over-estimations due to contamination in the references. They further investigate which microbial references are mostly responsible for the contamination in the examined databases, and show that the PhyloNorway references are highly contaminated.

The problem that Oskolkov et al are tackling here is highly relevant for practitioners, not just in the aeDNA field, but in whole-genome eDNA studies in general. Their findings are worrying to say the least, and indicate that future research and development is needed to fully solve the problem of contamination. This manuscript is also a cautionary tale for data sanitation, and should be read by anyone working with aeDNA.

The methodological approach is straight-forward and sound, and to my understanding makes more sense than the two existing methods that are referred to in the manuscript. Mapping the contaminants against the target (Eukaryotes) seems intuitively more reasonable than the other way round, and allows the authors to produce masks for the genomes, which is interesting and relevant in practice in order to obtain masks.

The paper is very well written and easy to follow. Furthermore, all analyses are thorough, and the figures support the claims that the authors make. All scripts and datasets are made available, and seem well documented.

In summary, the paper is of high quality, and definitely should be published. I do have some suggestions on improvements, which I list below.

Best regards,
Lucas Czech

Authors' comment: we thank the reviewer for the very valuable comments, which have helped us to substantially improve the workflow and the manuscript. Below, we provide as response in bold the reviewer's comments point-by-point.

Major
=================

* One suggestion that we discussed internally after reading the preprint of the manuscript is the following: Using the presented method to map the pseudo-reads against GTDB itself, and/or against the RefSeq organelle or microbial sequences. This would serve as a further validation (everything should show up as "contaminated"), and can be used as a baseline for accuracy and other evaluations.

Authors' response: We thank the reviewer for this suggestion. We have now validated our method by aligning the GTDB pseudo-reads to >820 random GTDB reference sequences and observed the median of 99.1% of breadth of coverage. In addition, aligning the RefSeq pseudo-reads to 25 random RefSeq reference sequences resulted in median breadth of coverage of 97.2%. This confirms the expectation that GTDB and RefSeq reference sequences indeed seem to be close to fully consisting of microbial-like sequences. We have added this validation analysis to the Methods section and Supplementary Material S4 of the manuscript.

* Furthermore, would it make sense to include the human genome as a source of contamination, i.e., create pseudo-reads from it as well? The authors mention that references might be contaminated with human reads, and the workflow as presented should easily be able to accommodate for that, in order to catch that contamination.

Authors' response: Thank you very much for this excellent suggestion. Indeed, the workflow in its current state can easily accommodate the search for potential human contamination. We have pre-computed human pseudo-reads and made them publicly available via the SciLifeLab Figshre, https://doi.org/10.17044/scilifelab.28491956. We have also modified the parameters of the workflow, which now includes the option of using the pre-computed human pseudo-reads if one is interested in detecting "human-like" regions in prokaryotic or eukaryotic reference genomes. As a proof-of-concept we have run the workflow on the Spirometra erinaceieuropaei (parasitic tapeworm) reference genome GCA_000951995.1 which was suspected to contain human contamination in Jensen et al. Nature Communications 2019. We detected that >0.1% of the reference genome includes human-like sequences with over 50 scaffolds with length up to 1.7 kbp having 100% breadth of coverage by human pseudo-reads. The total length of all detected human-like sequences within the parasitic tapeworm reference genome is 1.4 Mbp. We present an IGV visualization of one of the fully covered scaffolds in the newly included Supplementary Figure 15. This analysis was also added to the Methods, Discussion and the new Supplementary Material S5 sections of the manuscript.

* The available scripts are great, however might make it a bit hard to use the approach in practice on other datasets or later versions. To fully leverage the method, and for practitioners to be able to use it on their own data, I suggest to develop this into a proper workflow (e.g., Snakemake), taking a set of reference fasta files and a set of contaminant fasta files as input, and running through all steps automatically. Given that

the scripts already exist, this might be relatively easy to implement, and of great use for the community. I understand though if the authors feel that this is out of scope for this manuscript.

Authors’ response: We agree with the reviewers suggestion. We have now made a major revision of the workflow codes and wrapped them into a Nextflow pipeline, which should ensure the reproducibility and dissemination of the method. Taking into account the previously mentioned implementation of human pseudo-reads, the pipeline follows the suggestion and is general now, i.e. it accepts a reference genome (prokaryotic or eukaryotic) and microbial (GTDB or RefSeq) or human pseudo-reads and outputs the coordinates of predicted exogenous regions.

Minor
=================

* Line 62: "the presence of hippopotamus-like sequence" is syntactically incorrect. Either "the presence of a hippopotamus-like sequence", or "sequences" in plural would work.

Authors’ response: We have now corrected this.

* Line 137, and Supplement S1: Using 60 bp pseudo-reads is yielding very specific hits, which might be what is needed here. Have the authors evaluated smaller pseudo-reads as well? As 30-ish is usually enough to identify a species, I am curious to hear the author's view on this - why 60, and not 30, 40, or 50? Wouldn't those potentially catch more contamination? Or would that be too sensitive and give too many false positives?

Authors’ response: We chose a pseudo-read length of 60 bp to adopt a conservative approach in predicting microbial-like regions. While shorter pseudo-reads (e.g., 30–50 bp) could, in principle, increase the sensitivity and potentially detect more microbial-like sequences, they would also increase the risk of spurious matches due to shorter alignments and reduced sequence complexity, particularly in repetitive genomic regions. Using a length twice the conventional lower limit of ~30 bp prioritizes specificity, ensuring that predicted microbial-like sequences are robust and less likely to represent false positives. This approach minimizes the need for additional downstream validation while maintaining high confidence in our predictions. We acknowledge that this choice may miss some shorter microbial-like regions, but we consider this an acceptable trade-off to improve the reliability of the final masked reference genomes. This reasoning has now been added to the Methods section.

* Line 141, and Supp S1: Allowing for multi-mapping reads is certainly necessary for the reasons the authors have laid out. I am wondering however if 10 is enough here. Have the authors checked if that number is saturated for some genomes? If so, it might need increasing.

Addendum after getting to the supplement: This was indeed evaluated, great! But only on two genomes - might be good enough, but it might differ for other species, or not? I am lacking intuition here, and would like to hear the author's take on this.

Authors’ response: we have indeed evaluated a range of allowed Bowtie2 multi-mappers, which we describe in Supplementary Figure 1. The main conclusion was that the number of discovered microbial-like regions was saturating after 5-10 allowed multi-mappers, and higher thresholds become exponentially computationally more expensive but provide almost no added resolution. We have however now made the number of multi-mapping pseudo-reads allowed by Bowtie2 an explicit parameter of the workflow which could improve the resolution in edge-cases (i.e. regions at extreme copy-number in the reference), please see the last argument here https://github.com/NikolayOskolkov/MCWorkflow.

Powered by Editorial Manager® and ProduXion Manager® from Aries Systems Corporation

* Lines 168-172: While this is the methods section, a very short description of why this PCA was computed as a means of validation, or a foreshadowing link to Fig 4, might help the reader here to understand the reason behind this here already.

Authors' response: We agree this requires additional explanation. We have now added the following sentence motivating the use of PCA for validation of our method:

"Because microbes and plants have distinct k-mer profiles, we used PCA to compare the k-mer composition of microbial-like segments identified by our method with that of endogenous segments, aiming to confirm that these groups indeed form distinct clusters in the PCA plot"

* Lines 220ff: I needed several attempts to read and understand this sentence, and find it confusingly phrased. Might I suggest something along the lines of: "There are 81 primate genomes in the 566 assessed mammalian genomes. Yet, 37 of these are within the top 45 most contaminated genomes." Also, it might be interesting to report the range of contamination of these 37 genomes.

Authors' response: thank you for this excellent suggestion, we have now rephrased this sentence in the main text following the recommendation.

* Lines 295ff: The authors very thoroughly compute the expected percentage of overlap with regions identified as microbial-like in the Hippuris vulgaris reference. How much of that genome was reported as being microbial-like though? Maybe I've missed this, but that might be an interesting fraction to report here as well.

Authors' response: the Hippuris vulgaris reference genome was predicted by our method to contain 57% of microbial-like regions. This was mentioned in the manuscript, however thanks to your suggestion, we have now also emphasized it in the paragraph discussing the by-chance intersection of the mapped reads with the predicted microbial-like regions.

Reviewer #2:

Summary
=================

This manuscript addresses a significant issue in ancient metagenomics: how microbial contamination in reference genomes can influence taxonomic profiling and analytical outcomes. It is a well-written, timely, and highly relevant study. The structure is logical, the arguments are generally sound, and the results are important for the field. However, there are several major limitations that must be addressed to strengthen the study.

I believe this work could be of interest to the readers of GigaScience, but the current analysis and documentation require substantial improvement.

Authors' comment: we thank the reviewer for the very valuable comments, they have helped us to substantially improve the workflow and the manuscript. Below, we address in bold the reviewer's comments point-by-point.

Major Comments
=================

1. Handling of conserved and closely related genes/genomes

It is unclear how the workflow accounts for photosynthetic or ancestrally derived genomic regions in plants, which may be legitimate components of plant genomes. Classifying such regions as microbial contamination may not be appropriate, and masking them in downstream analyses could distort results. For example, GTDB includes over 5,000 cyanobacterial genomes, some of which are among the closest known relatives to plants. Given the potential for genuine sequence similarity, particularly under permissive mapping parameters, the reported contamination may be overestimated. This concern is also relevant to conclusions drawn from single-sample tests.

Authors' response: thank you, we agree this is an important question to discuss further. The GTDB version r214, that was used in this study, contains 3,846 reference genomes (out of the total 394,932 genomes) belonging to the Cyanobacteriota phylum, representing only ~1% of the total number of reference sequences in GTDB. We now tested the enrichment of cyanobacterial pseudo-reads in the predicted microbial-like regions of Hippuris vulgaris and Claytonia eschscholtzii plants from the PhyloNorway dataset, which were predicted to contain the highest percentage of microbial-like regions, 57% and 70%, respectively. Based on the annotation from the GTDB r214, there were 622,336,764 cyanobacterial pseudo-reads out of total 26,089,195,106 pseudo-reads, i.e. 2.4%, pre-computed from the GTDB reference sequences. However, we observed only 2,739,015 cyanobacterial pseudo-reads of of total 481,618,468 (i.e. 0.6%) aligned to the Hippuris vulgaris, and only 1,688,444 cyanobacterial pseudo-reads out of 408,824,087 (i.e. 0.4%) aligned to Claytonia eschscholtzii, reference genomes from the PhyloNorway dataset. This implies that cyanobacterial pseudo-reads do not align more often to the two PhyloNorway plant references than would be expected by chance. Therefore, although potential over-masking of plant references following our method is plausible, it is not supported by our analysis of GTDB data. Nevertheless, because of ambiguity of the analysis, we cannot fully reject the hypothesis that due to the genuine sequence similarity between cyanobacterial and plant genomes, the fractions of microbial-like regions in some plant reference genomes predicted in this study may be overestimated. However, if such over-masking occurs and these regions represent genuine host genome sequences, microbial sequences in aeDNA samples can still align to them, potentially leading to erroneous taxonomic assignments. In this context, masking these regions remains beneficial, as it promotes a more conservative approach and thus a more reliable detection of true species present in ancient samples.

We have now added these results to the Discussion section of the revised manuscript.

2. Contamination in GTDB Database

The manuscript does not address possible contamination within the GTDB database itself, which appears to be used uncritically for cleaning eukaryotic reference genomes. Prior work has raised concerns about microbial genome contamination (e.g., https://pubmed.ncbi.nlm.nih.gov/38809778/). At a minimum, this limitation should be discussed; ideally, it should be assessed computationally. For instance, pseudo-reads generated from a well-annotated genome (e.g., human) could be mapped against microbial references to demonstrate the complexity and potential pitfalls in cross-superkingdom contamination detection.

Alternatively, I encourage the authors to develop or include a method to evaluate the specificity and sensitivity of the contaminated regions identified in the eukaryotic genomes. This would reduce reliance on external assumptions and provide stronger validation for their conclusions.

Authors' response: thank you, we agree this is an important consideration. We indeed assume GTDB to represent a microbial "ground-truth" and free from eukaryotic contamination as it is a highly curated database. We aimed to further validate this assumption by the additional analysis that we performed by aligning human pseudo-reads (prepared in the same way as the microbial pseudo-reads described in the manuscript) to >820 random GTDB and 25 RefSeq microbial reference sequences. In

Powered by Editorial Manager® and ProduXion Manager® from Aries Systems Corporation

the whole experiment we observed only 3 human reads aligned to any microbial reference which represents a negligible breadth of coverage with median 0% and ensures that the microbial pseudo-reads used in this manuscript are very likely free from any substantial eukaryotic contamination.

In contrast, when we screen eukaryotic reference genomes for the presence of human-like sequences, we discover substantially higher coverage. For example, Spirometra erinaceieuropaei (parasitic tapeworm) reference genome GCA_000951995.1, indicated over 8 million aligned human pseudo-reads covering >0.1% of the reference genome with the total length of human-like sequences of 1.4 Mbp. We present an IGV visualization of one of 100% covered scaffolds of the parasitic tapeworm reference in the newly included Supplementary Figure 15. In addition, screening the reference genome GCF_002220235.1 of Bathycoccus prasinos (green algae), we detected over 236,000 aligned human pseudo-reads covering ~0.2% of the reference with the total length of the potentially exogenous regions of 37 kbp.

To further address the sensitivity and specificity of our method, we ran it using a random subset of 6.5 million GTDB pseudo-reads and the screened reference genome represented concatenated hg38 human reference genome with 16 microbial reference genomes (corresponding to 726 reference sequences on chromosome and scaffold level) used in Pochon at al. Genome Biology 2023. We observed only 1 microbial pseudo-read mapped to one chromosome (chr 12) out of 24 canonical and 432 decoy chromosomes in the human hg38 reference genome, while 202,762 pseudo-reads were mapped to the microbial reference sequences. This demonstrates the high specificity of our method as well as the very low risk of Bowtie2 non-specific alignments (please see the next reviewer's comment regarding the risk of including non-specific alignments).

We have now added this analysis to the Methods, Discussion as well as Supplementary Material S5 sections of the revised manuscript.

3. Quantitative Analysis of Sequence Similarity

The manuscript lacks a detailed analysis of the similarity between microbial reads and the eukaryotic regions they map to. Given that the Bowtie2 setting --very-sensitive allows alignments with substantial mismatches (e.g., 60 bp reads with >10 bp differences), there is a risk of including non-specific alignments. A quantitative analysis of sequence identity across these alignments would be helpful, along with a justified similarity threshold to exclude highly divergent mappings that may not be of microbial origin.

Authors' response: thank you for this very valid point. Bowtie2 has a special non-trivial scoring system to determine whether a read will be mapped or not. The scoring system is not solely based on the exact number of mismatches but includes multiple other metrics such as base quality, gaps, clipping etc. Nevertheless, Bowtie2 prioritizes high-scoring (i.e. more similar) alignments, and heavily penalizes divergence. Empirically, we can see that a 60 bp read with more than ~6 mismatches (average nucleotide identity ANI=90%) will usually fail to align, even under --very-sensitive –end-to-end. For example, tested on RefSeq plants and PhyloNorway references with aligned GTDB pseudo-reads, we observe on average 4 ± 0.4 and 4 ± 0.2 mismatches per read, respectively, i.e. the similarity of ANI=93%. The comment that "Bowtie2 setting --very-sensitive allows alignments with substantial mismatches (e.g., 60 bp reads with >10 bp differences)" is not directly supported by our empirical testing. Taking into account that a typical ANI threshold for ancient metagenomics projects is set much lower than 93%, i.e. often down to 85% (Pochon et al. 2023, Huebler et al. 2019), due to DNA damage, we assume the risk of non-specific alignments with the Bowtie2 mapping parameters used in this study is low.

Also (please see the answer to the previous question), in the experiment when we aligned human pseudo-reads to microbial references and vice-versa microbial pseudo-reads to concatenated human + microbial references we discovered extremely few (3 and 1 respectively) miss-mapped reads, which highlights the high specificity of Bowtie2

Powered by Editorial Manager® and ProduXion Manager® from Aries Systems Corporation

alignments with –very-sensitive and –end-to-end arguments used in this study.

We have now added this additional analysis of sequence similarity threshold to the Supplementary Material S1 section.

4. Reproducibility and Workflow Usability

The computational workflow should be included in the GitHub repository to ensure reproducibility. I attempted to run the small test example provided, but encountered multiple issues on both a Unix-based laptop and a Linux server. These included absolute paths in scripts, unresolved dependencies, and failures in execution. I recommend that the authors:

- Fix hardcoded paths and ensure portability across environments
- Provide a working bash script that runs successfully on clean systems
- Include a conda environment file or, at a minimum, document tool versions and dependencies

Here is a sample of the errors encountered:

```
xxxxxx@xxxxxxxxx MCWorkflow % ./micr_cont_detect.sh GCF_002220235.fna.gz data GTDB 4 GTDB_sliced_seqs_sliding_window.fna.gz GTDB_fna2name.txt

PREPARING FILES FOR ANALYSIS OF GCF_002220235.fna.gz REFERENCE GENOME
mkdir: GCF_002220235.fna.gz_GTDB: File exists

BUILDING BOWTIE2 INDEX FOR GCF_002220235.fna.gz REFERENCE GENOME
ALIGNING MICROBIAL READS WITH BOWTIE2 TO GCF_002220235.fna.gz REFERENCE GENOME
(ERR): "data/GCF_002220235.fna.gz" does not exist or is not a Bowtie 2 index
Exiting now ...
[main_samview] fail to read the header from "-".
[W::hts_set_opt] Cannot change block size for this format
samtools sort: failed to read header from "-"
samtools index: "MicrReads_aligned_to_GCF_002220235.fna.gz.bam" is in a format that cannot be usefully indexed
RANKING GCF_002220235.fna.gz CONTIGS BY NUMBER OF MAPPED MICROBIAL READS
[main_samview] fail to read the header from "MicrReads_aligned_to_GCF_002220235.fna.gz.bam".
sed: 1: "contigs_abund_sorted_GT ...": command c expects \ followed by text
sed: 1: "contigs_abund_sorted_GT ...": command c expects \ followed by text
COMPUTING BREADTH OF COVERAGE FOR EACH CONTIG AND COORDINATES OF MICROBIAL CONTAMINATION FOR GCF_002220235.fna.gz REFERENCE GENOME

AGGREGATING RESULTS FOR GCF_002220235.fna.gz REFERENCE GENOME AND CLEANING
paste: total_length_per_ref.txt: No such file or directory
sed: 1: "contigs_boc_sorted_GTDB ...": command c expects \ followed by text
sed: 1: "contigs_boc_sorted_GTDB ...": command c expects \ followed by text
rm: total_length_per_ref.txt: No such file or directory
rm: boc_per_ref.txt: No such file or directory
COMPUTING LIST OF MOST ABUNDANT MICROBES CONTMAMINATING GCF_002220235.fna.gz REFERENCE GENOME
[main_samview] fail to read the header from "MicrReads_aligned_to_GCF_002220235.fna.gz.bam".

ANALYSIS FOR GCF_002220235.fna.gz REFERENCE GENOME FINISHED
```

Powered by Editorial Manager® and ProduXion Manager® from Aries Systems Corporation

	SUCCESSFULLY<br>xxxxxx@xxxxxxxxx MCWorkflow % ll data<br>total 103232<br>-rwxr-xr-x 1 xxxxxx staff 4941250 May 9 11:35 GCF_002220235.fna.gz<br>-rw-r--r-- 1 xxxxxx staff 13403961 May 9 11:50 GCF_002220235.fna.gz.1.bt2l<br>-rw-r--r-- 1 xxxxxx staff 7519068 May 9 11:50 GCF_002220235.fna.gz.2.bt2l<br>-rw-r--r-- 1 xxxxxx staff 709 May 9 11:50 GCF_002220235.fna.gz.3.bt2l<br>-rw-r--r-- 1 xxxxxx staff 3759531 May 9 11:50 GCF_002220235.fna.gz.4.bt2l<br>-rw-r--r-- 1 xxxxxx staff 13403961 May 9 11:50 GCF_002220235.fna.gz.rev.1.bt2l<br>-rw-r--r-- 1 xxxxxx staff 7519068 May 9 11:50 GCF_002220235.fna.gz.rev.2.bt2l<br>drwxr-xr-x 6 xxxxxx staff 192 May 9 11:50 GCF_002220235.fna.gz_GTDB<br>-rw-r--r-- 1 xxxxxx staff 67376 May 9 11:50 bowtie2-build.log<br>```<br><br>Authors' response: thank you very much for testing our workflow and your suggestions! We agree and we have now substantially improved the reproducibility and usability of the workflow. Specifically, first, we resolved the hard-coded paths issue which now makes it possible to run the workflow from the cloned repository without manually editing the paths. Second, we added the conda environment file, that assists installing the workflow tools, and corresponding clarifications to the README in the github repository page. Finally, we wrapped the workflow up via the Nextflow framework for further scalability and reproducibility. The workflow has been tested on one laptop, one stationary workstation and two computer clusters where it ran without errors. We invite the reviewer to test the workflow again following the command lines presented here:<br><br>https://github.com/NikolayOskolkov/MCWorkflow?tab=readme-ov-file#quick-start.
**Additional Information:**	
**Question**	**Response**
Are you submitting this manuscript to a special series or article collection?	No
**Experimental design and statistics**<br><br>Full details of the experimental design and statistical methods used should be given in the Methods section, as detailed in our Minimum Standards Reporting Checklist. Information essential to interpreting the data presented should be made available in the figure legends.<br><br>Have you included all the information requested in your manuscript?	Yes
**Resources**<br><br>A description of all resources used, including antibodies, cell lines, animals and software tools, with enough information to allow them to be uniquely identified, should be included in the Methods section. Authors are strongly encouraged to cite Research Resource	Yes

Identifiers (RRIDs) for antibodies, model organisms and tools, where possible.<br><br>Have you included the information requested as detailed in our Minimum Standards Reporting Checklist?	
**Availability of data and materials**<br><br>All datasets and code on which the conclusions of the paper rely must be either included in your submission or deposited in publicly available repositories (where available and ethically appropriate), referencing such data using a unique identifier in the references and in the "Availability of Data and Materials" section of your manuscript.<br><br>Have you have met the above requirement as detailed in our Minimum Standards Reporting Checklist?	Yes
GigaScience has policies and guidelines in place for the use of generative AI-writing tools such as ChatGPT. If you have used such writing tools to assist with writing the manuscript this must be declared and cited in the text. Authors should not list AI-writing tools and other AI-assisted technologies as an author or co-author and should acknowledge that they are fully responsible for text generated or refined by AI-writing tools.<p><br><br>A summary of use (particularly in the introduction or among methods) needs to be included at the end of the paper, and the outputs should also be included as a supplementary file hosted in GigaDB or other open repositories. Please read our guidelines for more information. <p><br><br>By submitting to GigaScience, you are aware of the journal's AI-writing tools policy, and if you have declared use of such tools below, you have acknowledged this where appropriate in your manuscript and have made a summary of use and outputs available. <p>	No

<b>AI-assisted writing tools have been used in the preparation of this manuscript?	

Powered by Editorial Manager® and ProduXion Manager® from Aries Systems Corporation

Manuscript with tracked changes

Click here to access/download;Manuscript;Reference_Contamination_manu

Improving Taxonomic Inference from Ancient Environmental Metagenomes by Masking Microbial-like Regions in Reference Genomes

Disinfecting eukaryotic reference genomes to improve taxonomic inference from ancient environmental metagenomic data

Authors: Nikolay Oskolkov[1+], Chenyu Jin[2,3,4], Samantha López Clinton[2,3,4], Benjamin Guinet[2,3], Flore Wijnands[2,5], Ernst Johnson[2,5], Verena E. Kutschera[6], Cormac M. Kinsella[3,7], Peter D. Heintzman[2,5], and Tom van der Valk[2,3,8]

+ to whom correspondence should be addressed

1. Department of Biology, National Bioinformatics Infrastructure Sweden, Science for Life Laboratory, Lund University, Lund, Sweden
2. Centre for Palaeogenetics, Svante Arrhenius väg 20C, 10691 Stockholm, Sweden.
3. Department of Bioinformatics and Genetics, Swedish Museum of Natural History, Stockholm, Sweden.
4. Department of Zoology, Stockholm University, Stockholm, Sweden
5. Department of Geological Sciences, Stockholm University, Stockholm, Sweden
6. Department of Biochemistry and Biophysics, National Bioinformatics Infrastructure Sweden, Science for Life Laboratory, Stockholm University, Solna, Sweden
7. Department of Cell and Molecular Biology, National Bioinformatics Infrastructure Sweden, Science for Life Laboratory, Uppsala University, Uppsala, Sweden
8. Scilifelab, Stockholm, Sweden

Keywords: environmental DNA, ancient metagenomics, microbial-like regions

~~**contamination**~~

Abstract

Ancient environmental DNA is increasingly vital~~essential~~ for reconstructing past ecosystems, particularly when palaeontological and archaeological tissue remains are absent. Detecting ancient plant and animal DNA in environmental samples ~~often~~ relies on using extensive eukaryotic reference genome databases for profiling ~~shotgun~~ metagenomics data. However, many eukaryotic genomes contain regions with high sequence similarity to microbial DNA, which can lead to the misclassification of bacterial and archaeal reads as eukaryotic. This issue is especially problematic in ancient eDNA datasets, where ~~microbial contamination in these references can introduce substantial biases in taxonomic assignments, especially given the typical low abundance of~~ plant and animal DNA is typically present at very low abundance..~~in such samples.~~ In this study, we present a method for identifying bacterial- and archaeal-like sequences in eukaryotic genomes and apply it to nearly 3,000 reference genomes from NCBI RefSeq and GenBank (vertebrates, invertebrates, plants) as well as the 1,323 PhyloNorway plant genome assemblies from herbarium material from northern high-latitude regions. We find that~~Our analysis reveals~~ microbial-like regions are widespread across eukaryotic genomes and ~~sequences in many eukaryotic reference genomes, which are most pronounced in the PhyloNorway dataset. We~~ provide a comprehensive resource of their~~detailed map of the microbial-like regions, including~~ genomic coordinates and taxonomic annotations. This resource enables the masking of microbial-like regions during profiling analyses, thereby improving the reliability of ancient environmental metagenomic datasets for downstream analyses.

Introduction

Ancient environmental DNA (aeDNA) is a tool for studying past ecosystems, especially in contexts where traditional archaeological and palaeontological tissue remains, such as bones and seeds, are absent [1-4]. It consists of genetic traces left by organisms in the environment, such as soil, sediments or ice~~soils, sediments, ice, or other environmental samples~~, and allows for the reconstruction of past biodiversity and ecological communities to provide insight into species extinction, vegetation changes, and ecosystem responses to climatic shifts and anthropogenic impacts.

The often limited amount of DNA that can be isolated from ancient environmental samples imposes ~~significant~~ constraints on analytical methods. Coupled with the often low relative abundance of plant and animal DNA preserved in most environments, as compared to microbes, aeDNA analysis primarily relies on a reference-based approach for taxonomic profiling, which assumes similarity between the aeDNA query and the reference genome sequences. Therefore, robust aeDNA-derived community reconstructions are dependent on the accuracy of read identification by comparison to genomic reference databases. Consequently, ~~both~~ the quality of both the aeDNA data and the reference databases is crucial for reliable inferences. Microbial-like sequences in~~within~~ reference genomic databases, originating either ~~that are either derived~~ from non-endogenous sources (contamination) or from evolutionary similarity to microbial genomes (e.g., due to ancient horizontal gene transfer or the endosymbiotic origins of plastids~~similar to highly-diverged taxa (evolutionarily conserved or convergent~~), can be a potential source of false-positive taxonomic identifications. In such cases, microbial sequences present in aeDNA data may be mistakenly classified as belonging to a eukaryotic reference genome due to sequence similarity.

The existence of contaminant-like sequences is a pervasive issue in reference genomes with multiple examples reported in the literature [5-7]. For instance, contaminated reference sequences, such as the presence of a hippopotamus-like sequence in the alpaca

mitochondrial reference genome [8] and human sequences in parasitic worm genomes [9], have led to inaccurate inferences of evolutionary relationships [10], divergence times [8], and horizontal gene transfer events [11]. The inclusion of such eukaryotic reference genome contamination, most commonly originating from microbial or human sources, can occur at any stage throughout the genome assembly process [12].

Several analytical approaches have been proposed to address the issue of microbial contamination in reference genomes. For instance, Lu and Salzberg [13] suggested a computational method for masking erroneous sequences from draft genomes of eukaryotic pathogens. This is implemented by splitting the draft pathogenic references into pseudo-reads and filtering them using *k*-mer based Kraken classification [14, 15] and Bowtie2 alignment [16] against the human genome and National Center for Biotechnological Information Reference Sequence Database (NCBI RefSeq) microbial references. Conterminator is another program for contamination detection in the NCBI GenBank, RefSeq, and non-redundant (NR) reference databases proposed by Steinegger and Salzberg [17]. The program operates by an exhaustive all-against-all sequence comparison across kingdoms by splitting reference sequences into short segments, extracting their *k*-mers, grouping the *k*-mers, and then performing cross-kingdom alignments of the representative sequences in order to predict the contaminating sequences. This approach identified over 2,000,000 contaminated entries in the GenBank database [18]. Furthermore, the Physeter [19] and ~~-~~CheckM [20] tools have also been used to estimate contamination levels in NCBI RefSeq bacterial genomes. Lastly, ongoing efforts by NCBI, such as introducing the FCS-GX tool [21], which uses hashed *k*-mer matches and a curated reference database, in addition to more traditional VecScreen [22] and BLAST [23], are retroactively reducing the prevalence of contaminant-like sequences within the NCBI RefSeq and GenBank databases.

However, these efforts do not address ~~the same problems in~~ alternative databases, such as those comprising genome-wide data, e.g. PhyloNorway, PhyloAlps [24], or ~~in~~ legacy versions of the NCBI RefSeq database [25, 26], that are commonly used in workflows for large-scale

metagenomics analysis (e.g. Kraken [14, 15]). Therefore, there is a need for a generic tool that identifies and removes these problematic ~~contaminant-like~~ sequences, particularly those similar to bacteria and archaea, from any genomic datasets that will be used as reference sequences for ancient environmental metagenomics analysis. In addition, although the microbial NCBI RefSeq is one of the largest available reference databases that has previously been used for estimating the amount of contamination in eukaryotic reference genomes [13, 17], the advent of the more diverse and comprehensive microbial Genome Taxonomy DataBase (GTDB) [27], allows for greater sensitivity in identifying regions in genome assemblies that are characterised by containing microbial-like sequences.

The aim of this study was therefore threefold. First, we developed a workflow ~~generic algorithm~~ applicable to any eukaryotic reference genome in FASTA format, which outputs exact genomic coordinates of microbial-like sequences in BED-format. The coordinate file can then be used to mask the eukaryotic reference genome~~genomes~~ for various applications, including taxonomic profiling from ancient metagenomics data. Second, we sought higher identification accuracy of ~~for regions of~~ microbial-like regions~~sequences~~ by using the curated and non-redundant microbial GTDB database, ~~–~~the most comprehensive of its type at present~~– with the goal of minimising false-positive discoveries in ancient environmental metagenomics studies~~. Lastly, to allow for future investigation of the sources and mechanisms of contamination, we annotated and summarized each genomic region identified as microbial-like by the relative contribution of each microbial taxon.

To demonstrate~~showcase~~ our approach, we aligned all microbial sequences from the GTDB database to six panels of eukaryotic reference databases and identified the genomic regions that are similar to bacterial and archaeal sequences. We show that up to 70% of a taxon's reference genome assembly can have shared similarity with bacteria and archaea (microbial-like). After masking microbial-like regions from the reference genomes, we re-analysed two empirical ancient metagenomic datasets and showed that some eukaryotic species detections

can be ~~entirely~~ driven by alignments of reads to the microbial-like regions~~sequences~~. We anticipate that masking reference genomes for these microbial-like sequences will greatly reduce reference-genome-derived false-positive taxonomic assignments in ancient and modern environmental metagenomic studies.

Methods

We selected 4,294 reference genomes of varying degrees of completeness and from a broad spectrum of taxonomic groups. This included (1) chromosome-level reference genome assemblies for 96 plants, (2) 114 invertebrates, and (3) 162 non-mammalian vertebrate species available from NCBI RefSeq, release 213; (4) 566 chromosome- and scaffold-level mammalian genome assemblies from NCBI GenBank, release 254 (if a species had multiple assemblies, we selected the one with highest N50 value); (5) all 2,033 chromosome- and scaffold-level arthropod reference genomes available in NCBI GenBank, release 256; and (6) 1,323 genome-skimmed contig-level plant assemblies from the PhyloNorway project (DataverseNO, V1) [28]. We individually constructed Bowtie2 [16] indices for all 4,294 reference genomes in the six genome groups.

Next, we fragmented all microbial (bacterial + archaea) reference genomes present in the GTDB dataset ([27]; release 214~~ from the 28th of April 2023~~) into 60 bp long segments using a sliding window with a 10 bp step. The fragmentation length of 60 bp was chosen to ensure sufficient specificity when matching microbial sequences to eukaryotic references, as it is twice the commonly accepted ~30 bp minimum threshold for organism-level specificity across the tree of life [14, 15] and matches the average fragment length often obtained from aeDNA datasets. This resulted in a collection of $2.6*10^{10}$ ~~nucleotide~~ sequences representing microbial sequencing data (reads), which we refer to as “pseudo-reads” in this study. In addition, microbial RefSeq and human hg38 reference genome pseudo-reads were prepared in a

similar manner, and we provide these together with the workflow (see also Data and Code Availability). Their use is discussed in Supplementary Material S4 and S5. These reads were aligned to each indexed eukaryotic reference genome using Bowtie2 on the –very-sensitive and –end-to-end settings and allowing ~~, with~~ up to 10 multi-mappers to be retained per read. The retention of multi-mappers ensured that multi-copy microbial-like regions from the same microbe within a reference were also detected. In our testing,~~ we discovered that~~ keeping multi-mappers greatly improved the detection sensitivity for microbial-like regions in the eukaryotic reference genomes, with this gain saturating after retaining approximately 10 multi-mapped positions (Supplementary Figure 1). We considered genomic regions covered by at least one microbial pseudo-read as microbial-like. We visually validated a set of the microbial-like regions using the Integrative Genomics Viewer (IGV) [29], and confirmed their coverage by microbial pseudo-reads (Supplementary Figure 2). For additional details about the alignment procedure, see Supplementary Material S1.

Next, we~~We then~~ used *samtools depth* [30] and *bedtools merge* [31] to detect and extract the coordinates of regions in the eukaryotic reference genomes that were covered by microbial pseudo-reads in BED format (Table 1, which also includes data on the abundance of the most prevalent microbes in each identified genomic region). The breadth of coverage of microbial-like sequences was computed as the fraction of reference genome nucleotides covered at least once by microbial pseudo-reads. We validated the successful preparation of GTDB pseudo-reads by aligning them to over 820 randomly selected GTDB reference sequences and observing a median breadth of coverage of 99.1%. This result supports our expectation that the GTDB reference sequences themselves should look to be almost entirely composed of microbial-like sequences. We used *samtools* [30] and custom bash and R scripts for annotating the reference genomes with the most abundant source microbial species. The annotation was done both genome-wide and for each individual microbial-like region in the BED file. In the latter case, only the top 5 most abundant microbial taxa per region were recorded. The entire workflow is schematically presented in Figure 1 (see also Data and Code

Availability).

Due to the nature of the PhyloNorway dataset, being the only included dataset with genome-skim assemblies from museum specimens and the potential secondary microbial growth, it ~~The PhyloNorway dataset~~ exhibited the highest proportions of microbial-like sequences of the genome groups. To validate our method, we therefore extracted the microbial-like (presumed exogenous) and remaining (presumed endogenous) segments from the PhyloNorway reference genomes with *bedtools getfasta* and *bedtools complement* [31] using their coordinates in the BED file. Next, we applied the Mash algorithm [32] (*mash dist* function was used) to construct a matrix of pairwise distances based on their *k*-mer composition among all species, separating the endogenous and exogenous segments. We then computed a Principal Component~~component~~ Analysis (PCA) on the obtained matrix using the *scikit-learn* module in Python. Because microbes and plants have distinct k-mer profiles, we used PCA to compare the k-mer composition of microbial-like segments identified by our method with that of endogenous segments, thereby aiming to confirm that these groups indeed form distinct clusters.

Finally, our ~~The~~ workflow was verified against two empirical aeDNA datasets, which capture the flora and fauna from ~~either~~ across the Arctic and~~or~~ the Kap Kobenhavn Formation in Greenland respectively [28, 33]. We used one sample from each study, i.e. cr9_67 from [28] (further referred to as the “Arctic sample”) and 69_B2_100_L0_KapK-12-1-35 [33] (further referred to as the “Greenland sample”). Adapter-removed reads from these samples were aligned with Bowtie2 [16] to the PhyloNorway reference genome assemblies, together with the Asian Elephant as a proxy for woolly mammoths (EleMax1, GCF_024166365.1) and Human (GRCH38, GCF_000001405.40) reference genomes. These two latter mammalian references were added as decoys to attract mammalian reads via competitive mapping, since mammals were also reported in these samples in the original studies [28, 33]. Next, we used~~We next applied~~ *bedtools closest* [31] to compute the number of intersections of the

aligned reads with the microbial-like sequences detected by our workflow in the PhyloNorway reference genomes. A custom R script was used to compute the null distribution of such intersections corresponding to random placement of the reads within the reference genomes.

Results

After applying our workflow to a diverse set of eukaryotic reference genomes, we ranked the results by the percentage of the genome flagged as microbial-like sequence separately for each genome group (Figures 2 and 3, and Supplementary Tables 1-6).

The non-mammalian vertebrate reference genomes exhibit the lowest overall levels of microbial-like sequence, comprising~~i.e.~~ <0.2% of the reference, as compared to other genome groups, with~~where~~ the Tiger barb fish (*Puntigrus tetrazona*; NCBI id: GCF_018831695.1) having~~has~~ the greatest amount (0.16%). In contrast, mammals, plants, and invertebrate genomes often contained moderate degrees of microbial-like sequence~~, i.e.~~ up to ~~~1.5~~2%, where Tibetan antelope (*Pantholops hodgsonii*; GCF_000400835.1; 1.4% microbial-like sequence), rice (*Oryza sativa*; GCF_001433935.1; 1.6%), and fruit-fly (*Drosophila ananassae*; GCA_017639315.2; 2.3%) contain the most microbial-like sequence in each respective group.

During the course of this study, NCBI RefSeq flagged the version of the Tibetan antelope reference genome used here (GCF_000400835.1) as containing a high level~~magnitude~~ of contamination and replaced it with an improved version (GCA_040182635.1). Using our workflow, we found that this reduced the percentage of microbial-like inserts in the Tibetan antelope genome from 1.4% to 0.12%, thereby indirectly validating the accuracy of our approach. Although the improved version of the Tibetan antelope reference genome contains an order of magnitude less microbial-like sequences, we suggest that the remaining microbial-like sequences detected here are likely due to the broader scope of the GTDB microbial

dataset we used for the detections. We also found that the only The two reference genomes available for extinct organisms, Steller's sea cow (*Hydrodamalis gigas*; GCA_013391785.1; 1.3%) and thylacine (*Thylacinus cynocephalus*; GCA_007646695.3; 0.7%), are were found to be among the top five mammalian genomes with the most microbial-like sequences sequence according to our method (Figure 3, Supplementary Table 1). This highlights the challenging aspect of high-quality genome assembly from historical samples, as We consider this plausible, as these genomes are derived from degraded samples havewith preservation conditions amenable to microbial contamination [34].

Among the mammalian genomes with the most microbial-like sequencessequence, there is a significant over-representation of primates. There are 81 primate genomes in the 566 , consisting of 37 out of the top 45 mammalian genomes we assessed, (i.e. 14% of the total. Yet, 37 of these contain 0.4-0.6% of microbial-like regions and are within the top 45 genomes, 82%), while there are only 81 primate genomes out of the total 566 mammalian genomes assessed (i.e. 82%14%) (Fisher exact test, p=2.6*10^{-11}), ranked by the fraction of microbial-like sequences (Supplementary Table 1). Similarly. Second, bovids, including cattle (*Bos taurus*; GCA_947034695.1; 0.3%), wild yak (*Bos mutus*; GCA_027580195.1; 0.3%), and American bison (*Bison bison*; GCF_000754665.1; 0.2%), whichthat are common organisms of interest in aeDNA studies, were are placed among the top mammalian organisms with the most microbial-like inserts with up to 9 Mb of their genomes deemed microbial-likeconsisting of microbial-like sequence.

Among plant reference genomes, rice (*Oryza sativa*; GCF_001433935.1; 1.6% microbial-like sequences), rapeseed (*Brassica napus*; GCF_020379485.1; 1.4%), corn (*Zea mays*; GCF_902167145.1; 1%) and pumpkin (*Cucurbita pepo*; GCF_002806865.1; 0.7%) have the highest fractions, i.e. 0.7-1.6%, of microbial-like inserts, corresponding to genomic lengths of 2-6 Mb (Figure 3, Supplementary Table 2). During Interestingly, during the period of this

study, the rice (*Oryza sativa*; GCF_001433935.1) reference genome, which we found to have the highest levels of microbial-like sequences, was suppressed by NCBI as a result of standard genome annotation processing, further serving ~~which can serve~~ as an independent~~additional~~ validation of our microbial-like detection workflow. Invertebrates demonstrate similar levels, i.e. 0.5-2%, corresponding to genomic lengths of 1-5 Mb, with several *Drosophila* references~~the *Drosophila* genus~~ among the invertebrates with most potentially contaminated reference genomes (Figure 3 and Supplementary Table 4).

GenBank arthropod reference genomes, which mostly comprise scaffold-level assemblies, on average demonstrate a comparable degree of microbial-like sequences to ~~as in~~ NCBI RefSeq vertebrates and invertebrates (Figure 2). Although with some outliers, such as ~~However, the most extreme examples show higher levels than those showcased from NCBI RefSeq vertebrates, invertebrates, and plants (Figure 3). For instance,~~ the water flea (*Daphnia dubia*; GCA_013387435.1), with ~~has~~ ~7% of microbial-like sequences ~~(which corresponds to~~ 7 Mb of genomic length), and~~followed by~~ the Labrador sulphur butterfly (*Colias nastes*; GCA_907164665.1; 4%; 20 Mb) (Figure 3 and Supplementary Table 5).

The PhyloNorway dataset, an extensive ~~a~~ collection of high-latitude skimmed plant genomes assembled from herbarium voucher specimens that is currently necessary for ancient environmental metagenomics ~~widely used in environmental ancient DNA~~ studies [24, 28, 33], demonstrated particularly high levels of microbial-like sequences ~~compared to all other datasets we analyzed in this work~~ (Figure 2). For instance, the PhyloNorway genomes with the highest proportions of microbial-like sequences, such as grassleaf spring beauty flower (*Claytonia eschscholtzii*; 70%), common mare's-tail ~~plant~~ (*Hippuris vulgaris*; 57%), and herbaceous seepweed (*Suaeda maritima*; 31%), were well above the levels observed in other datasets (Figure 3 and Supplementary Table 6). To further assess~~validate~~ the difference in nucleotide composition between endogenous eukaryotic and microbial-like sequences in the

PhyloNorway dataset, we performed a PCA based on pairwise k-mer matching distances and visualized the two leading principal components (Figure 4)~~visualized the two leading principal components of a PCA computed on their pairwise distances in Figure 4~~. We observed distinct clustering of the microbial-like and endogenous regions, supporting the inference that the identified microbial-like sequences are ~~not~~ derived from non-plant sources~~plant genomes~~. In addition, reference sequences of *Hippuris vulgaris* projected on a~~the~~ hierarchical dendrogram based~~built~~ on pairwise *k*-mer distances between NCBI RefSeq plants and bacteria, demonstrated that microbial-like sequences cluster together with bacterial genomes whereas~~and~~ endogenous sequences cluster with plant genomes (Supplementary Figure 3).

The aquatic plant genus *Hippuris* was previously reported as one of the most abundant taxa in ancient sediments from northern Siberia (Arctic sample) [28] and Greenland (Greenland sample) [33]. These identifications were based on alignments to the PhyloNorway reference genome assemblies, in which *H. vulgaris* was the sole representative of the genus. Our analysis revealed that *H. vulgaris* contains one of the highest proportions of microbial-like sequences among the surveyed species. We therefore assessed the extent to which the findings in [28] and [33] may have been influenced by the presence of microbial-like sequences in the *H. vulgaris* reference genome.

~~The aquatic plant genus *Hippuris* was found to be one of the most abundant in the two empirical studies examined and was reported from both northern Siberia (Arctic sample) [28] and Greenland (Greenland sample) [33]. Since this finding was based on alignments against the PhyloNorway reference genome assemblies, where *Hippuris vulgaris* was the only representative of *Hippuris* genus, and *Hippuris vulgaris* was shown by our analysis to be one of the species with the most extreme fractions of microbial-like sequences, we evaluated to what extent the conclusions of [28] and [33] could be affected by the presence of microbial-like sequences in the reference genome. The PhyloNorway reference genome assembly of *Hippuris vulgaris* consists of 433,631 contigs, which have a bimodal breath of coverage distribution for the microbial-like sequence fraction in our analysis, with modes at~~

~~approximately 0 and 100% (Supplementary Figure 4). This indicates that a substantial proportion of *Hippuris vulgaris* contigs appear to be free from microbial-like sequences (the zero mode). In the Arctic sample however, a clear unimodal distribution of microbial-like fractions from the 20,213 *Hippuris vulgaris* contigs with at least one read mapped demonstrates that the vast majority of these contigs had close to 100% breath of coverage of microbial-like sequences (Supplementary Figure 5A). This implies that the *Hippuris*-identified reads from the Arctic sample have a much higher affinity to the microbial-like *Hippuris vulgaris* contigs, suggesting these reads originated from a microbial source. This indicates a potential mechanism for the discovery of *Hippuris* in [28]. In contrast, the reads attributed to *Hippuris vulgaris* in the Greenland sample from [33] mapped to 73,911 contigs that included both "endogenous" (to a larger extent) and "microbial-like" (to a lesser extent) contigs (Supplementary Figure 5B). Nevertheless, the peak at 100% of microbial-like fraction is not negligible, implying that the number of endogenous DNA sequences of *Hippuris vulgaris* in the Greenland sample was likely overestimated.~~

The PhyloNorway reference genome assembly of *H. vulgaris* consists of 433,631 contigs, which have a bimodal breadth of coverage distribution for the microbial-like sequence fraction in our analysis, with modes at approximately 0 and 100% (Supplementary Figure 4). This indicates that a substantial proportion of *H. vulgaris* contigs appear to be free from microbial-like sequences (the zero mode). In the Arctic sample however, a unimodal distribution of microbial-like fractions from the 20,213 *H. vulgaris* contigs with at least one read mapped demonstrates that the vast majority of these contigs had close to 100% breadth of coverage of microbial-like sequences (Supplementary Figure 5A). This implies that the *Hippuris*-identified reads from the Arctic sample have a much higher affinity to the microbial-like *Hippuris vulgaris* contigs, suggesting these reads originated from a microbial source. In contrast, the reads attributed to *H. vulgaris* in the Greenland sample [33] mapped to 73,911 contigs that included both "endogenous" (to a larger extent) and "microbial-like" (to a lesser extent) contigs (Supplementary Figure 5B). Nevertheless, the peak at 100% of microbial-like

fraction is not negligible, implying that the number of endogenous DNA sequences of *H. vulgaris* in the Greenland sample was likely overestimated.

Of the 119,854 reads mapped in the Arctic sample, 116,483 (i.e. 97%) intersected with regions identified as microbial-like in the *Hippuris vulgaris* reference. To check whether this represents a statistically significant enrichment, we performed 300 random assignments of the 119,854 reads to the *Hippuris vulgaris* reference within the length limits of each contig, and demonstrated that approximately 58.8 ± 0.3 % would be a by-chance expectation if the intersection of mapped reads with the regions of microbial contamination was purely random. The observed 97% intersection is far beyond ($p < 0.0033$) the expected percentage (Supplementary Figure 6A). For the Greenland sample, where *Hippuris* was reported to be one of the most abundant genera in [33], 1,014,237 reads out of 1,367,627 reads, or 74%, mapped to microbial-like regions of the *Hippuris vulgaris* reference, which was again significantly higher ($p < 0.0033$) than the null expectation (Supplementary Figure 6B). For more details about *Hippuris vulgaris* follow up, see Supplementary Material S2. Therefore, for both the Arctic and Greenland samples, we conclude that the majority of their reads assigned to *Hippuris vulgaris* are of likely microbial origin.

Of the 119,854 reads mapped to the *H. vulgaris* reference for the Arctic sample, 116,483 (i.e. 97%) intersected with regions identified as microbial-like.As our method predicted that the *H. vulgaris* reference comprises 57% microbial-like sequences, we investigated whether the 97% intersect represents a statistically significant enrichment by performing 300 random assignments of the 119,854 reads to the *H. vulgaris* reference within the length limits of each contig. This showed that approximately 58.8 ± 0.3 % would be a by-chance expectation if the intersection of mapped reads with the microbial-like regions was random. The observed 97% intersection is beyond the expected percentage ($p<0.0033$) (Supplementary Figure 6A). For the Greenland sample, where *Hippuris* was reported to be one of the most abundant genera [33], 1,014,237 reads out of 1,367,627 reads, or 74%, mapped to microbial-like regions of the *H. vulgaris* reference, which was also significantly higher than the null expectation ($p<0.0033$)

(Supplementary Figure 6B). Therefore, for both the Arctic and Greenland samples, the majority of their reads assigned to *H. vulgaris* are of likely microbial origin. For more details about the *H. vulgaris* analyses, see Supplementary Material S2.

We next sought to explore the potential mechanisms for~~of~~ the origins of microbial-like sequences in mammalian reference genomes. To achieve this, we quantified the abundance of the most common microbe matches ~~microbes~~ in each eukaryotic reference genome and compared the reference genomes based on the patterns of microbial genus/species presence observed. The most common microbe matches ~~microbes~~ across the mammalian reference genomes with the highest levels of microbial-like sequences form several clusters (Figure 5). First, the highly abundant *Streptococcus* sp000187445 bacterium is shared across six equid reference genomes (*Equus quagga burchellii*, GCA_026770645.1; *Equus przewalskii*, GCF_000696695.1; *Equus caballus*, GCF_002863925.1; *Equus asinus*, GCF_016077325.2; *Equus quagga*, GCF_021613505.1; *Equus asinus asinus*, GCA_003033725.1) and the white rhinoceros (*Ceratotherium simum simum*, GCA_023653735.1). Since these seven reference genomes were submitted to NCBI by different sequencing centres~~centers~~, lab contamination as a source for the microbial-like sequences is unlikely. The co-occurence of *Streptococcus* sp000187445 in equids and rhinos is intriguing, as these taxa all comprise part of the odd-toed ungulates, order Perissodactyla ~~(order Perissodactyla)~~. The remaining perissodactyl in the dataset, South American tapir (*Tapirus terrestris*), had the next highest abundance of *Streptococcus* sp000187445 but falls~~fell~~ outside of the perissodactyl cluster. This suggests that either *Streptococcus* sp000187445 could be a probiotic microbe endogenous to the perissodactyl microbiome and is erroneously integrated into the genome assemblies, or ~~or that~~ part of the ancestral perissodactyl genome was evolutionarily convergent with *Streptococcus* sp000187445. Second, the D16-34 sp910588485 bacterium (belonging to genus *Adlercreutzia*) is highly abundant and shared by Snow sheep (*Ovis nivicola lydekkeri*, GCA_903231385.1) and Scimitar oryx (*Oryx dammah*, GCF_014754425.2) reference genomes, both produced by different centres. These~~centers. The~~ two mammalian species

belonging~~belong~~ to the Bovidae family again suggest~~which suggests~~ some plausible similarity in their microbiomes or alternatively evolutionary convergence. Analogously, reference genomes~~,~~ produced by different centres, of four mammalian species belonging to family Canidae, i.e. maned wolf (*Chrysocyon brachyurus*, GCA_028533335.1), arctic fox (*Vulpes lagopus*, GCF_018345385.1), dingo (*Canis lupus dingo*, GCF_003254725.2), and domestic dog (*Canis lupus familiaris*, GCF_013276365.1), all ~~–~~share highly abundant microbial-like sequences from *Paracoccus denitrificans B*, which is a soil-associated bacterium not previously shown to be related to the canid microbiome. Therefore, evolutionary convergence could be an ~~can be a plausible~~ explanation for co-occurence of *Paracoccus denitrificans B*-like sequences in the reference genomes of Canidae mammals. In addition, at least two more large clusters including broad groups of both mammalian and microbial organisms can be distinguished: 1) an ungulate cluster driven by intermediately abundant *Aureimonas A endophytica*, *Aliidongia dinghuensis*, *Mycobacterium malmesburyense*, *Anaerotardibacter muris*, *Muriiphilus lacisalsi*, and 2) a non-human primates cluster driven by moderately abundant *Streptomyces griseoincarnatus*, *Streptomyces kurssanovii*, *Chromatium weissei*, *Zobellia laminariae*, *Caproicibacter* sp900184925, *Streptomyces* sp020873915 and *Paeniglutamicibacter antarcticus*. These ~~The latter~~ two clusters suggest that microbial-like sequences from multiple microbes contributed to reference genomes of evolutionarily related organisms possibly due to shared ecological environments and hence similarities of their microbiomes or evolutionary convergence. In contrast, there are a few clusters which likely point at some commonalities that are not strongly host-associated. For example, *Tumebacillus A avium* is shared at high abundance between Sunda flying lemur (*Galeopterus variegatus*, GCA_004027255.2) and Asian black bear (*Ursus thibetanus thibetanus*, GCA_009660055.1), which are not closely related species and the reference genomes were produced by different research institutes. Figure 5 also demonstrates that many microbial species, such as *Spirillospora cremea*, *Azonexus* sp016617495, D16-34 sp910588485, *Anaerotardibacter muris*, *Chromatium weissei* and *Aliidongia dinghuensis*, are moderately abundant across a wide range of distinct mammals. Since these microbes are also typical inhabitants of soil and

aquatic environments, we hypothesize that they either represent environmental or shared lab-reagent contamination which was incorporated during the sampling, sequencing and genome assembly process, or can also be due to evolutionary convergence. For further discussion of microbial-like sequences composition within NCBI RefSeq / GenBank plants, invertebrates, non-mammalian vertebrates, arthropods, and PhyloNorway plants, please see Supplementary Material S3 and Supplementary Figures 7-11.

Discussion

Microbial-like sequences present in reference genome databases represent an ongoing~~a growing~~ problem [35]. While human contamination was recognized some time ago to be one of the major challenges in ancient microbial genomics [9, 36], the opposite scenario of microbial contamination in animal and plant reference genomes became particularly evident in the rapidly developing ancient environmental DNA field [1-4], where reference-based organism discovery is commonplace~~widely used~~. Microbial contamination can occur at different steps of reference database generation [19, 20] and subsequently poses a ~~serious~~ risk of false-positive discovery, which, if unaccounted for~~neglected~~, can lead to erroneous results and interpretations in downstream analyses. Previous attempts to address this ~~issue~~ [12, 13, 17, 19, 20] have concentrated on flagging contaminated eukaryotic references without ~~providing~~ more comprehensive and quantitative information about specific locations and origins of microbial-like regions. Here, we aimed to mitigate biases introduced by ~~we aim at mitigating~~ microbial-like sequences with higher~~more~~ precision and mechanistic understanding, ~~while specifically~~ concentrating on reference genomes that are particularly important in the field~~for the area~~ of ancient environmental DNA.

We present a workflow for detecting microbial-like sequences within eukaryotic reference genomes, and a collection of BED files (see example in Table 1) with coordinates of microbial-

like sequences from a large custom dataset of mammalian, non-mammalian vertebrate, invertebrate, arthropod, and plant reference genomes (N= ~4,300). The application of this workflow allows researchers within the aeDNA field to mask the portions of the genome that match microbial-like sequences. Therefore, rather than merely marking entire reference genomes as unsuitable, our approach seeks to retrieve specific contigs and regions annotated with potentially underlying microbial taxa. The method also enables more precise microbial-like detections by utilizing the largest available microbial genome database (GTDB [27]), which includes both archaeal and bacterial reference genomes.

Although our approach follows a similar strategy put forward by Lu and Salzberg [13] and Steinegger and Salzberg [17], there are a few conceptual and technical differences. Lu and Salzberg [13] implemented splitting of eukaryotic reference genomes into pseudo-reads, screening them with Kraken [14, 15], and aligning them with Bowtie2 [16] against human and microbial references, while Steinegger and Salzberg [17] applied cross-kingdom *k*-mer matching across the NCBI RefSeq, GenBank, and NR databases. In contrast, we follow the opposite approach of splitting microbial (bacterial, archaeal) reference genomes into pseudo-reads and aligning them against eukaryotic references, resulting in precise coordinates of microbial-like regions within eukaryotic reference genomes. The conceptual difference is that only eukaryotic pathogens were used in [13], while we utilise all NCBI RefSeq plant and animal references and the PhyloNorway dataset of skimmed plant genome assemblies. Therefore, our method is not specific to the NCBI databases but applicable to any custom nucleotide sequence in FASTA format. Another conceptual difference is that both [13] and [17] used the microbial NCBI RefSeq database, which has limited size and diversity compared to the non-redundant GTDB database [27] used in our testing (see Supplementary Material S4), which increased detection sensitivity to microbial-like sequences in eukaryotic reference genomes.

As microbial databases like NCBI RefSeq and GTDB are continually updated with new assemblies, masking of eukaryotic reference genomes with BED files from this study should

not be considered an exhaustive solution. There will be a need to update the microbial-like regions presented here as microbial databases continue to grow.

We present a novel method for detecting microbial presence within eukaryotic reference databases, and a collection of BED files (see example in Table 1) with coordinates of microbial-like sequences from a large custom dataset of ~4,300 mammalian, non-mammalian vertebrate, invertebrate, arthropod, and plant reference genomes. The application of this method will allow researchers within the aeDNA field to mask portions of the genome with a potentially microbial origin. Therefore, rather than merely marking reference genomes as contaminated, our approach seeks to retrieve specific contigs and regions annotated with underlying microbial taxa. The method also enables more precise detections by utilizing the largest available microbial genome database (GTDB [27]), which includes both archaeal and bacterial genomes.

Although our approach follows a similar strategy suggested by Lu and Salzberg [13] and Steinegger and Salzberg [17], there are a few conceptual and technical differences. Lu and Salzberg [13] implemented splitting of eukaryotic reference genomes into pseudo-reads, screening them with Kraken [14, 15] and aligning them with Bowtie2 [16] against human and microbial references, while Steinegger and Salzberg [17] applied cross-kingdom *k*-mer matching across the NCBI RefSeq, GenBank, and NR databases. In contrast, we follow the opposite approach of splitting microbial (bacterial, archaeal) reference genomes into pseudo-reads and aligning them against eukaryotic references, which results in precise coordinates of microbial-like regions within eukaryotic reference genomes. The conceptual difference is that only eukaryotic pathogens were used in [13], while we utilise all NCBI RefSeq plant and animal references and the PhyloNorway dataset of plant genome assemblies. Therefore, our method is not specific to the NCBI databases but applicable to any custom nucleotide sequence in FASTA format. Another conceptual difference is that both [13] and [17] used the microbial NCBI RefSeq database, which has limited size and diversity compared to the non-redundant GTDB database [27], which we used in our testing (see Supplementary Material

~~S4) and increases detection sensitivity to microbial-like sequences in eukaryotic reference genomes.~~

The importance of microbial database coverage can be seen from the study of Kjaer et al. [33], who used a previous version of GTDB (release 95) as a decoy, in order to ensure that animal and plant hits were not originating from microbial reads. Nevertheless, we report in this study that a substantial amount of sequences attributed to the plant findings in the original work [33] are microbial-like. It is likely that a proportion of microbial-like reads remained in [33] after filtering the data with the GTDB release 95, and further microbial-like sequence discovery became possible with the substantially larger GTDB release 214 database used here.

~~As microbial databases such as NCBI RefSeq and GTDB are continually updated with new assemblies, the masking of eukaryotic reference genomes with BED files from this study should not be considered an exhaustive solution. There will be a need to update the microbial-like regions presented here as microbial databases continue to grow.~~

A potential limitation of our proposed method is the assumption that the GTDB database used in this study represents a microbial "ground truth" and is free from eukaryotic contamination, as prior work raised concerns about the quality of GTDB [41]. However, when we aligned human pseudo-reads (prepared using the same procedure as the microbial pseudo-reads in this study) to over 820 randomly selected GTDB and 25 RefSeq microbial reference genomes, only three human reads aligned to any microbial reference, corresponding to a negligible breadth of coverage with median 0%, thereby supporting the assumption that it is highly unlikely that the microbial pseudo-reads used in our study contain eukaryotic contamination. In contrast, screening eukaryotic reference genomes revealed substantially higher levels of human-like sequences. For example, the *Spirometra erinaceieuropaei* (parasitic tapeworm) reference genome (GCA_000951995.1) contained over 8 million aligned human pseudo-reads, covering more than 0.1% of the genome with a total of 1.4 Mb of human-like sequences (Supplementary Figure 15). Similarly, analysis of the *Bathycoccus prasinos* (green algae)

reference genome (GCF_002220235.1) revealed over 236,000 aligned human pseudo-reads, covering approximately 0.2% of the genome and spanning a total of 37 kb of human-like regions. This confirms the significantly higher similarity of the eukaryotic pseudo-reads to eukaryotic references compared to bacterial references, which in turn supports the assumption of negligible eukaryotic contamination within GTDB.

To further evaluate the sensitivity and specificity of our workflow, we applied it to a random subset of 6.5 million GTDB pseudo-reads. The screened reference genome consisted of the concatenated hg38 human reference genome and 16 microbial reference genomes (corresponding to 726 reference sequences at the chromosome and scaffold levels) as used in [43]. We observed that only a single microbial pseudo-read aligned to one human chromosome (chr 12) out of the 24 canonical and 432 decoy chromosomes in the hg38 reference genome, whereas 202,762 pseudo-reads aligned to the microbial reference sequences. These results highlight the high specificity of the method and indicate a low likelihood of non-specific alignments by Bowtie2.

There is currently no ultimate bioinformatic solution for distinguishing microbial contamination from true taxonomic hits in aeDNA studies. Here, we emphasize that we can only classify certain regions of eukaryotic reference genomes as “microbial-like”, as there is no guarantee they are of microbial origin and could instead be due to sequence conservation or convergence, or from potential *in vivo* insertion of microbial sequences into eukaryotic genomes.

The importance of microbial database coverage can be seen from the study of Kjaer et al. [33], who used an older version of GTDB (release 95) as a decoy, in order to ensure that animal and plant hits were not originating from microbial reads. Nevertheless, we report in this study that a substantial amount of sequences attributed to the plant findings in the original work [33] are microbial-like. It is likely that a proportion of microbial-like reads were still remaining in [33] after filtering the data with the GTDB release 95, and further microbial-like

~~sequence discovery became possible with the larger GTDB release 214 database used here.~~

This challenge is particularly evident when identifying microbial-like sequences in plant reference genomes, such as those in the PhyloNorway dataset. As previously demonstrated [42], the evolutionary relationship between certain bacteria and plant organelles (e.g., chloroplasts) often results in genuine sequence similarities. This overlap can lead to ambiguous classifications and misannotations within databases such as NCBI GenBank. Because the GTDB database includes a subset of cyanobacterial genomes, some of which are among the closest known relatives to plants, we aimed to assess the potential for overestimating microbial-like regions in the PhyloNorway plant reference genomes by our method. First, GTDB version r214, used in our study, contains 3,846 reference genomes from the phylum Cyanobacteriota, approximately 1% of the total 394,932 reference genomes. Second, we specifically evaluated whether cyanobacterial pseudo-reads were overrepresented in the predicted microbial-like regions of two plant species from the PhyloNorway dataset, *Hippuris vulgaris* and *Claytonia eschscholtzii*, which had the highest predicted proportions of microbial-like regions (57% and 70%, respectively). According to GTDB r214 annotations, cyanobacterial pseudo-reads accounted for 622,336,764 out of 26,089,195,106 total pseudo-reads (2.4%). However, only 2,739,015 cyanobacterial pseudo-reads (0.6%) aligned to *Hippuris vulgaris* and 1,688,444 (0.4%) to *Claytonia eschscholtzii*, out of 481,618,468 and 408,824,087 total aligned pseudo-reads, respectively. These results suggest that the cyanobacterial pseudo-reads do not align to the plant reference genomes more frequently than expected by chance. Therefore, while our analysis does not indicate that over-masking plant references due to sequence similarity is a major concern in our workflow, the inherent ambiguity means some microbial-like regions in plant genomes may still be overestimated. However, if such over-masking occurs and these regions represent genuine host genome sequences, microbial sequences in aeDNA samples can still align to them and potentially lead to erroneous taxonomic assignments. In this context, masking these regions remains beneficial, as it promotes a more conservative approach and thus a more reliable

detection of true species present in aeDNA data.

~~There is currently no ultimate bioinformatic solution for distinguishing microbial contamination from true taxonomic hits in aeDNA studies. Here, we emphasize that we can only classify certain regions of eukaryotic reference genomes as “microbial-like”, which does not guarantee they are truly of microbial origin but could rather be due to sequence conservation or convergence, or from the potential insertion of microbial sequences into eukaryotic genomes.~~

Our study highlights the need to avoid using sequencing data mapped to publicly available genomes, without also accounting for microbial-like regions within the reference genome assemblies. When working with only a handful of reference genomes, it is possible to evaluate the contamination of each individually, either through bioinformatic methods or by consulting the methods used to construct each assembly. However, this quickly becomes unfeasible~~infeasible~~ in metagenomic studies, where data is often mapped against hundreds or thousands of different reference genomes. Mapping sequence reads against potentially contaminated reference genomes can lead to spurious detections of animal and plant organisms. We therefore suggest, as a preventive measure, to either mask the microbial-like regions in the reference genomes before performing mapping, or add~~adding~~ a validation step after mapping to confirm that the detection signal does not derive from the microbial-like regions.

References

[1] Slon V, Hopfe C, Weiß CL, Mafessoni F, de la Rasilla M, Lalueza-Fox C, Rosas A, Soressi M, Knul MV, Miller R, Stewart JR, Derevianko AP, Jacobs Z, Li B, Roberts RG, Shunkov MV, de Lumley H, Perrenoud C, Gušić I, Kućan Ž, Rudan P, Aximu-Petri A, Essel E, Nagel S,

Nickel B, Schmidt A, Prüfer K, Kelso J, Burbano HA, Pääbo S, Meyer M. Neandertal and Denisovan DNA from Pleistocene sediments. Science. 2017 May 12;356(6338):605-608.

[2] Zavala EI, Jacobs Z, Vernot B, Shunkov MV, Kozlikin MB, Derevianko AP, Essel E, de Fillipo C, Nagel S, Richter J, Romagné F, Schmidt A, Li B, O'Gorman K, Slon V, Kelso J, Pääbo S, Roberts RG, Meyer M. Pleistocene sediment DNA reveals hominin and faunal turnovers at Denisova Cave. Nature. 2021 Jul;595(7867):399-403.

[3] Vernot B, Zavala EI, Gómez-Olivencia A, Jacobs Z, Slon V, Mafessoni F, Romagné F, Pearson A, Petr M, Sala N, Pablos A, Aranburu A, de Castro JMB, Carbonell E, Li B, Krajcarz MT, Krivoshapkin AI, Kolobova KA, Kozlikin MB, Shunkov MV, Derevianko AP, Viola B, Grote S, Essel E, Herráez DL, Nagel S, Nickel B, Richter J, Schmidt A, Peter B, Kelso J, Roberts RG, Arsuaga JL, Meyer M. Unearthing Neanderthal population history using nuclear and mitochondrial DNA from cave sediments. Science. 2021 May 7;372(6542):eabf1667.

[4] Pedersen MW, De Sanctis B, Saremi NF, Sikora M, Puckett EE, Gu Z, Moon KL, Kapp JD, Vinner L, Vardanyan Z, Ardelean CF, Arroyo-Cabrales J, Cahill JA, Heintzman PD, Zazula G, MacPhee RDE, Shapiro B, Durbin R, Willerslev E. Environmental genomics of Late Pleistocene black bears and giant short-faced bears. Curr Biol. 2021, Jun 21;31(12):2728-2736.e8. doi: 10.1016/j.cub.2021.04.027. Epub 2021 Apr 19.

[5] Longo MS, O'Neill MJ, O'Neill RJ. Abundant human DNA contamination identified in non-primate genome databases. PLoS One. 2011 Feb 16;6(2):e16410.

[6] Gruber K. Here, there, and everywhere: From PCRs to next-generation sequencing technologies and sequence databases, DNA contaminants creep in from the most unlikely places. EMBO Rep. 2015 Aug;16(8):898-901.

[7] Merchant S, Wood DE, Salzberg SL. Unexpected cross-species contamination in genome

sequencing projects. PeerJ. 2014 Nov 20;2:e675. doi: 10.7717/peerj.675.

[8] Peter D. Heintzman, Grant D. Zazula, James A. Cahill, Alberto V. Reyes, Ross D.E. MacPhee, Beth Shapiro, Genomic Data from Extinct North American *Camelops* Revise Camel Evolutionary History, *Molecular Biology and Evolution*, Volume 32, Issue 9, September 2015, Pages 2433–2440.

[9] Jensen, T.Z.T., Niemann, J., Iversen, K.H. *et al.* A 5700 year-old human genome and oral microbiome from chewed birch pitch. *Nat Commun* 10, 5520 (2019).

[10] Laurin-Lemay S, Brinkmann H, Philippe H. Origin of land plants revisited in the light of sequence contamination and missing data. Curr Biol. 2012 Aug 7;22(15):R593-4. doi: 10.1016/j.cub.2012.06.013. PMID: 22877776.

[11] G. Koutsovoulos, S. Kumar, D.R. Laetsch, L. Stevens, J. Daub, C. Conlon, H. Maroon, F. Thomas, A.A. Aboobaker, M. Blaxter, No evidence for extensive horizontal gene transfer in the genome of the tardigrade Hypsibius dujardini, Proc. Natl. Acad. Sci. U.S.A. 113 (18) 5053-5058, https://doi.org/10.1073/pnas.1600338113 (2016).

[12] Cornet, L., Baurain, D. Contamination detection in genomic data: more is not enough. *Genome Biol* 23, 60 (2022). https://doi.org/10.1186/s13059-022-02619-9

[13] Lu J, Salzberg SL (2018) Removing contaminants from databases of draft genomes. PLoS Comput Biol 14(6): e1006277. https://doi.org/10.1371/journal.pcbi.1006277

[14] Wood DE, Salzberg SL. Kraken: ultrafast metagenomic sequence classification using exact alignments. Genome Biol. 2014 Mar 3;15(3):R46. doi: 10.1186/gb-2014-15-3-r46.

[15] Wood DE, Lu J, Langmead B. Improved metagenomic analysis with Kraken 2. Genome Biol. 2019 Nov 28;20(1):257. doi: 10.1186/s13059-019-1891-0.

[16] Langmead B, Salzberg SL. Fast gapped-read alignment with Bowtie 2. Nat Methods. 2012 Mar 4;9(4):357-9. doi: 10.1038/nmeth.1923.

[17] Steinegger M, Salzberg SL. Terminating contamination: large-scale search identifies more than 2,000,000 contaminated entries in GenBank. Genome Biol. 2020 May 12;21(1):115. doi: 10.1186/s13059-020-02023-1. PMID: 32398145; PMCID: PMC7218494.

[18] Sayers EW, Beck J, Bolton EE, Brister JR, Chan J, Comeau DC, Connor R, DiCuccio M, Farrell CM, Feldgarden M, Fine AM, Funk K, Hatcher E, Hoeppner M, Kane M, Kannan S, Katz KS, Kelly C, Klimke W, Kim S, Kimchi A, Landrum M, Lathrop S, Lu Z, Malheiro A, Marchler-Bauer A, Murphy TD, Phan L, Prasad AB, Pujar S, Sawyer A, Schmieder E, Schneider VA, Schoch CL, Sharma S, Thibaud-Nissen F, Trawick BW, Venkatapathi T, Wang J, Pruitt KD, Sherry ST. Database resources of the National Center for Biotechnology Information. Nucleic Acids Res. 2024 Jan 5;52(D1):D33-D43. doi: 10.1093/nar/gkad1044.

[19] Lupo V, Van Vlierberghe M, Vanderschuren H, Kerff F, Baurain D, Cornet L. Contamination in Reference Sequence Databases: Time for Divide-and-Rule Tactics. Front Microbiol. 2021 Oct 22;12:755101. doi: 10.3389/fmicb.2021.755101.

[20] Parks, D. H., Imelfort, M., Skennerton, C. T., Hugenholtz, P., and Tyson, G. W. (2015). CheckM: assessing the quality of microbial genomes recovered from isolates, single cells, and metagenomes. *Genome Res.* 25, 1043–1055. doi: 10.1101/gr.186072.114

[21] Astashyn A, Tvedte ES, Sweeney D, Sapojnikov V, Bouk N, Joukov V, Mozes E, Strope PK, Sylla PM, Wagner L, Bidwell SL, Brown LC, Clark K, Davis EW, Smith-White B, Hlavina

W, Pruitt KD, Schneider VA, Murphy TD. Rapid and sensitive detection of genome contamination at scale with FCS-GX. Genome Biol. 2024 Feb 26;25(1):60. doi: 10.1186/s13059-024-03198-7. PMID: 38409096; PMCID: PMC10898089.

[22] Schäffer AA, Nawrocki EP, Choi Y, Kitts PA, Karsch-Mizrachi I, McVeigh R. VecScreen_plus_taxonomy: imposing a tax(onomy) increase on vector contamination screening. Bioinformatics. 2018 Mar 1;34(5):755-759. doi: 10.1093/bioinformatics/btx669.

[23] Camacho C, Coulouris G, Avagyan V, Ma N, Papadopoulos J, Bealer K, Madden TL. BLAST+: architecture and applications. BMC Bioinformatics. 2009 Dec 15;10:421.

[24] Alsos IG, Lavergne S, Merkel MKF, Boleda M, Lammers Y, Alberti A, Pouchon C, Denoeud F, Pitelkova I, Pușcaș M, Roquet C, Hurdu BI, Thuiller W, Zimmermann NE, Hollingsworth PM, Coissac E. The Treasure Vault Can be Opened: Large-Scale Genome Skimming Works Well Using Herbarium and Silica Gel Dried Material. Plants (Basel). 2020 Apr 1;9(4):432. doi: 10.3390/plants9040432. PMID: 32244605; PMCID: PMC7238428.

[25] O'Leary NA, Wright MW, Brister JR, Ciufo S, Haddad D, McVeigh R, Rajput B, Robbertse B, Smith-White B, Ako-Adjei D, Astashyn A, Badretdin A, Bao Y, Blinkova O, Brover V, Chetvernin V, Choi J, Cox E, Ermolaeva O, Farrell CM, Goldfarb T, Gupta T, Haft D, Hatcher E, Hlavina W, Joardar VS, Kodali VK, Li W, Maglott D, Masterson P, McGarvey KM, Murphy MR, O'Neill K, Pujar S, Rangwala SH, Rausch D, Riddick LD, Schoch C, Shkeda A, Storz SS, Sun H, Thibaud-Nissen F, Tolstoy I, Tully RE, Vatsan AR, Wallin C, Webb D, Wu W, Landrum MJ, Kimchi A, Tatusova T, DiCuccio M, Kitts P, Murphy TD, Pruitt KD. Reference sequence (RefSeq) database at NCBI: current status, taxonomic expansion, and functional annotation. Nucleic Acids Res. 2016 Jan 4;44(D1):D733-45. doi: 10.1093/nar/gkv1189. Epub 2015 Nov 8. PMID: 26553804; PMCID: PMC4702849.

[26] Tamara Goldfarb, Vamsi K Kodali, Shashikant Pujar, Vyacheslav Brover, Barbara Robbertse, Catherine M Farrell, Dong-Ha Oh, Alexander Astashyn, Olga Ermolaeva, Diana Haddad, Wratko Hlavina, Jinna Hoffman, John D Jackson, Vinita S Joardar, David Kristensen, Patrick Masterson, Kelly M McGarvey, Richard McVeigh, Eyal Mozes, Michael R Murphy, Susan S Schafer, Alexander Souvorov, Brett Spurrier, Pooja K Strope, Hanzhen Sun, Anjana R Vatsan, Craig Wallin, David Webb, J Rodney Brister, Eneida Hatcher, Avi Kimchi, William Klimke, Aron Marchler-Bauer, Kim D Pruitt, Françoise Thibaud-Nissen, Terence D Murphy, NCBI RefSeq: reference sequence standards through 25 years of curation and annotation, *Nucleic Acids Research*, 2024.

[27] Donovan H Parks, Maria Chuvochina, Christian Rinke, Aaron J Mussig, Pierre-Alain Chaumeil, Philip Hugenholtz, GTDB: an ongoing census of bacterial and archaeal diversity through a phylogenetically consistent, rank normalized and complete genome-based taxonomy, *Nucleic Acids Research*, Volume 50, Issue D1, 7 January 2022, Pages D785–D794, https://doi.org/10.1093/nar/gkab776

[28] Wang, Y., Pedersen, M.W., Alsos, I.G. et al. Late Quaternary dynamics of Arctic biota from ancient environmental genomics. Nature 600, 86–92 (2021).

[29] Thorvaldsdóttir, H., Robinson, J. T., & Mesirov, J. P. (2013). Integrative Genomics Viewer (IGV): high-performance genomics data visualization and exploration. *Briefings in bioinformatics*, *14*(2), 178–192. https://doi.org/10.1093/bib/bbs017

[30] Li H, Handsaker B, Wysoker A, Fennell T, Ruan J, Homer N, Marth G, Abecasis G, Durbin R; 1000 Genome Project Data Processing Subgroup. The Sequence Alignment/Map format and SAMtools. Bioinformatics. 2009 Aug 15;25(16):2078-9. doi: 10.1093/bioinformatics/btp352. Epub 2009 Jun 8. PMID: 19505943; PMCID: PMC2723002.

[31] Quinlan AR, Hall IM. BEDTools: a flexible suite of utilities for comparing genomic features. Bioinformatics. 2010 Mar 15;26(6):841-2. doi: 10.1093/bioinformatics/btq033. Epub 2010 Jan 28. PMID: 20110278; PMCID: PMC2832824.

[32] Ondov BD, Treangen TJ, Melsted P, Mallonee AB, Bergman NH, Koren S, Phillippy AM. Mash: fast genome and metagenome distance estimation using MinHash. Genome Biol. 2016 Jun 20;17(1):132. doi: 10.1186/s13059-016-0997-x.

[33] Kjær KH, Winther Pedersen M, De Sanctis B, De Cahsan B, Korneliussen TS, Michelsen CS, Sand KK, Jelavić S, Ruter AH, Schmidt AMA, Kjeldsen KK, Tesakov AS, Snowball I, Gosse JC, Alsos IG, Wang Y, Dockter C, Rasmussen M, Jørgensen ME, Skadhauge B, Prohaska A, Kristensen JÅ, Bjerager M, Allentoft ME, Coissac E; PhyloNorway Consortium; Rouillard A, Simakova A, Fernandez-Guerra A, Bowler C, Macias-Fauria M, Vinner L, Welch JJ, Hidy AJ, Sikora M, Collins MJ, Durbin R, Larsen NK, Willerslev E. A 2-million-year-old ecosystem in Greenland uncovered by environmental DNA. Nature. 2022 Dec;612(7939):283-291. doi: 10.1038/s41586-022-05453-y. Epub 2022 Dec 7.

[34] Bein B, Chrysostomakis I, Arantes LS, Brown T, Gerheim C, Schell T, Schneider C, Leushkin E, Chen Z, Sigwart J, Gonzalez V, Wong NLWS, Santos FR, Blom MPK, Mayer F, Mazzoni CJ, Böhne A, Winkler S, Greve C, Hiller M. Long-read sequencing and genome assembly of natural history collection samples and challenging specimens. bioRxiv [Preprint]. 2024 Sep 27:2024.03.04.583385. doi: 10.1101/2024.03.04.583385. Update in: Genome Biol. 2025 Feb 10;26(1):25. doi: 10.1186/s13059-025-03487-9.

[35] Chorlton SD. Ten common issues with reference sequence databases and how to mitigate them. Front Bioinform. 2024 Mar 15;4:1278228. doi: 10.3389/fbinf.2024.1278228.

[36] Breitwieser FP, Pertea M, Zimin AV, Salzberg SL. Human contamination in bacterial

genomes has created thousands of spurious proteins. Genome Res. 2019 Jun;29(6):954-960. doi: 10.1101/gr.245373.118. Epub 2019 May 7.

[37] Brait N, Hackl T, Lequime S., detectEVE: Fast, Sensitive and Precise Detection of Endogenous Viral Elements in Genomic Data. Mol Ecol Resour. 2025 Feb 12:e14083. doi: 10.1111/1755-0998.14083. Epub ahead of print. PMID: 39936183.

[38] Stephanie Dolenz, Tom van der Valk, Chenyu Jin, Jonas Oppenheimer, Muhammad Bilal Sharif, Ludovic Orlando, Beth Shapiro, Love Dalén, Peter D Heintzman, Unravelling reference bias in ancient DNA datasets, *Bioinformatics*, Volume 40, Issue 7, July 2024, btae436, https://doi.org/10.1093/bioinformatics/btae436

[39] Blanco-Melo D, Campbell MA, Zhu H, Dennis TPW, Modha S, Lytras S, Hughes J, Gatseva A, Gifford RJ. A novel approach to exploring the dark genome and its application to mapping of the vertebrate virus fossil record. Genome Biol. 2024 May 13;25(1):120. doi: 10.1186/s13059-024-03258-y. PMID: 38741126; PMCID: PMC11089739.

[40] Palatini U, Alfano N, Carballar-Lejarazu R, Chen XG, Delatte H, Bonizzoni M. Virome and nrEVEome diversity of Aedes albopictus mosquitoes from La Reunion Island and China. Virol J. 2022 Nov 18;19(1):190. doi: 10.1186/s12985-022-01918-8. Erratum in: Virol J. 2022 Dec 9;19(1):211. doi: 10.1186/s12985-022-01950-8.

[41] Mussig AJ, Chaumeil PA, Chuvochina M, Rinke C, Parks DH, Hugenholtz P. Putative genome contamination has minimal impact on the GTDB taxonomy. Microb Genom. 2024 May;10(5):001256. doi: 10.1099/mgen.0.001256. PMID: 38809778; PMCID: PMC11261887.

[42] Robinson AJ, Daligault HE, Kelliher JM, LeBrun ES, Chain PSG. Multiple Cases of Bacterial Sequence Erroneously Incorporated Into Publicly Available Chloroplast Genomes.

Front Genet. 2022 Jan 13;12:821715. doi: 10.3389/fgene.2021.821715. PMID: 35096026; PMCID: PMC8793683.

[43] Pochon Z, Bergfeldt N, Kırdök E, Vicente M, Naidoo T, van der Valk T, Altınışık NE, Krzewińska M, Dalén L, Götherström A, Mirabello C, Unneberg P, Oskolkov N. aMeta: an accurate and memory-efficient ancient metagenomic profiling workflow. Genome Biol. 2023 Oct 23;24(1):242. doi: 10.1186/s13059-023-03083-9. PMID: 37872569; PMCID: PMC10591440.

[44] Hübler R, Key FM, Warinner C, Bos KI, Krause J, Herbig A. HOPS: automated detection and authentication of pathogen DNA in archaeological remains. Genome Biol. 2019 Dec 16;20(1):280. doi: 10.1186/s13059-019-1903-0. PMID: 31842945; PMCID: PMC6913047.

Acknowledgments

NO, CMK and VEK are financially supported by Knut and Alice Wallenberg Foundation as part of the National Bioinformatics Infrastructure Sweden at SciLifeLab. PDH and FW were supported by the Knut and Alice Wallenberg Foundation (KAW 2021.0048 [PDH, FW] and KAW 2022.0033 [PDH]). EJ is supported by the Swedish Research Council (VR 2020-04808). TvdV, BG, CJ and SLC acknowledge support from the SciLifeLab and Wallenberg Data Driven Life Science Program [KAW 2020.0239].

Data availability

NCBI RefSeq reference genomes, release 213 (from 23rd of July 2022), were obtained from https://ftp.ncbi.nlm.nih.gov/refseq/release/, and NCBI GenBank reference genomes were downloaded from https://ftp.ncbi.nih.gov/genomes/genbank/. The NCBI accession ids of the reference genomes used for the analysis are available in the Supplementary Tables 1-6.

GTDB dataset of microbial reference genome assemblies (bacterial and archaeal), release 214, can be accessed at https://data.gtdb.ecogenomic.org/releases/release214/214.0/, and the PhyloNorway project DataverseNO V1 Nordic plant contig-level reference genomes are available at https://doi.org/10.18710/3CVQAG. The empirical datasets [28, 32] were obtained from the EMBL-ENA under project accession PRJEB43822 and PRJEB55522, respectively. The adapter-removed reads for the Arctic sample were downloaded from ftp://ftp.sra.ebi.ac.uk/vol1/run/ERR645/ERR6458938/cr9_67.truncated.fastq.gz, and the adapter-removed reads for the Greenland sample were downloaded from the ftp-address: ftp://ftp.sra.ebi.ac.uk/vol1/run/ERR104/ERR10493316/69_B2_100_L0_KapK-12-1-35_Ext-12_Lib-12.pair1.truncated.gz. The BED-files with coordinates of microbial-like sequences for each group of eukaryotic organisms can be downloaded from the SciLifeLab Figshare repository https://doi.org/10.17044/scilifelab.28380476. The workflow together with the pre-built datasets of microbial and human pseudo-reads and other helping files is available via~~at~~ the SciLifeLab Figshare repository https://doi.org/10.17044/scilifelab.28491956 as well as Zenodo repository https://doi.org/10.5281/zenodo.16788411 and workflowhub.eu repository https://doi.org/10.48546/workflowhub.workflow.1846.1. We have also registered the workflow at SciCrunch.org with the following Research Resource Identification Initiative ID (RRID): SCR_027305, and bio.tools with the biotools:genex_workflow unique resource ID.

Availability of source code and requirements

The source codes of the workflow together with a ~~A~~ comprehensive vignette covering the workflow usage and interpretation of the output are ~~is~~ available at the GitHub repository https://github.com/NikolayOskolkov/MCWorkflow. Custom scripts used for performing the analysis and computing the figures for the manuscript are explained in detail in the Supplementary Material S6, and are available in~~available at~~ the GitHub repository https://github.com/NikolayOskolkov/MCManuscript.

Main Figures

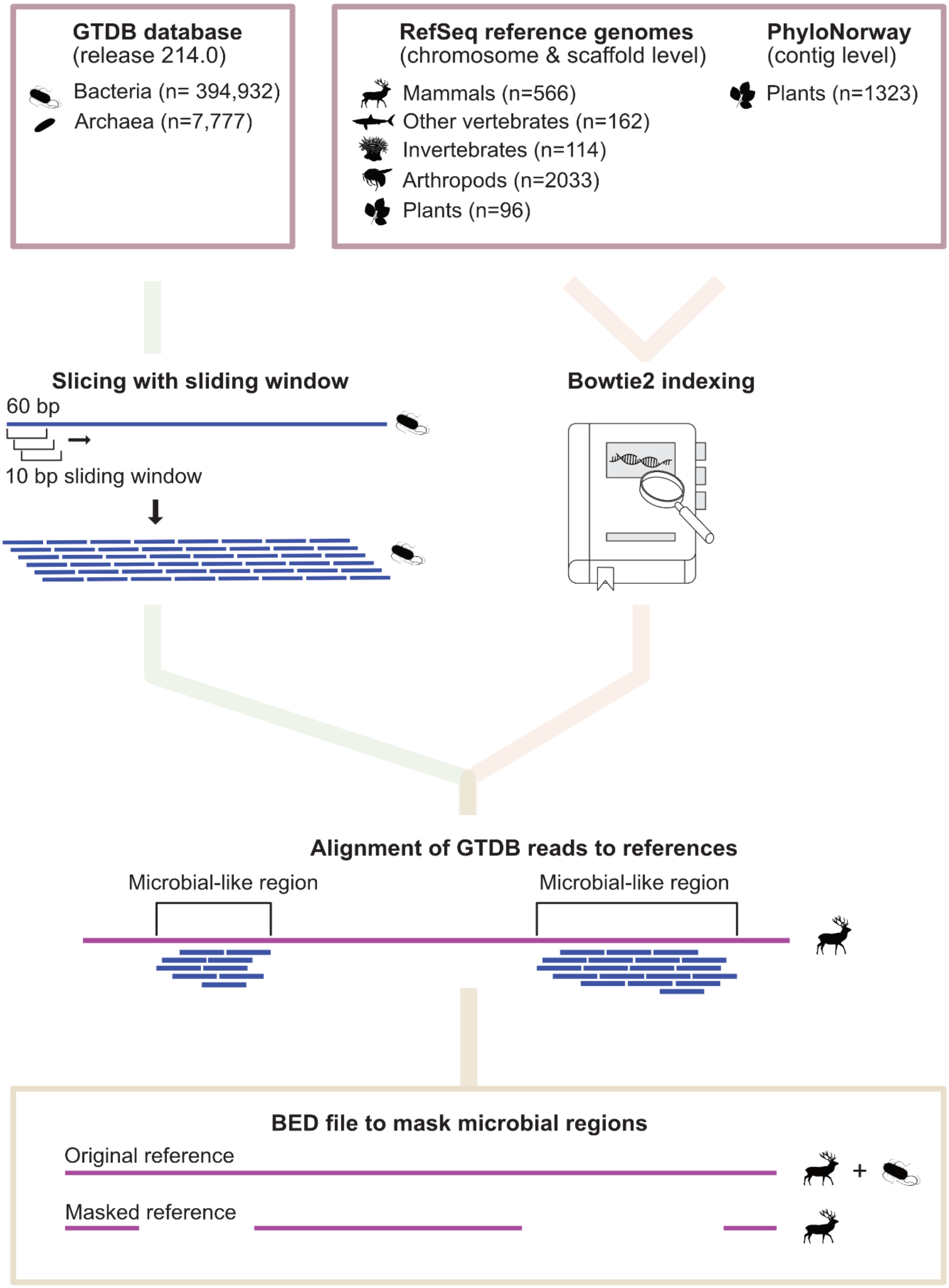

Figure 1. The workflow for detection of microbial-like sequences in eukaryotic reference genomes. Assemblies at different levels from different databases were subjected to the workflow. Microbial reference genomes from GTDB were fragmented into pseudo-reads (60 bp) and aligned to these genomes, retaining up to 10 multi-mappers to increase detection sensitivity. Microbial-like~~Contaminated~~ regions were identified, annotated, and visualised, with microbial abundance summarised in BED files and validated using IGV.

ORGANISM	REFID	CONTIG	START	END	LENGTH	NREADS	MICR1	MICR2	MICR3
Arctocephalus gazella	GCA_900500725.1	UIRR01000886.1	127	246	119	49387	56_reads_Moritella_sp018219455	52_reads_Moritella_sp018219155	47_reads_Moritella_marina
Arctocephalus gazella	GCA_900500725.1	UIRR01000886.1	252	895	643	657342	535_reads_Moritella_sp018219455	519_reads_Vibrio_echinoideorum	499_reads_Photobacterium_p
Arctocephalus gazella	GCA_900500725.1	UIRR01000886.1	1017	1222	205	42914	132_reads_Moritella_sp018219455	127_reads_Photobacterium_swingsii	124_reads_Photobacterium_to
Arctocephalus gazella	GCA_900500725.1	UIRR01000886.1	1268	2493	1225	754795	945_reads_Vibrio_parahaemolyticus	922_reads_Photobacterium_toruni	921_reads_Photobacterium_s
Arctocephalus gazella	GCA_900500725.1	UIRR01000886.1	2719	5781	3062	2079142	1534_reads_Kosakonia_sp000410515	1482_reads_Photobacterium_toruni	1476_reads_Moritella_sp0182
Arctocephalus gazella	GCA_900500725.1	UIRR01000886.1	5878	6190	312	1695	24_reads_Escherichia_sp004211955	22_reads_Escherichia_ruysiae	22_reads_Escherichia_albertii

Table 1. Example of BED-file with coordinates of microbial-like regions ~~contamination~~ of *Arctocephalus gazella*, reference genome GCA_900500725.1. The columns of the BED-file have the following notations: ORGANISM - scientific name of the organism, REFID - identification code of the reference genome, CONTIG - identification code of chromosome / scaffold / contig, START - start position of the segment of microbial-like region ~~contamination~~, END - end position of the segment of microbial-like region ~~contamination~~, LENGTH - length of the segment of microbial-like region ~~contamination~~, NREADS - number of pseudo-reads supporting the microbial-like segment~~segment of microbial contamination~~, MICR1-3 - top 3 most abundant microbes contributing to the microbial-like segment~~segment of microbial contamination~~; for example, the element “56_reads_Moritella_sp018219455” within MICR1 column denotes that *Moritella* sp018219455 was the most abundant microbe from that segment with 56 pseudo-reads.

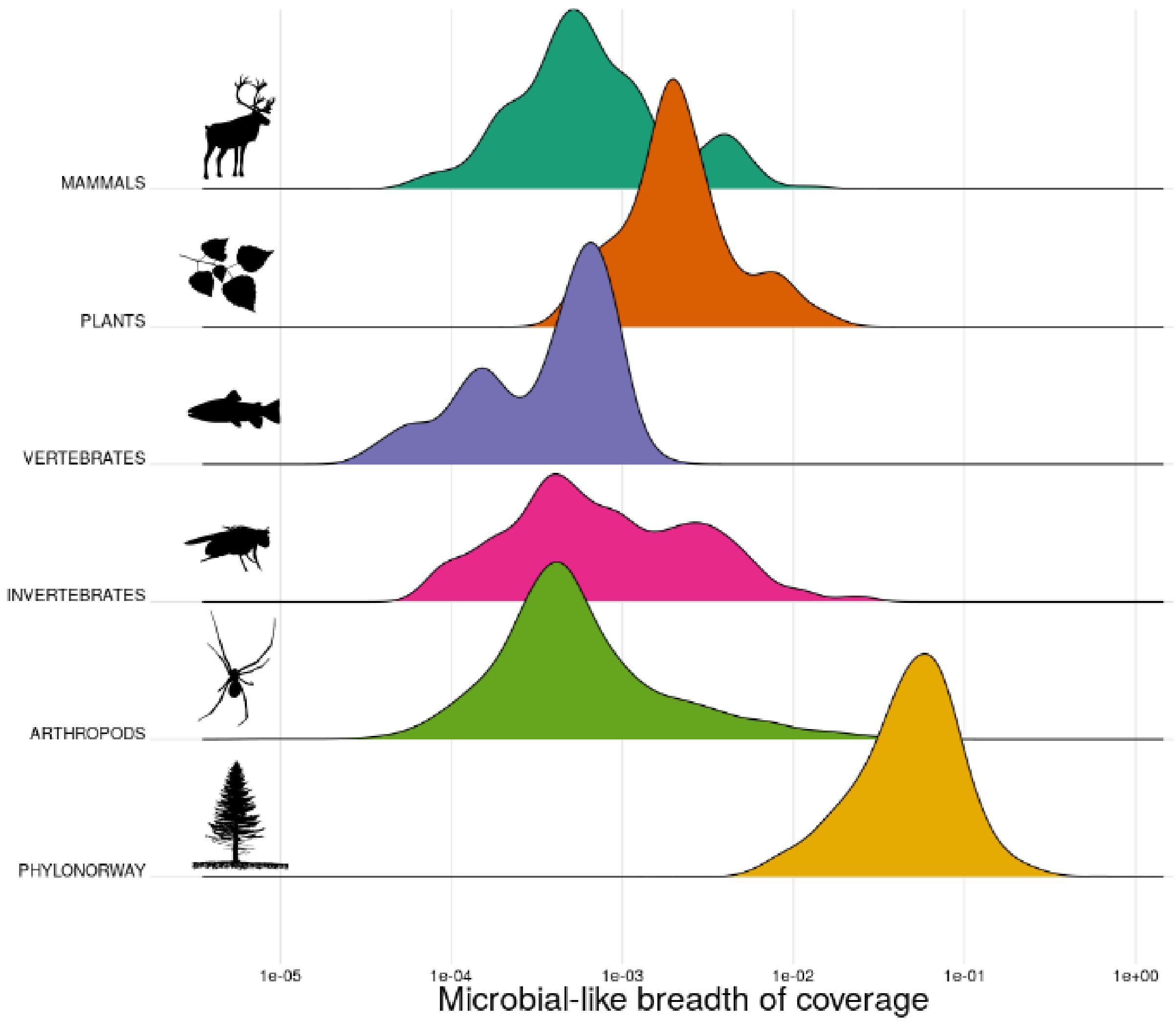

Figure 2. Distribution of microbial-like breadth of coverage (fraction of covered reference nucleotides) across the six reference genome groups from PhyloNorway or NCBI RefSeq. Mammalian genomes are represented by the genome with the highest contig N50 for each species sourced from the NCBI assembly database, which includes but is not limited to genomes from Refseq. The x-axis of the plot is on a log-scale. The y-axis represents the density estimates of the six datasets.

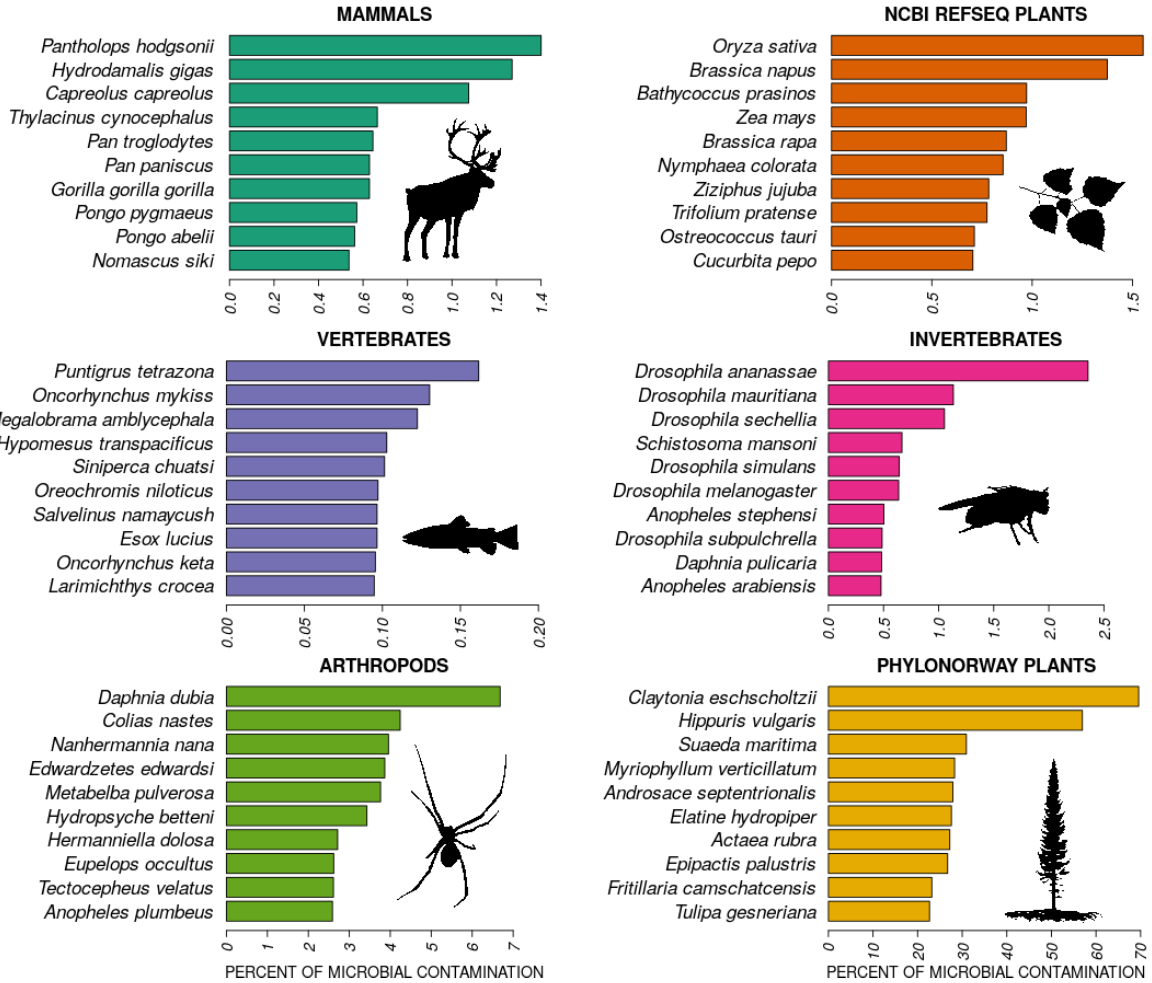

Figure 3. Reference genomes with the highest levels of microbial-like sequences for each genome group. Complete information is available in Supplementary Tables 1-6.

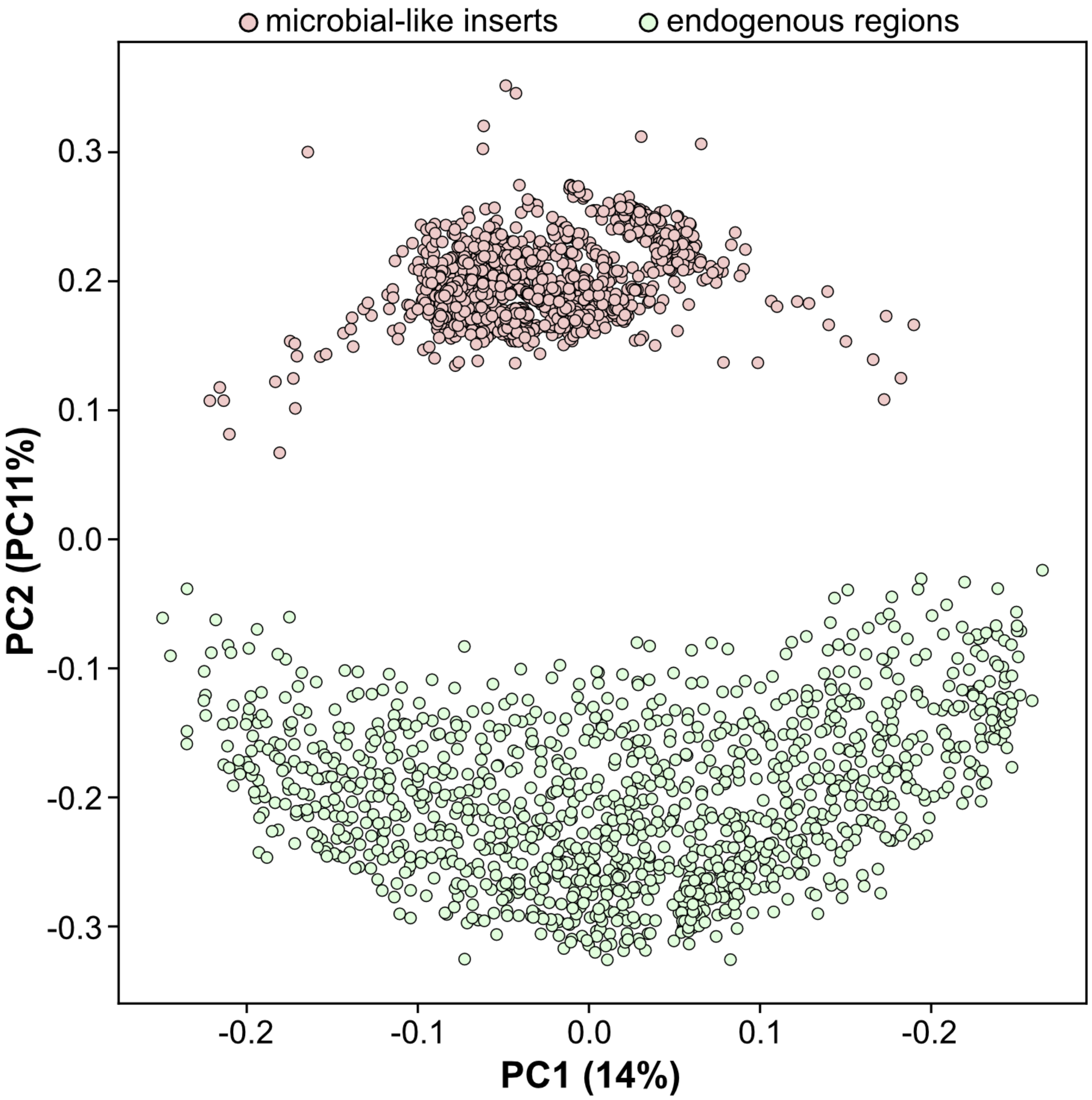

Figure 4. Principal Component Analysis (PCA) visualization of genomic pairwise distances between presumed endogenous (plant DNA) and exogenous (microbial-like) regions in the PhyloNorway dataset detected in this study. Each dot represents a single genome, with the light red dots representing regions identified as microbial-like and green dots as endogenous. The distinct clustering of endogenous and exogenous genomic segments suggests differentiation in their k-mer composition.

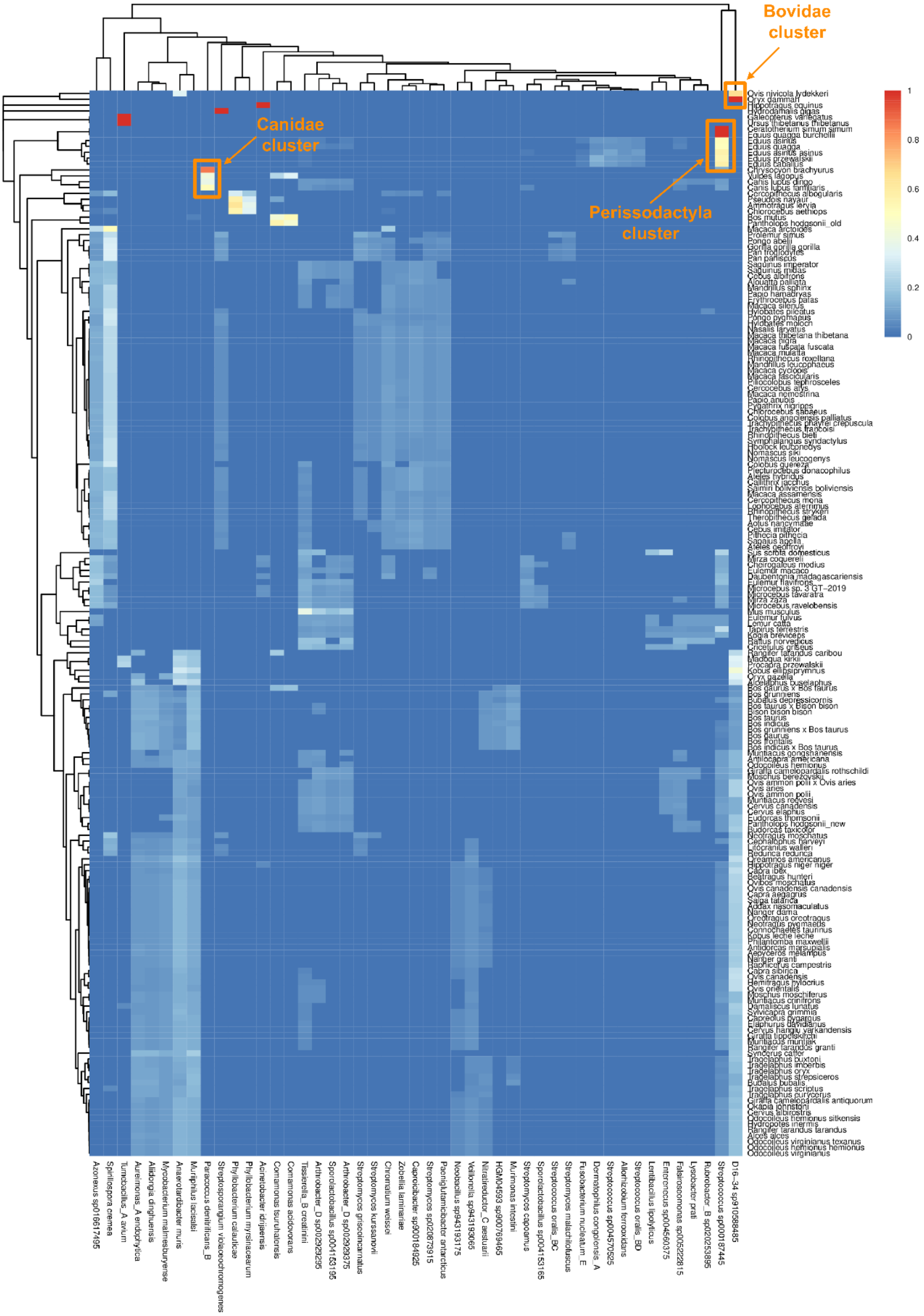

Figure 5. Abundance heatmap of microbial-like sequences across mammalian reference genomes. The columns represent microbial taxa contributing to the reference genomes of mammals displayed as rows. The color gradient indicates normalized abundance of microbial-like sequences (0-lowest,1-highest).

Supplementary Material

S1. Alignment of microbial pseudo-reads to eukaryotic reference genomes

To generate microbial pseudo-reads, we utilized 394,932 bacterial and 7,777 archaeal reference genome sequences from the Genome Taxonomy Database (GTDB) release 214 [27] downloaded from https://data.gtdb.ecogenomic.org/releases/release214/214.0/. Each of the microbial references was fragmented into 60 bp long segments using a sliding window with a 10 bp step. The 60 bp length of microbial pseudo-reads was chosen to provide sufficient specificity of matching to eukaryotic references as it is twice as long as the conventional 30 bp lower threshold of specificity across organisms in the tree of life [14, 15]. As a result, we generated a set of $2.6*10^{10}$ microbial pseudo-reads. The eukaryotic reference genomes were individually indexed with Bowtie2 [16] using the following command line:

bowtie2-build --large-index reference_genome.fna.gz reference_genome.fna.gz --threads 20

Afterwords, microbial pseudo-reads were aligned to each indexed eukaryotic reference genome, and the alignments were sorted and indexed with *samtools* [30] using the following command lines:

bowtie2 --large-index -f -k 10 -x reference_genome.fna.gz --end-to-end --threads 20 --very-sensitive -U microbial_reads.fna.gz | samtools view -bS -F 4 -h -@ 20 - | samtools sort -@ 20 - > MicrReads_aligned_to_reference_genome.bam

samtools index -c MicrReads_aligned_to_reference_genome.bam

It is reasonable to assume that some microbial sequences can map to multiple loci in eukaryotic reference genomes. Therefore, in order to increase sensitivity of discovery of

microbial-like~~contaminated~~ regions, we allowed up to 10 multi-mapping pseudo-reads to be kept in the alignments (the flag *-k 10* in the Bowtie2 command line above). For estimating the optimal number of multi-mappers to keep, we performed alignments of microbial pseudo-reads to Gray short-tailed opossum (*Monodelphis domestica*, GCA_027887165.1) and African elephant (*Loxodonta africana*, GCF_000001905.1) reference genomes while varying the maximum number of multi-mapped positions to retain for a read (0, 5, 10, 25, or 50 positions). We recorded the total number of both mapped reads and discovered regions of microbial-like sequences (Supplementary Figure 1). We observed that both sensitivity metrics for both organisms saturated at ~5-10 multi-mappers. We therefore decided to allow up to 10 multi-mapping pseudo-reads to be kept when performing alignments.

The microbial-like regions were detected by computing the breadth of coverage (boc) from the alignments with *samtools depth* [30] as

samtools depth -g 0x100 -a MicrReads_aligned_to_reference_genome.bam > boc.txt

Here, we used the *-g 0x100* flag to account for contributions from multi-mapping microbial pseudo-reads to the total coverage.

It is important to mention that Bowtie2 has a special non-trivial scoring system to determine whether a read will be mapped to a reference. The scoring system is not solely based on the exact number of mismatches but includes multiple other metrics such as base quality, gaps, clipping etc. Nevertheless, Bowtie2 prioritizes high-scoring (i.e. more similar) alignments, and heavily penalizes divergence. Empirically, we can see that a 60 bp read with more than ~6 mismatches (average nucleotide identity ANI=90%) will usually fail to align, even under --very-sensitive –end-to-end. For example, tested on RefSeq plants and PhyloNorway references with aligned GTDB pseudo-reads, we observe on average 4 ± 0.4 and 4 ± 0.2 mismatches per read, respectively, i.e. the similarity of ANI=93%. Taking into account that a typical ANI

threshold for ancient metagenomics projects is set much lower, i.e. down to 85%, due to DNA damage [43, 44], we assume the risk of non-specific alignments with the Bowtie2 mapping parameters used in this study is low.

S2. Following up the microbial-like and endogenous regions within *Hippuris vulgaris* reference genome assembly from the PhyloNorway dataset

We used the annotation file *PhyloNorwayContigs_acc2TaxaID.txt* provided together with the PhyloNorway dataset *merged_PhyloNorway.fna* (merged individual FASTA-files) available at https://doi.org/10.18710/3CVQAG for retrieving 433,631 contig ids corresponding to the taxid of 39321 of the *Hippuris vulgaris* species. The corresponding reference sequences for each contig id of *Hippuris vulgaris* were extracted with *seqtk subseq* function from the seqtk toolkit https://github.com/lh3/seqtk, and saved as *39321.fna* FASTA-file using the following command lines:

```
grep -w 39321 PhyloNorwayContigs_acc2TaxaID.txt | cut -f2 > contig_ids_39321.txt
seqtk subseq merged_PhyloNorway.fna contig_ids_39321.txt > 39321.fna
```

Further, after we have inferred the coordinates of microbial-like regions of *Hippuris vulgaris* with our method, and generated the *micr_coords_39321.bed* BED-file, which can be retrieved from the integrated BED-file for all PhyloNorway reference genomes at SciLifeLab Figshare https://doi.org/10.17044/scilifelab.28380476, we proceeded with *bedtools getfasta* [31], and extracted the *Hippuris vulgaris* reference sequences corresponding to the microbial-like regions:

```
bedtools getfasta -fi 39321.fna -bed micr_coords_39321.bed -fo micr_seqs_39321.fna
```

Next, we applied *samtools* [30], *bedtools complement* [31] and *bedtools getfasta* to group the

remaining (presumed endogenous) reference sequences of *Hippuris vulgaris* in a separate FASTA-file:

```
samtools faidx 39321.fna && cut -f1,2 39321.fna.fai > 39321.fai
bedtools complement -i micr_coords_39321.bed -g 39321.fai > endo_coords_39321.bed
bedtools getfasta -fi 39321.fna -bed endo_coords_39321.bed -fo endo_seqs_39321.fna
```

In order to explore whether the microbial-like reference sequences of *Hippuris vulgaris* cluster together with bacterial or plant reference genomes, we computed the *k*-mer pairwise distances with Mash [32] using 91 NCBI RefSeq plant and 100 random bacterial NCBI RefSeq reference genomes as well as the two additional *Hippuris vulgaris* FASTA-files corresponding to endogenous and microbial-like sequences. We performed hierarchical clustering with the *hclust* function in R using the Ward method (Supplementary Figure 3). We observed that the inferred microbial-like sequences of *Hippuris vulgaris* were clustering together with bacterial NCBI RefSeq reference genomes while endogenous sequences grouped with plant reference genomes.

Next, for each of 433,631 contigs of *Hippuris vulgaris* we computed the fraction of microbial-like sequences using the coordinates, *micr_coords_39321.bed*, of microbial-like regions. We plotted the histogram, Supplementary Figure 4, of microbial-like fractions with *plot_hist.R* available at https://github.com/NikolayOskolkov/MCManuscript.

After we have explored the microbial-like content of the *Hippuris vulgaris* reference genome assembly from the PhyloNorway dataset, we aimed at investigating how this could affect the read assignment in [28] and [33] studies reporting *Hippuris* prevalence at certain periods of history. We downloaded adapter-removed reads in the form of FASTQ-files corresponding to two samples from [28] (“Arctic sample”) and [33] (“Greenland sample”), where high *Hippuris* abundance was reported in the original studies:

```
wget ftp://ftp.sra.ebi.ac.uk/vol1/run/ERR645/ERR6458938/cr9_67.truncated.fastq.gz
wget ftp://ftp.sra.ebi.ac.uk/vol1/run/ERR104/ERR10493316/69_B2_100_L0_KapK-12-1-35_Ext-12_Lib-12.pair1.truncated.gz
```

Since both mammalian and plant organisms were reported for those two samples in the original studies [28] and [33], we implemented the competitive mapping approach to disentangle the mammalian and plant reads, and proceeded with the reads that align uniquely to the *Hippuris vulgaris* reference. To perform the competitive mapping, we built Bowtie2 [16] index of the *Hippuris vulgaris* reference genome concatenated with Asian Elephant (EleMax1, GCF_024166365.1) and Human (GRCH38, GCF_000001405.40) reference genome. Next, we performed Bowtie2 alignment of the downloaded reads to the indexed composite reference, and extracted only the reads mapping uniquely to the *Hippuris vulgaris* reference genome:

```
cat EleMax1.fna Human38.fna 39321.fna > EleMax_Human_Hippuris.fna
bowtie2-build --large-index EleMax_Human_Hippuris.fna EleMax_Human_Hippuris.fna --threads 20

bowtie2 --large-index -x EleMax_Human_Hippuris.fna --end-to-end --very-sensitive --threads 20 -U cr9_67.truncated.fastq.gz | samtools view -bS -q 1 -h -@ 20 - | samtools sort -@ 20 - > cr9_67.aligned_to_EleMax_Human_Hippuris.bam

awk '{print $1, 1, $2}' OFS='\t' genome_39321.fna.fai > genome_39321.fna.bed
samtools view -L genome_39321.fna.bed -q 1 -h -@ 20 -o cr9_67.aligned_to_39321.bam cr9_67.aligned_to_EleMax_Human_Hippuris.bam
```

From the alignment BAM-file, we retrieved the ids of contigs with at least one read aligned,

and using the BED-coordinates, *micr_coords_39321.bed*, of microbial-like regions for *Hippuris vulgaris*, we computed the fraction of microbial-like sequences corresponding to each contig with at least one aligned read (Supplementary Figure 5).

To understand how often the aligned reads overlap with the inferred microbial-like regions of *Hippuris vulgaris*, we extracted the coordinates of aligned reads with *bedtools bamtobed*:

```
bedtools bamtobed -i cr9_67.aligned_to_39321.bam >  cr9_67.coords_aligned_reads.bed
```

and calculated the number of intersections between the coordinates of the aligned reads and the coordinates of inferred microbial-like regions using *bedtools closest* with the *-d* (report distance) flag and custom bash / awk command lines:

```
bedtools closest -a cr9_67.coords_aligned_reads.bed -b micr_coords_39321.bed -d > cr9_67.coords_aligned_reads_annotated_with_closest_micr_like_region.bed
cut -f7 cr9_67.coords_aligned_reads_annotated_with_closest_micr_like_region.bed | awk '{if($1==0)print $0}' | wc -l >> number_of_observed_intersects.txt
```

We discovered that the vast majority of aligned reads, i.e. 116,483 out of 119,854 reads mapped in the Arctic sample (i.e. 97%) and 1,014,237 out of 1,367,627 reads (i.e. 74%) in the Greenland sample, intersected with the regions previously identified as microbial-like in the *Hippuris vulgaris* reference. To check whether this represents a significant enrichment compared to random read positioning, we performed 300 random replacements of the aligned reads, and every time counted the number of their intersects with the coordinates of microbial-like regions using a custom R script, please see the whole procedure in the R script *shuffle_reads.R* available at https://github.com/NikolayOskolkov/MCManuscript. We produced the Supplementary Figure 6 using the recorded numbers of intersects between the randomly placed reads and microbial-like regions and plotted them with *plot_hist.R* script.

S3. Microbial-like sequence composition of reference genomes from NCBI RefSeq plants, invertebrates, non-mammalian vertebrates, arthropods and PhyloNorway plants

We used samtools [30] and custom bash and R scripts for annotating the eukaryotic reference genomes with microbial taxonomic names corresponding to the most abundant microbial-like sequences. The most abundant (top 10 for each organism) microbes and eukaryotic references with the highest levels (top 200) of microbial-like regions were summarized via a heatmap computed by the *pheatmap* R package, demonstrating microbial co-occurrence in some groups of mammalian organisms (Figure 5). By analogy with the mammalian microbial-like sequences abundance heatmap, similar clustering patterns can be observed in microbial-like sequence composition of NCBI RefSeq plants, invertebrates, non-mammalian vertebrates, arthropods and PhyloNorway plants, shown respectively in Supplementary Figures 7-11.

For example, *Stenotrophomonas* sp003504055 is shared at high and moderately high abundance across two clusters comprising the fruit fly genus *Drosophila* (Supplementary Figure 8). Similarly, for non-mammalian vertebrate taxa, *Methylocystis* sp011058845 is highly abundant and shared across freshwater fishes such as northern pike (*Esox lucius*, GCF_011004845.1), lake whitefish (*Coregonus clupeaformis*, GCF_020615455.1), lake trout (*Salvelinus namaycush*, GCF_016432855.1), Atlantic salmon (*Salmo salar*, GCF_905237065.1), brown trout (*Salmo trutta*, GCF_901001165.1), chum salmon (*Oncorhynchus keta*, GCF_012931545.1), rainbow trout (*Oncorhynchus mykiss*, GCF_013265735.2), coho salmon (*Oncorhynchus kisutch*, GCF_002021735.2), sockeye salmon (*Oncorhynchus nerka*, GCF_006149115.2), pink salmon (*Oncorhynchus gorbuscha*, GCF_021184085.1) and chinook salmon (*Oncorhynchus tshawytscha*, GCF_018296145.1) (Supplementary Figure 9).

There are also a few clear clusters of arthropod reference genomes that share common microbial-like sequences. For instance, *Enterobacter* sp000493015 is commonly present among reference genomes of butterflies, moths and wasps such as Labrador sulphur (*Colias nastes*, GCA_907164665.1), Asiatic rice borer (*Chilo suppressalis*, GCA_902850365.2), parasitic wasp (*Cotesia vestalis*, GCA_000956155.1), and queen butterfly (*Danaus gilippus*, GCA_018231785.1), whereas *Sphingomonas* sp017418975 is prevalent and shared in reference genomes of soil and leaf associated arthropods such as beetle mite (*Nanhermannia comitalis*, GCA_034697665.1), oribatid mites (*Nothrus palustris*, GCA_034697745.1; *Malaconothrus monodactylus*, GCA_034697245.1), terrestrial cave isopod (*Haplophthalmus danicus*, GCA_034700045.1) and springtail (*Isotomurus plumosus*, GCA_034696705.1) (Supplementary Figure 10).

In contrast to the NCBI reference genomes, the PhyloNorway dataset does not demonstrate obvious commonalities in terms of co-occurrence of microbial-like sequences. Instead, there is at least one group of microbes including *JC017* sp004296775, *Solirubrobacter* sp003344625, *Frankia californiensis*, *Frankia* sp917627385, *Frankia meridionalis*, *Geodermatophilus endophyticus_A*, *Spirillospora cremea*, *Modestobacter lapidis*, *Geodermatophilus* sp019799925, *Streptomyces capoamus*, *SACZ01* sp023369685, *Ancylomarina* sp009669305, which is shared across nearly all plant genome assemblies in the PhyloNorway dataset (Supplementary Figure 11). This reflects, in our opinion, the common sample storage, processing, and sequencing routines used for generating these genome assemblies rather than shared ecological or evolutionary factors.

S4. Discovering microbial-like regions with microbial RefSeq pseudo-reads

In addition to the microbial pseudo-reads produced from the GTDB database, which included only bacterial and archaeal reference genomes, we have also generated a set of $1.1*10^{10}$

nucleotide sequences using the NCBI RefSeq microbial database, release 213. The latter contained 39,760 microbial reference genomes including 28,044 bacteria, 11,220 viruses, 459 archaea, 33 fungi and 4 protozoa. The RefSeq microbial pseudo-reads were prepared in the same way as described in the Methods section. The accuracy of RefSeq pseudo-reads preparation was validated by aligning them to 25 randomly selected RefSeq reference sequences which yielded a median breadth of coverage of 97.2%, which supports our expectation that the RefSeq reference sequences looked composed almost entirely of microbial-like sequences. Despite the potential redundancy (e.g. some bacteria such as *Escherichia coli* may have multiple versions of a reference genome), the RefSeq microbial pseudo-reads may be useful for discovering viral-like sequences in eukaryotic reference genomes. This analysis can be used complementary to the detection of microbial-like sequences with the GTDB pseudo-reads within the main workflow. Both GTDB and RefSeq microbial pseudo-reads are publicly available together with the workflow files via the SciLifeLab Figshare https://doi.org/10.17044/scilifelab.28380476. We found that in most cases, either the coverage by GTDB and RefSeq pseudo-reads had good agreement (Supplementary Figure 12), or the GTDB pseudo-reads provided higher resolution of discovery of microbial-like sequences (Supplementary Figures 13 and 14). Nevertheless, viral-like regions within eukaryotic genomes can only be inferred using the RefSeq microbial pseudo-reads.

When using this workflow with RefSeq (viral) pseudo-reads, it is important to carefully assess genomic fragments classified as viral-like sequences, as they may not represent free-living viral contaminants, but rather endogenous viral elements (EVEs), which are "fossilised" viral sequences integrated into the host genome. Establishing EVEs is a challenging problem and requires careful analysis to confirm that these sequences are not of exogenous viral origin [37]. Our approach can be used for detecting only recent EVEs, as our workflow relies on a mapping tool that performs poorly with highly divergent DNA sequences [38], a common feature of EVEs. Homology-based methods therefore offer a more effective alternative for

detecting distant viral relationships due to their greater flexibility and sensitivity [37, 39, 40].

S5. Discovering human-like regions with human hg38 pseudo-reads

We have pre-computed human hg38 pseudo-reads, which resulted in a set of $3.2*10^{8}$ nucleotide sequences, and made them publicly available together with the workflow (see also Data and Code Availability). The workflow parameters have been updated to include an option for using these pre-computed human pseudo-reads, enabling users to detect “human-like” regions in prokaryotic or eukaryotic reference genomes. As a proof of concept, we applied the workflow to the *Spirometra erinaceieuropaei* (parasitic tapeworm) reference genome GCA_000951995.1, previously suspected of containing human contamination [9]. Our analysis revealed that more than 0.1% of the genome contains human-like sequences, including over 50 scaffolds—some up to 1.7 kbp in length—with 100% breadth of coverage by human pseudo-reads. The total length of detected human-like sequences amounts to 1.4 Mbp. An IGV visualization of one of the fully covered scaffolds is shown in the Supplementary Figure 15. In addition, screening the *Bathycoccus prasinos* (green algae) reference genome GCF_002220235.1 revealed over 236,000 aligned human pseudo-reads, covering approximately 0.2% of the genome. The total length of these potentially exogenous regions amounts to 37 kbp. This testing demonstrates that the workflow can be extended beyond discovering only microbial-like sequences and serve as a tool for detecting exogenous regions within a given reference in general.

S6~~S5~~. Scripts used for computing main and supplementary figures

All scripts and input files used in this study for computing main and supplementary figures are available at the GitHub repository https://github.com/NikolayOskolkov/MCManuscript. Main Figures 2, 3, 4 and 5 were plotted in R using *ridgeline.R*, *make_cont_barplots.R*, *plotPCA.py* and *micr_abund_heatmap.R* scripts, respectively. Supplementary Figures 1 and 3 were

produced using *multimappers.R* and *cluster_plants_plus_bacteria_plus_hippuris.R*, respectively. The output of the latter script, i.e. the dendrogram in Newick format, is available at the GitHub as *dendrogram.nwk* file. Supplementary Figures 4-6 were plotted in R using *plot_hist.R* script. The heatmaps for Supplementary Figures 7-11 were computed with *micr_abund_heatmap.R* script. The input files for computing the heatmaps are available in the *micr_abundance* folder in the GitHub repository. Finally, the Supplementary Figure 14 was calculated in R with *RefSeq_vs_GTDB_discovered_regions.R* script.

Supplementary Figures

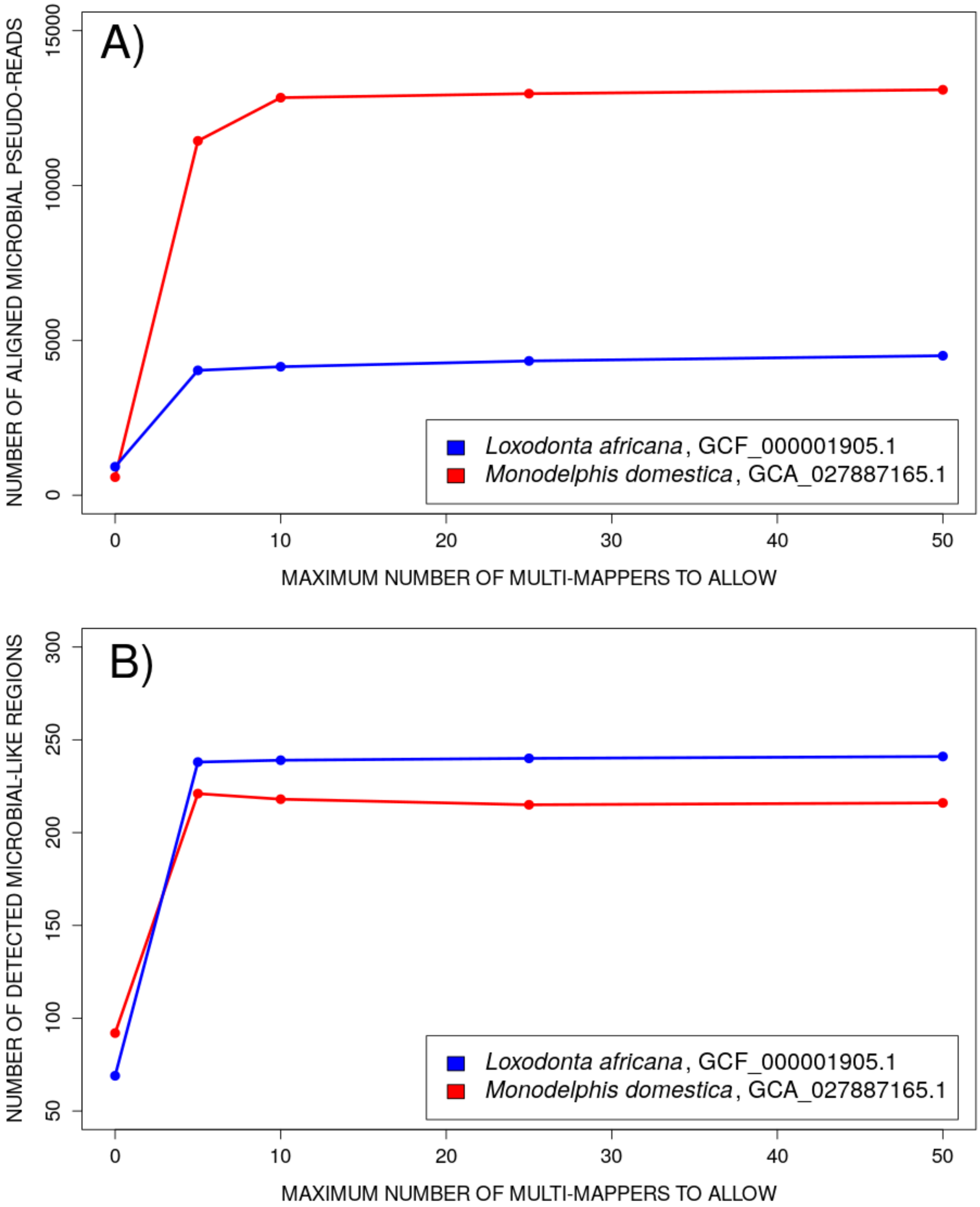

Supplementary Figure 1. Sensitivity of discovery of microbial-like regions when aligning microbial pseudo-reads to Gray short-tailed opossum (*Monodelphis domestica*, GCA_027887165.1) and African elephant (*Loxodonta africana*, GCF_000001905.1) reference genomes with different numbers of multi-mapping pseudo-reads to retain.

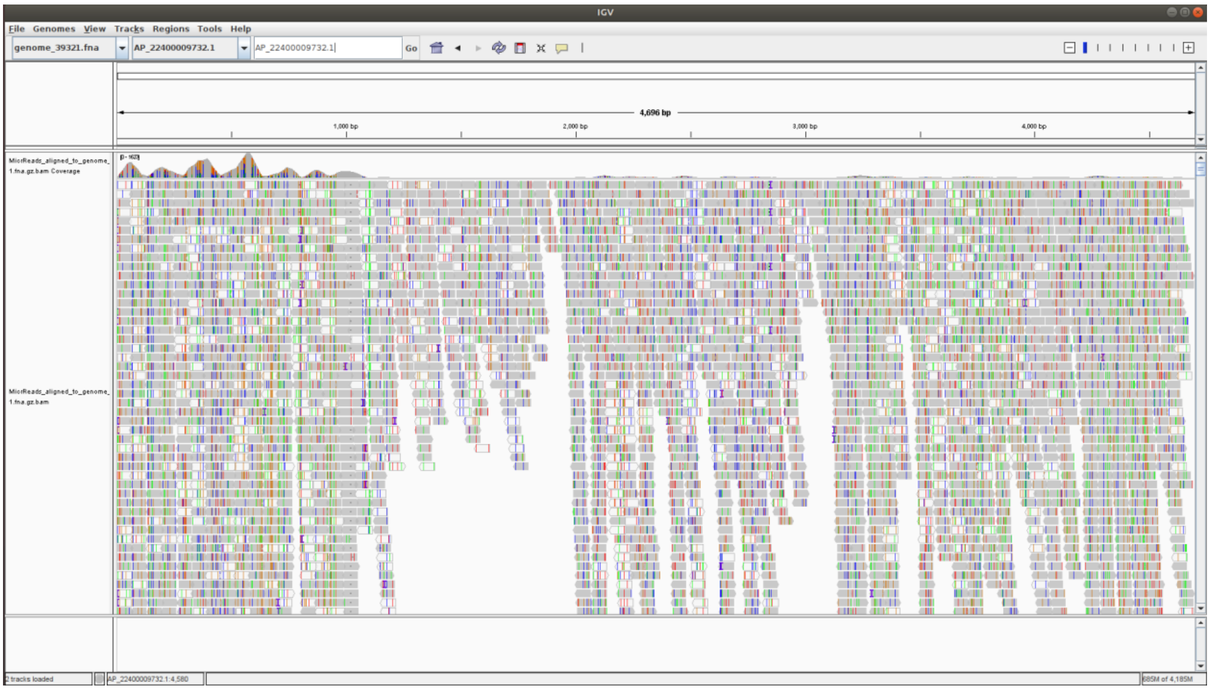

Supplementary Figure 2. Example of coverage of detected exogenous regions by mapped bacterial pseudo-reads to the *Hippuris vulgaris* reference genome from the PhyloNorway dataset. The visualization is performed using the Integrative Genomics Viewer (IGV).

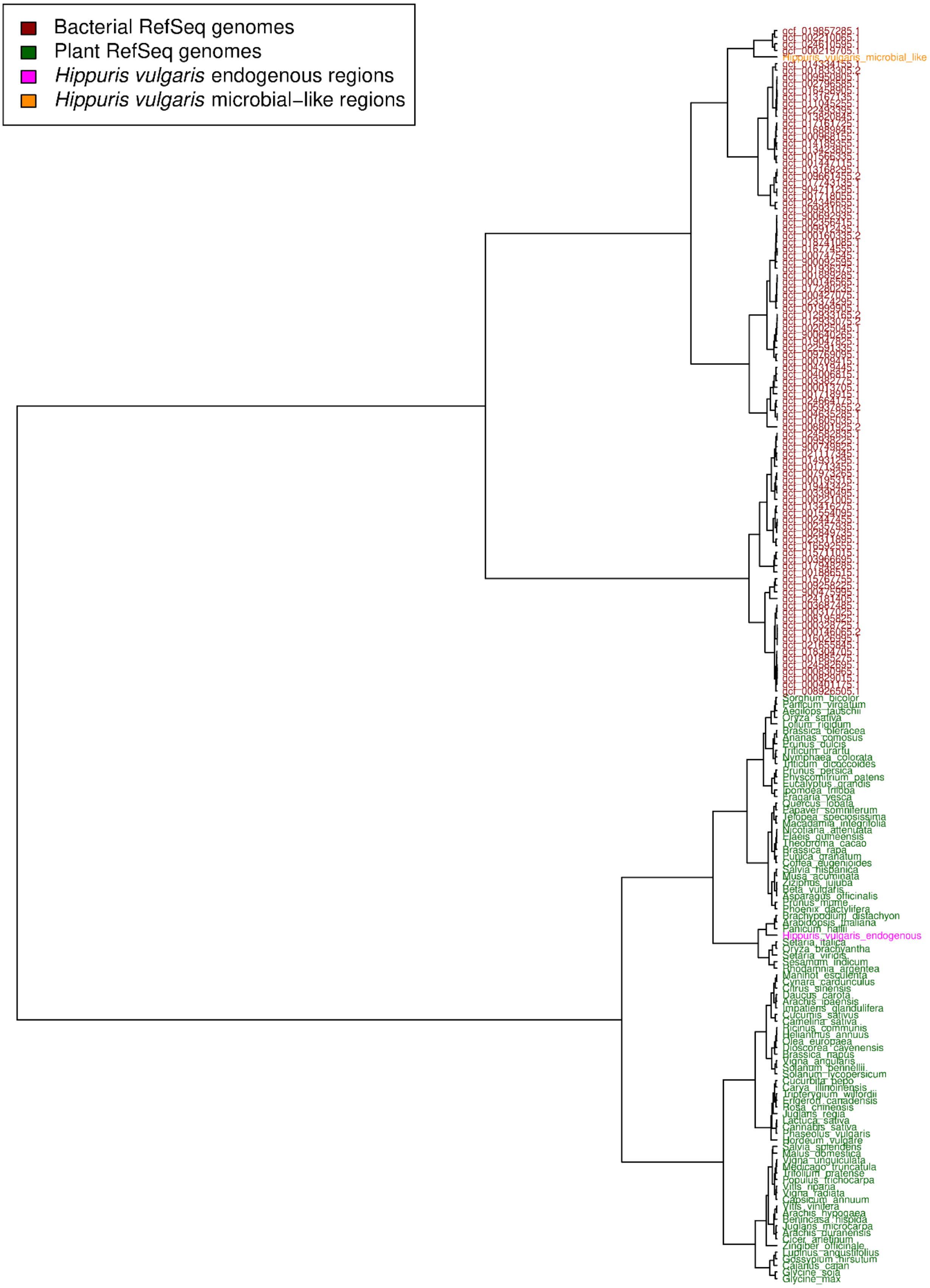

Supplementary Figure 3. *Hippuris vulgaris* microbial-like (presumed exogenous) and remaining (presumed endogenous) segments from the PhyloNorway dataset projected on the hierarchical clustering dendrogram of NCBI RefSeq plants and bacteria computed using Mash [32] pairwise distances based on the *k*-mer composition of their reference genomes.

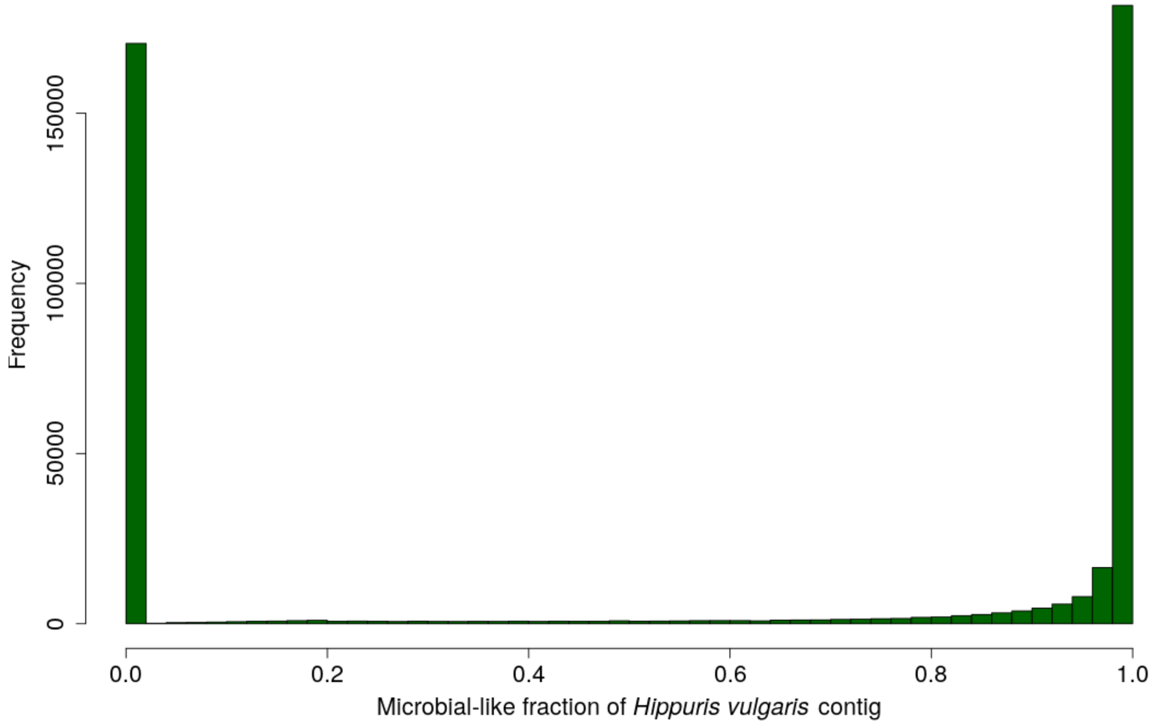

Supplementary Figure 4. Distribution of microbial-like fractions of 433,631 contigs of *Hippuris vulgaris* from the PhyloNorway dataset profiled ~~for microbial contamination~~ in our analysis.

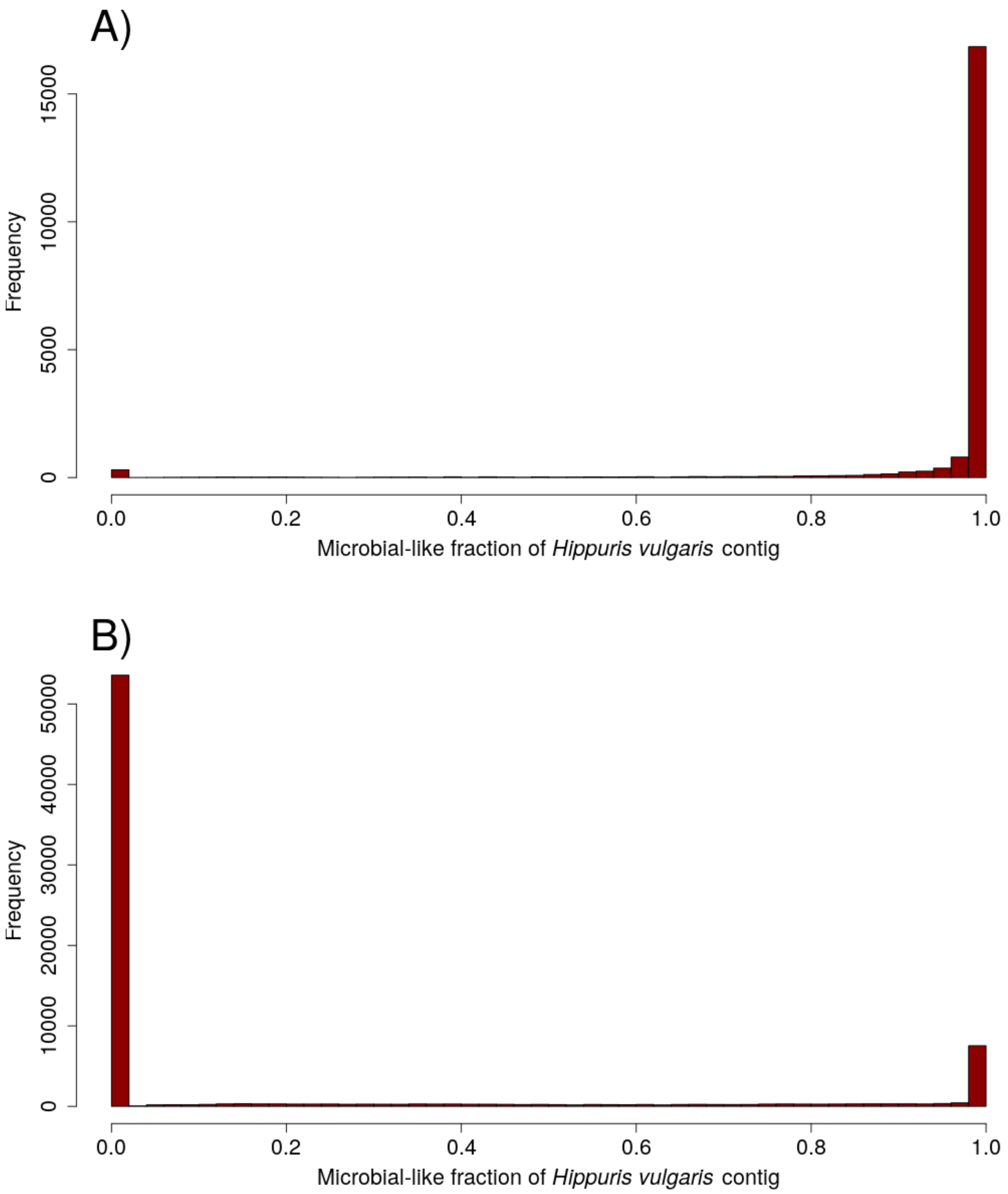

Supplementary Figure 5. Distribution of microbial-like fractions of *Hippuris vulgaris* contigs with aligned reads for: A) Arctic sample cr9_67 [28] (20,213 contigs), and B) Greenland sample 69_B2_100_L0_KapK-12-1-35 [33] (73,911 contigs).

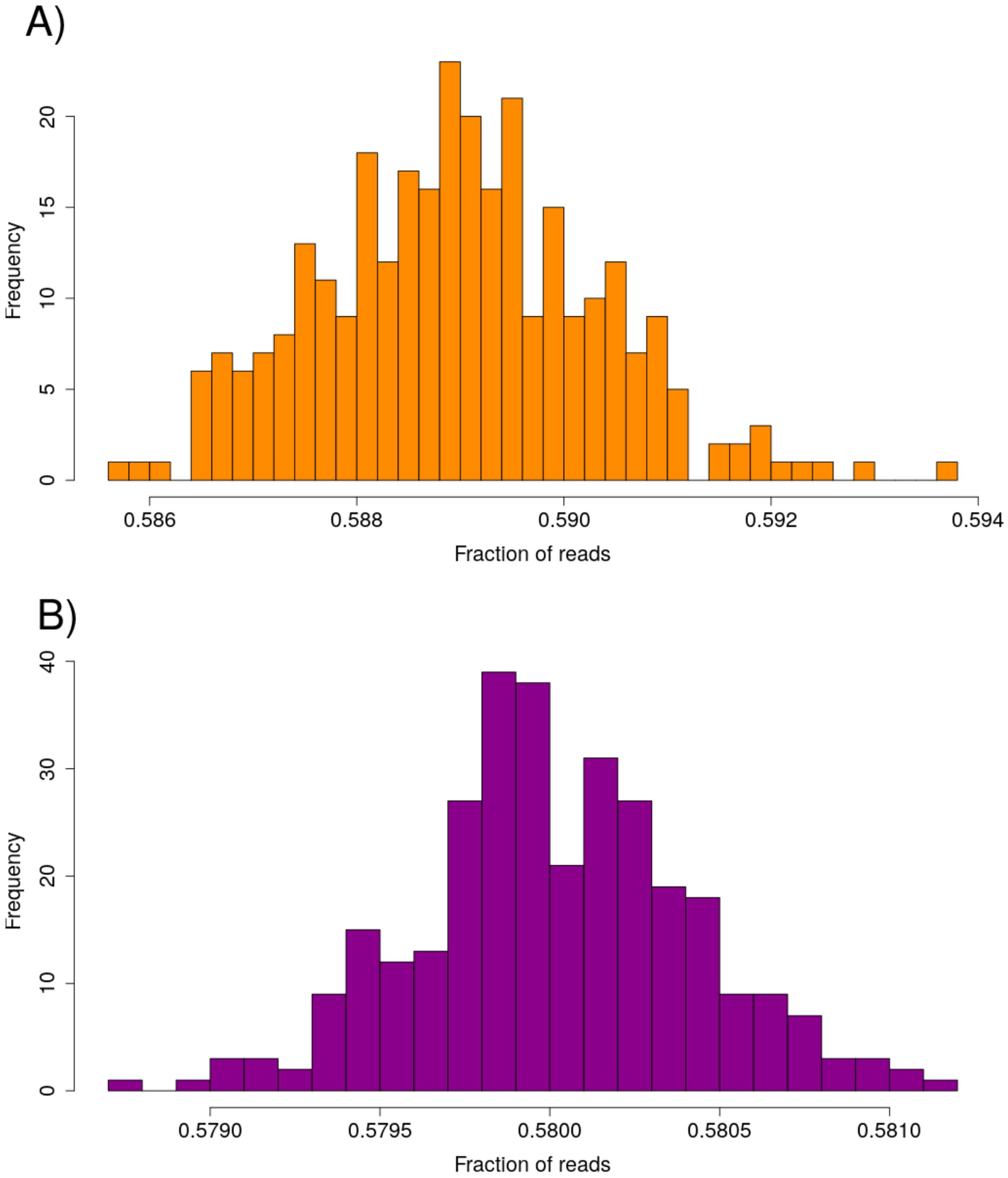

Supplementary Figure 6. Verification of *Hippuris* hit from [28] and [33]. Intersection fraction of randomly assigned reads from: A) the Arctic sample cr9_67 [28], and B) the Greenland sample 69_B2_100_L0_KapK-12-1-35 [33], with microbial-like regions in the *Hippuris vulgaris* reference genome.

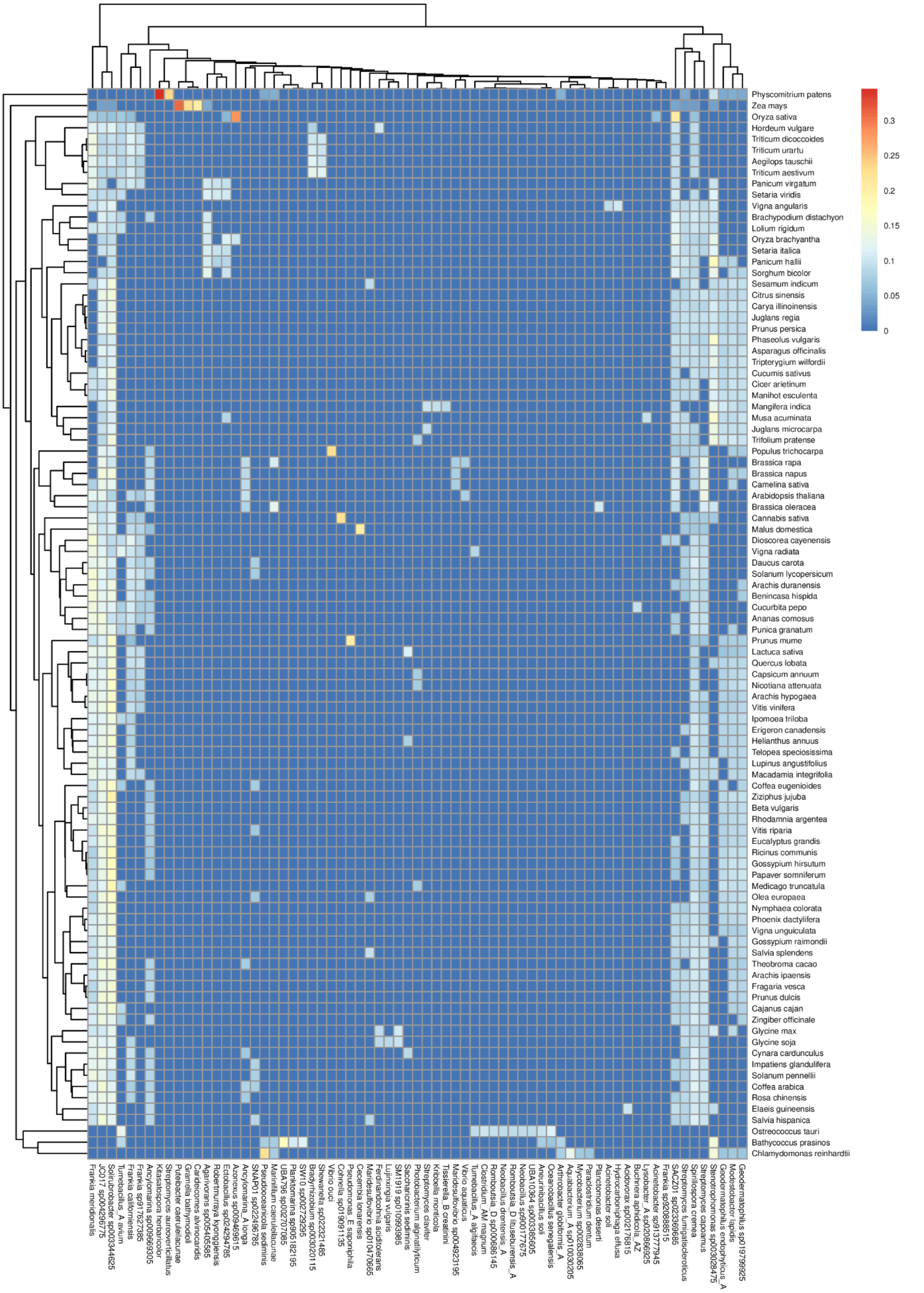

Supplementary Figure 7. Abundance heatmap of microbial-like sequences across NCBI RefSeq plants. The columns represent microbial taxa contributing to the reference genomes of plants displayed as rows. The color gradient indicates normalized abundance of microbial-like sequences (0-lowest, 1-highest).

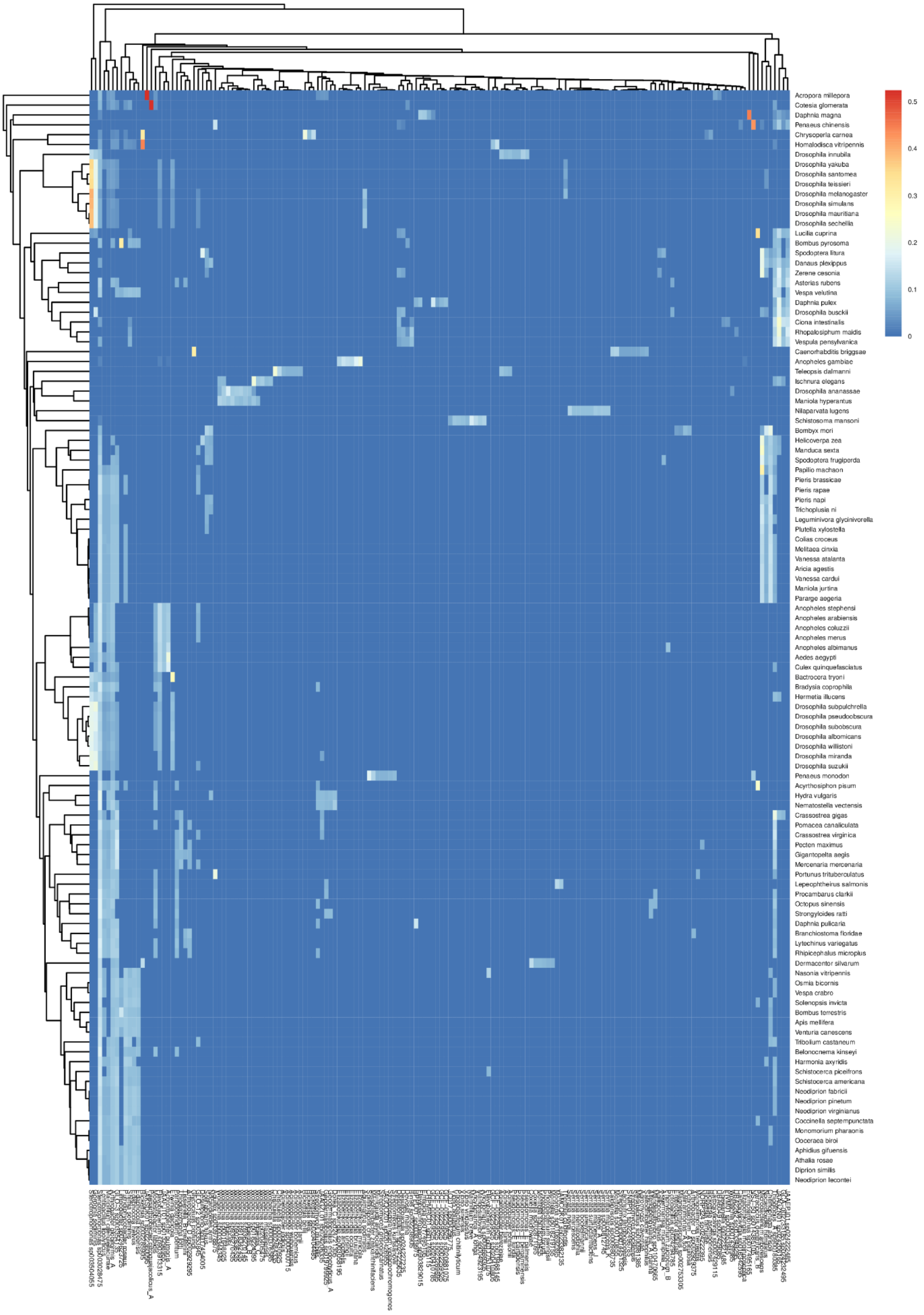

Supplementary Figure 8. Abundance heatmap of microbial-like sequences across NCBI RefSeq invertebrates. The columns represent microbial taxa contributing to the reference genomes of invertebrates displayed as rows. The color gradient indicates normalized abundance of microbial-like sequences (0-lowest, 1-highest).

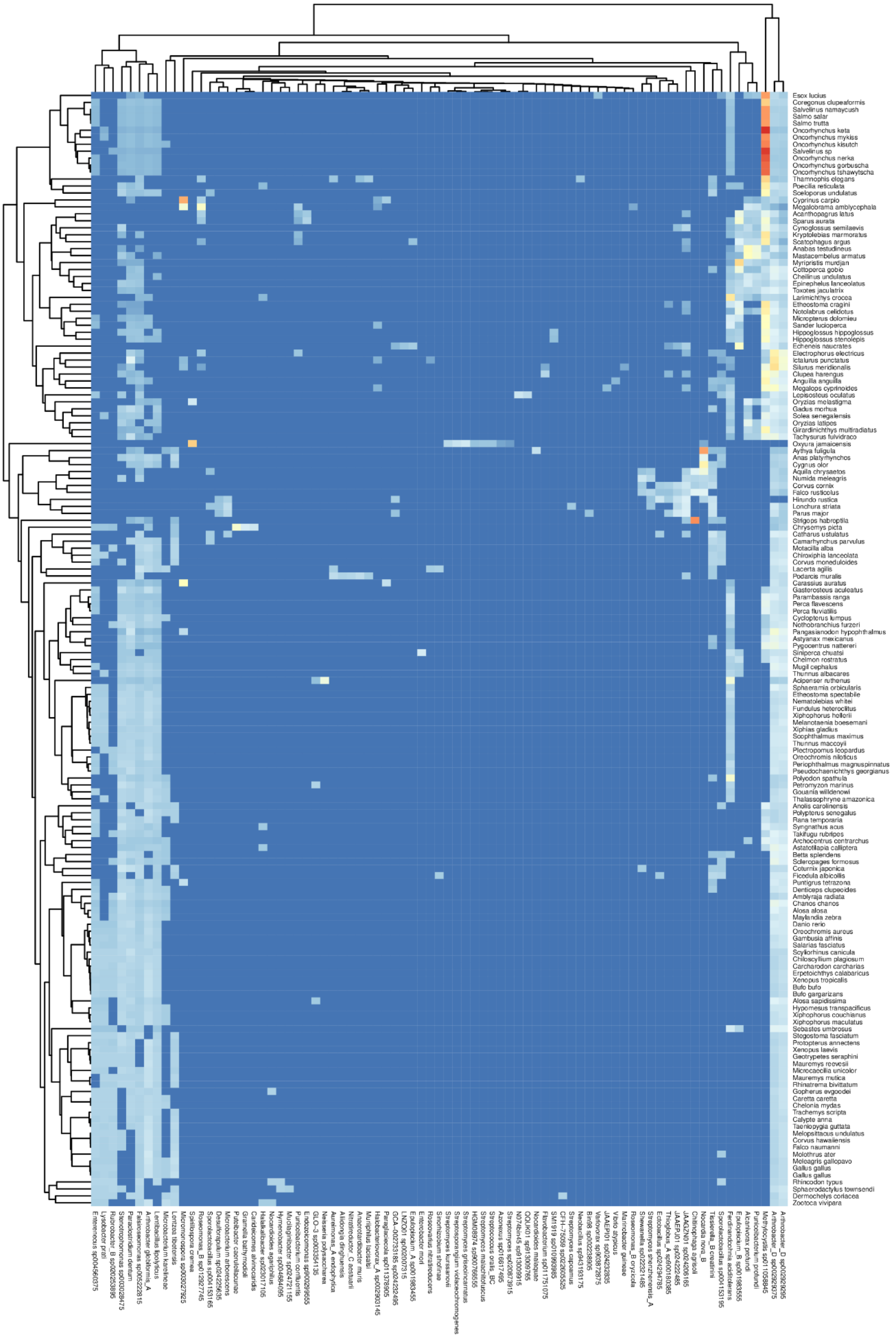

Supplementary Figure 9. Abundance heatmap of microbial-like sequences across NCBI RefSeq non-mammalian vertebrate taxa. The columns represent microbial taxa contributing to the reference genomes of vertebrates displayed as rows. The color gradient indicates normalized abundance of microbial-like sequences (0-lowest, 1-highest).

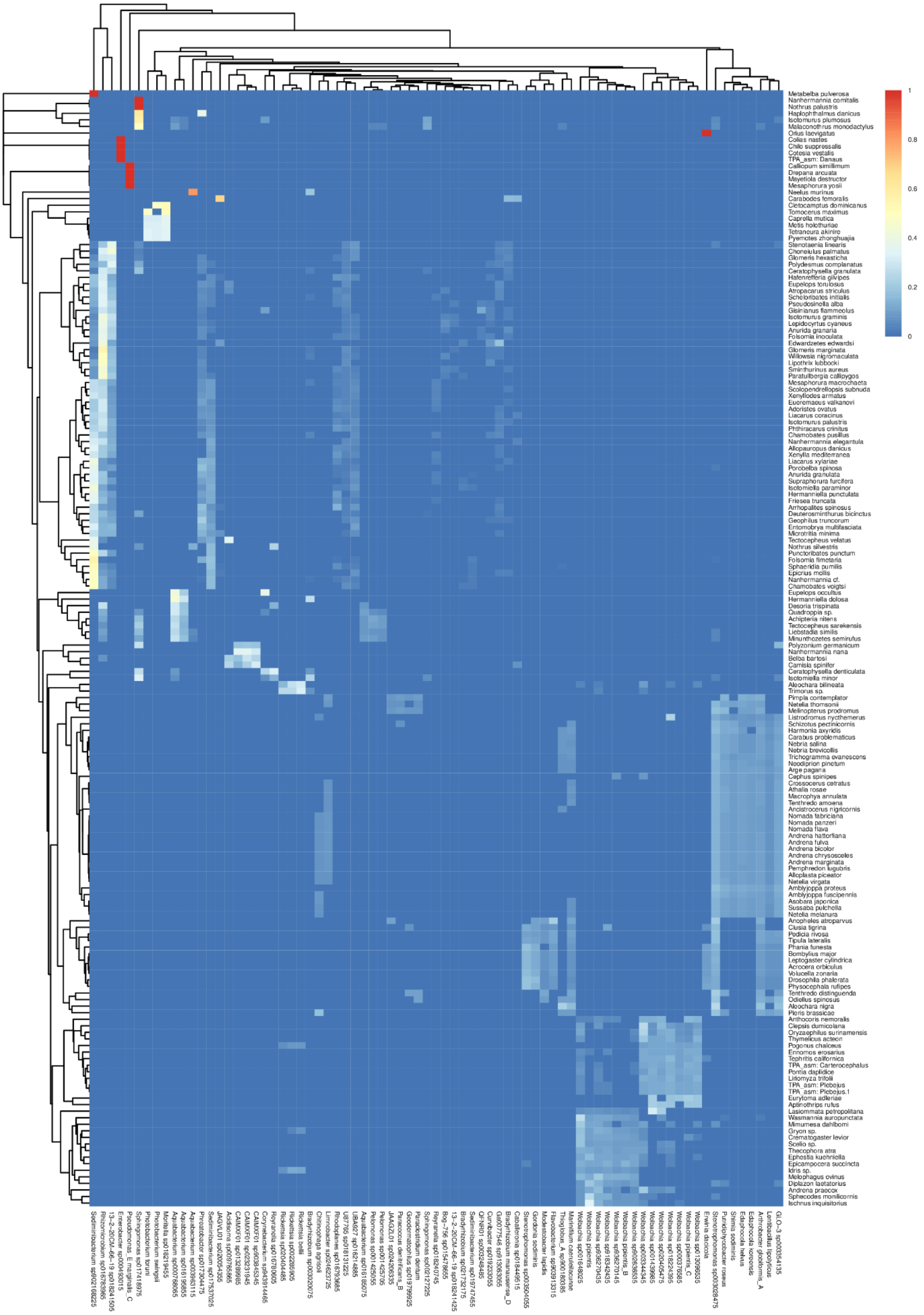

Supplementary Figure 10. Abundance heatmap of microbial-like sequences across NCBI RefSeq arthropods. The columns represent microbial taxa contributing to the reference genomes of arthropods displayed as rows. The color gradient indicates normalized abundance of microbial-like sequences (0-lowest, 1-highest).

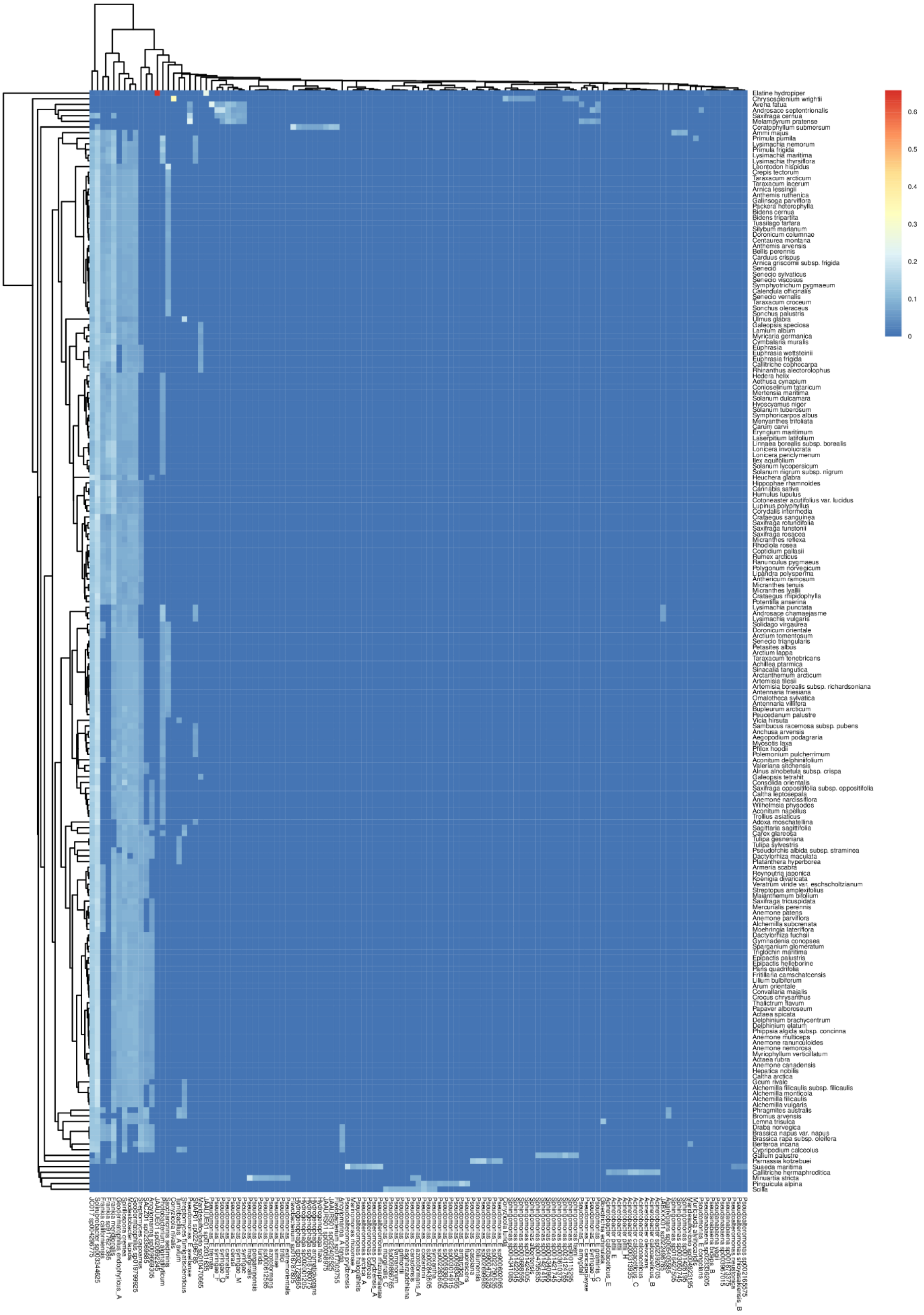

Supplementary Figure 11. Abundance heatmap of microbial-like sequences across PhyloNorway plants. The columns represent microbial taxa contributing to the reference genomes of PhyloNorway plants displayed as rows. The color gradient indicates normalized abundance of microbial-like sequences (0-lowest, 1-highest).

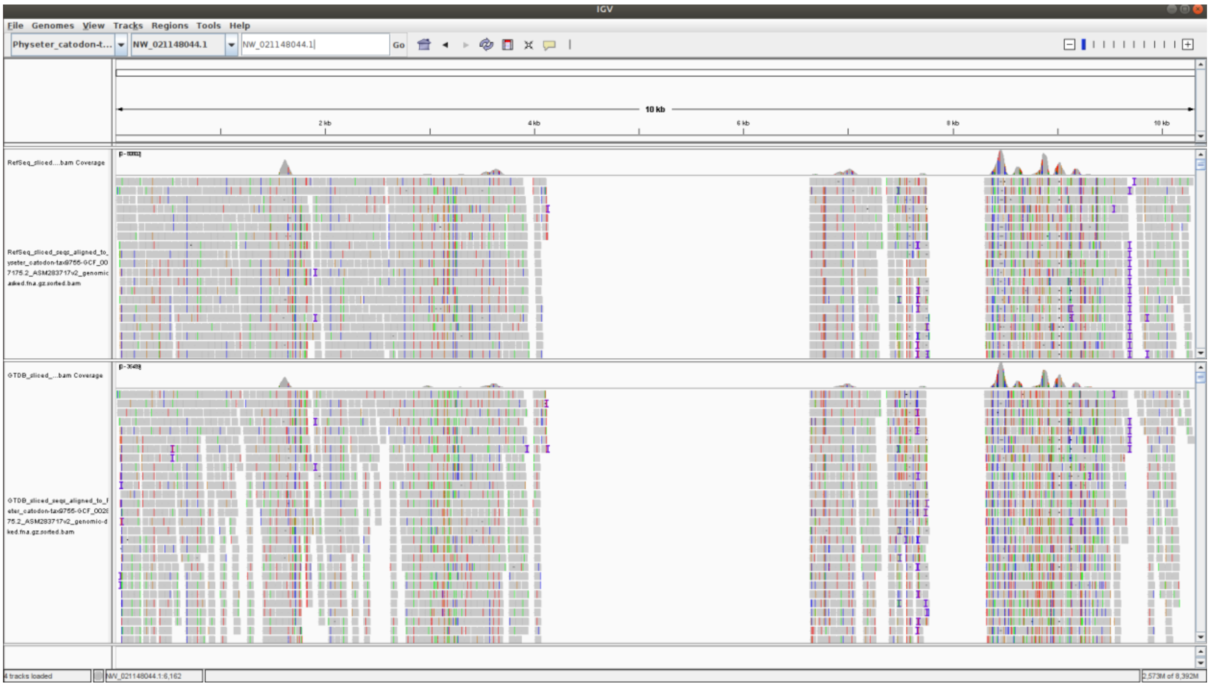

Supplementary Figure 12. Comparison of coverage of a 10 kb region of the sperm whale (*Physeter catodon*, GCA_900411695.1) reference genome by microbial pseudo-reads produced from the microbial RefSeq (top) and microbial GTDB (bottom) databases. The visualization is performed using the Integrative Genome Viewer (IGV).

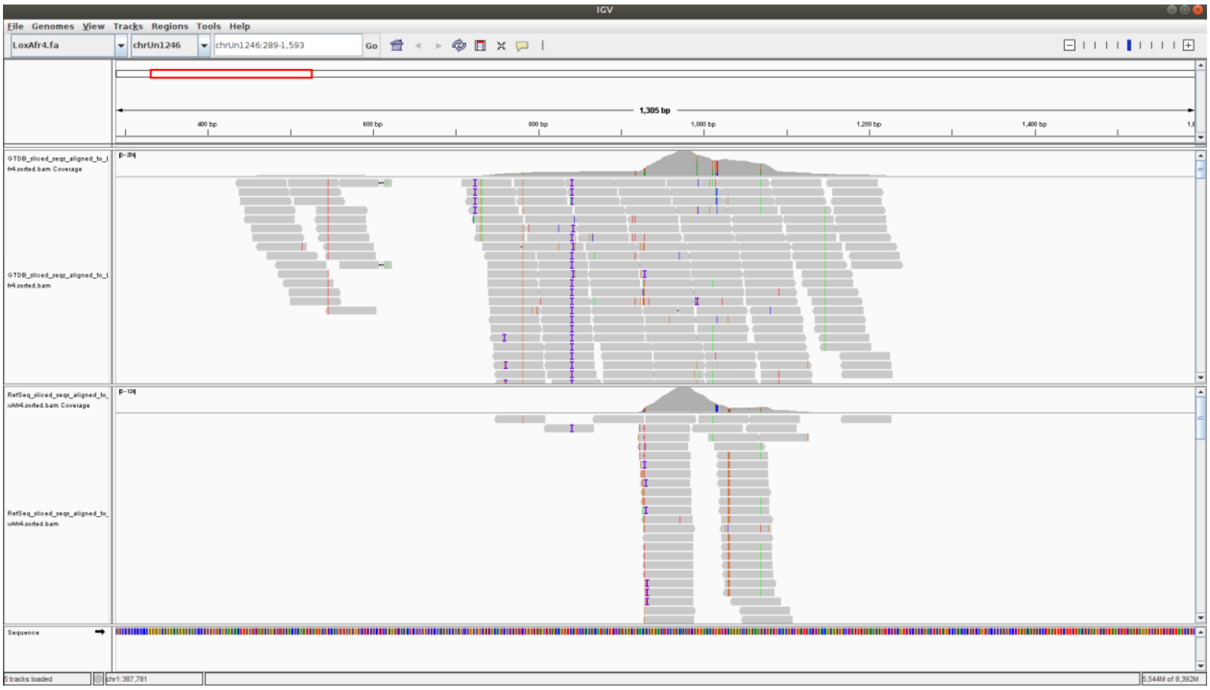

Supplementary Figure 13. Comparison of coverage of a 1.3 kb region of the African bush elephant (*Loxodonta africana*, GCF_000001905.1) reference genome by microbial pseudo-reads produced from the microbial GTDB (top) and microbial RefSeq (bottom) databases. The visualization is performed using the Integrative Genome Viewer (IGV). The visualization demonstrates that microbial GTDB pseudo-reads are capable of discovering more microbial-like regions within the eukaryotic reference genome compared to microbial RefSeq pseudo-reads.

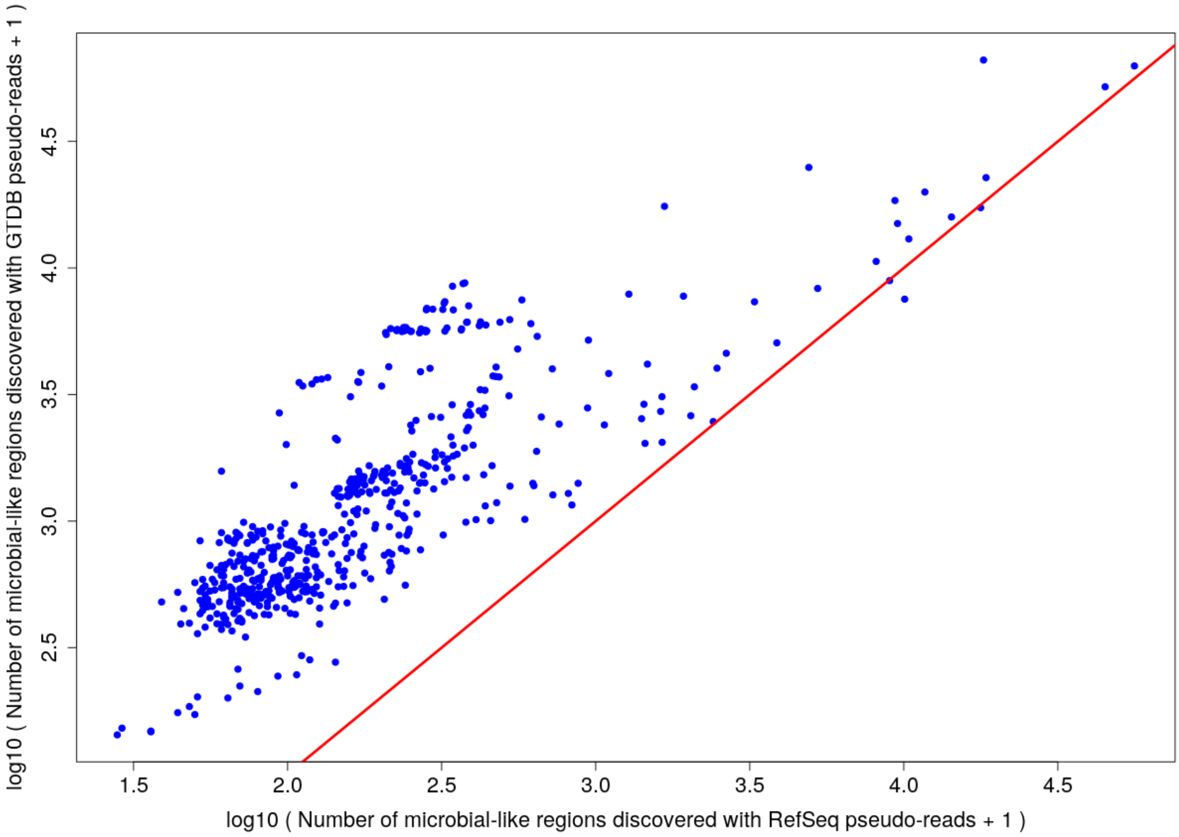

Supplementary Figure 14. Comparison of numbers of microbial-like regions in mammalian reference genomes detected by using microbial GTDB and RefSeq pseudo-reads. One point represents one mammalian reference genome. Red diagonal line highlights equal counts for RefSeq and GTDB.

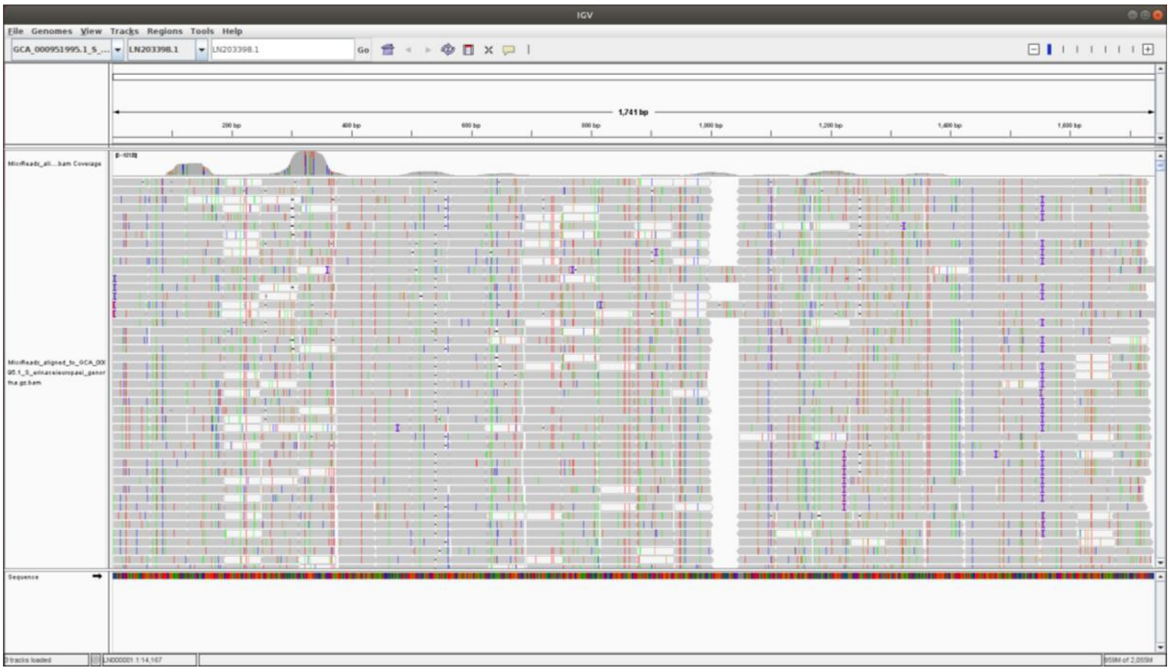

Supplementary Figure 15. Example of coverage of detected exogenous regions by mapped human pseudo-reads to the *Spirometra erinaceieuropaei* (parasitic tapeworm) reference genome GCA_000951995.1, scaffold LN203398.1 that has 100% breadth of coverage by human pseudo-reads. The visualization is performed using the Integrative Genomics Viewer (IGV).

Click here to access/download
Supplementary Material
Supplementary_Table_1_Mammals.xlsx

Click here to access/download
Supplementary Material
Supplementary_Table_2_Plants.xlsx

Click here to access/download
Supplementary Material
Supplementary_Table_3_Vertebrates.xlsx

Click here to access/download
Supplementary Material
Supplementary_Table_4_Invertebrates.xlsx

Click here to access/download
Supplementary Material
Supplementary_Table_5_Arthropods.xlsx

Click here to access/download
Supplementary Material
Supplementary_Table_6_PhyloNorway.xlsx

 Click here to access/download;Personal Cover;GigaScience_ReviewersComments_corrected.docx

GIGA-D-25-00115
Disinfecting eukaryotic reference genomes to improve taxonomic inference from ancient environmental metagenomic data, Nikolay Oskolkov; Chenyu Jin; Samantha López Clinton; Benjamin Guinet; Flore Wijnands; Ernst Johnson; Verena E. Kutschera; Cormac M. Kinsella; Peter D. Heintzman; Tom van der Valk, GigaScience

Dear Nikolay Oskolkov,

Your manuscript "Disinfecting eukaryotic reference genomes to improve taxonomic inference from ancient environmental metagenomic data" (GIGA-D-25-00115) has been assessed by our reviewers. Although it is of interest, we are unable to consider it for publication in its current form. The reviewers have raised a number of points which we believe would improve the manuscript and may allow a revised version to be published in GigaScience. Their reports, together with any other comments, are below. Please also take a moment to check our website at https://www.editorialmanager.com/giga/ for any additional comments that were saved as attachments.

- I'd like to highlight the suggestion of reviewer #1 "to develop this into a proper workflow (e.g., Snakemake), taking a set of reference fasta files and a set of contaminant fasta files as input, and running through all steps automatically." As also reviewer #2 encountered errors while running the script, implementing the method in easily deployable form will greatly improve uptake and reproducibility. Computational workflows should also be registered in workflowhub.eu and the DOIs cited in the relevant places in the manuscript.

Authors' comment: Dear Dr. Zauner, we thank you for handling our manuscript and coordinating the review process. We are thankful for the insightful comments from the reviewers, and have now revised our manuscript according to their suggestion. Among the revisions we have improved our workflow, including correcting the workflow running errors encountered by the reviewer #2. We now also provide the conda-environment with the tools used, and rewrote the workflow into a Nextflow pipeline for scalability and reproducibility. Currently, the Nextflow pipeline, as suggested by reviewer #1, accepts a set of reference fasta files and potential exogenous (contaminant) fasta-files as input, and runs through all steps automatically. In addition to having the workflow registered at the SciLifeLab Figshare portal https://doi.org/10.17044/scilifelab.28491956, we have now registered the workflow at Zenodo https://doi.org/10.5281/zenodo.16788411, as well as, as advised by GigaScience, at workflowhub.eu https://doi.org/10.48546/workflowhub.workflow.1846.1. We now provide the corresponding DOI-numbers in the "Data Availability" and "Availability of source code and requirements" sections of the manuscript.

- As your manuscript presents a method, we will consider it for the "Technical Note" section of the journal. Please have a look at our guidelines for this article type and format the manuscript accordingly (however, we are quite flexible - e.g. you can keep a section titled "discussion", if you feel it's useful.https://academic.oup.com/gigascience/pages/technical_note)

Authors' comment: Thank you for the suggestion. We have reformatted the manuscript in line with the *Technical Note* guidelines, and we would like to retain the revised *Discussion* section, as we believe it provides important context and interpretation of our results.

- In addition, please register any new software application in the bio.tools and SciCrunch.org databases to receive RRID (Research Resource Identification Initiative ID) and biotoolsID identifiers, and include these in your manuscript. These will facilitate tracking, reproducibility and re-use of your tool.

Authors' comment: We have now registered the workflow at SciCrunch.org and received the Research Resource Identification Initiative ID (RRID) SCR_027305. We also registered the workflow at bio.tools and obtained biotools:genex_workflow as the biotools unique resource ID. We included these IDs to the "Data Availability" and "Availability of source code and requirements" sections of the manuscript.

If you are able to fully address these points, we would encourage you to submit a revised manuscript to GigaScience. Once you have made the necessary corrections, please submit online at:

https://www.editorialmanager.com/giga/

If you have forgotten your username or password please use the "Send Login Details" link to get your login information. For security reasons, your password will be reset.

Please include a point-by-point within the 'Response to Reviewers' box in the submission system. Please ensure you describe additional experiments that were carried out and include a detailed rebuttal of any criticisms or requested revisions that you disagreed with. Please also ensure that your revised manuscript conforms to the journal style, which can be found in the Instructions for Authors on the journal homepage. If the data and code has been modified in the revision process please be sure to update the public versions of this too.

The due date for submitting the revised version of your article is 11 Aug 2025.

I look forward to receiving your revised manuscript soon.

Best wishes,
Hans

Dr Hans Zauner
Editor, GigaScience
www.gigasciencejournal.com

Reviewer reports:

Reviewer #1:

Summary
=================

Oskolkov et al present an approach to identify microbial contamination in reference sequence databases. They use this approach to scrutinize two previous publications in the ancient environmental DNA field, and show that some species found in these papers are likely over-estimations due to contamination in the references. They further investigate which microbial references are mostly responsible for the contamination in the examined databases, and show that the PhyloNorway references are highly contaminated.

The problem that Oskolkov et al are tackling here is highly relevant for practitioners, not just in the aeDNA field, but in whole-genome eDNA studies in general. Their findings are worrying to say the least, and indicate that future research and development is needed to fully solve the problem of contamination. This manuscript is also a cautionary tale for data sanitation, and should be read by anyone working with aeDNA.

The methodological approach is straight-forward and sound, and to my understanding makes more sense than the two existing methods that are referred to in the manuscript. Mapping the contaminants against the target (Eukaryotes) seems intuitively more reasonable than the other way round, and allows the authors to produce masks for the genomes, which is interesting and relevant in practice in order to obtain masks.

The paper is very well written and easy to follow. Furthermore, all analyses are thorough, and the figures support the claims that the authors make. All scripts and datasets are made available, and seem well documented.

In summary, the paper is of high quality, and definitely should be published. I do have some suggestions on improvements, which I list below.

Best regards,

Lucas Czech

Authors' comment: we thank the reviewer for the very valuable comments, which have helped us to substantially improve the workflow and the manuscript. Below, we provide as response in bold the reviewer's comments point-by-point.

Major
================

* One suggestion that we discussed internally after reading the preprint of the manuscript is the following: Using the presented method to map the pseudo-reads against GTDB itself, and/or against the RefSeq organelle or microbial sequences. This would serve as a further validation (everything should show up as "contaminated"), and can be used as a baseline for accuracy and other evaluations.

Authors' response: We thank the reviewer for this suggestion. We have now validated our method by aligning the GTDB pseudo-reads to >820 random GTDB reference sequences and observed the median of 99.1% of breadth of coverage. In addition, aligning the RefSeq pseudo-reads to 25 random RefSeq reference sequences resulted in median breadth of coverage of 97.2%. This confirms the expectation that GTDB and RefSeq reference sequences indeed seem to be close to fully consisting of microbial-like sequences. We have added this validation analysis to the Methods section and Supplementary Material S4 of the manuscript.

* Furthermore, would it make sense to include the human genome as a source of contamination, i.e., create pseudo-reads from it as well? The authors mention that references might be contaminated with human reads, and the workflow as presented should easily be able to accommodate for that, in order to catch that contamination.

Authors' response: Thank you very much for this excellent suggestion. Indeed, the workflow in its current state can easily accommodate the search for potential human contamination. We have pre-computed human pseudo-reads and made them publicly available via the SciLifeLab Figshre, https://doi.org/10.17044/scilifelab.28491956. We have also modified the parameters of the workflow, which now includes the option of using the pre-computed human pseudo-reads if one is interested in detecting "human-like" regions in prokaryotic or eukaryotic reference genomes. As a proof-of-concept we have run the workflow on the *Spirometra erinaceieuropaei* (parasitic tapeworm) reference genome GCA_000951995.1 which was suspected to contain human contamination in Jensen et al. Nature Communications 2019. We detected that >0.1% of the reference genome includes human-like sequences with over 50 scaffolds with length up to 1.7 kbp having 100% breadth of coverage by human pseudo-reads. The total length of all detected human-like sequences within the parasitic tapeworm reference genome is 1.4 Mbp. We present an IGV visualization of one of the fully covered scaffolds in the newly included Supplementary Figure 15. This analysis was also added to the Methods, Discussion and the new Supplementary Material S5 sections of the manuscript.

* The available scripts are great, however might make it a bit hard to use the approach in practice on other datasets or later versions. To fully leverage the method, and for practitioners to be able to use it on their own data, I suggest to develop this into a proper workflow (e.g., Snakemake), taking a set of reference fasta files and a set of contaminant fasta files as input, and running through all steps automatically. Given that the scripts already exist, this might be relatively easy to implement, and of great use for the community. I understand though if the authors feel that this is out of scope for this manuscript.

Authors' response: We agree with the reviewers suggestion. We have now made a major revision of the workflow codes and wrapped them into a Nextflow pipeline, which should ensure the reproducibility and dissemination of the method. Taking into account the previously mentioned implementation of human pseudo-reads, the pipeline follows the suggestion and is general now, i.e. it accepts a reference

genome (prokaryotic or eukaryotic) and microbial (GTDB or RefSeq) or human pseudo-reads and outputs the coordinates of predicted exogenous regions.

Minor
=================

* Line 62: "the presence of hippopotamus-like sequence" is syntactically incorrect. Either "the presence of a hippopotamus-like sequence", or "sequences" in plural would work.

Authors' response: We have now corrected this.

* Line 137, and Supplement S1: Using 60 bp pseudo-reads is yielding very specific hits, which might be what is needed here. Have the authors evaluated smaller pseudo-reads as well? As 30-ish is usually enough to identify a species, I am curious to hear the author's view on this - why 60, and not 30, 40, or 50? Wouldn't those potentially catch more contamination? Or would that be too sensitive and give too many false positives?

Authors' response: We chose a pseudo-read length of 60 bp to adopt a conservative approach in predicting microbial-like regions. While shorter pseudo-reads (e.g., 30–50 bp) could, in principle, increase the sensitivity and potentially detect more microbial-like sequences, they would also increase the risk of spurious matches due to shorter alignments and reduced sequence complexity, particularly in repetitive genomic regions. Using a length twice the conventional lower limit of ~30 bp prioritizes specificity, ensuring that predicted microbial-like sequences are robust and less likely to represent false positives. This approach minimizes the need for additional downstream validation while maintaining high confidence in our predictions. We acknowledge that this choice may miss some shorter microbial-like regions, but we consider this an acceptable trade-off to improve the reliability of the final masked reference genomes. This reasoning has now been added to the *Methods* section.

* Line 141, and Supp S1: Allowing for multi-mapping reads is certainly necessary for the reasons the authors have laid out. I am wondering however if 10 is enough here. Have the authors checked if that number is saturated for some genomes? If so, it might need increasing.

Addendum after getting to the supplement: This was indeed evaluated, great! But only on two genomes - might be good enough, but it might differ for other species, or not? I am lacking intuition here, and would like to hear the author's take on this.

Authors' response: we have indeed evaluated a range of allowed Bowtie2 multi-mappers, which we describe in Supplementary Figure 1. The main conclusion was that the number of discovered microbial-like regions was saturating after 5-10 allowed multi-mappers, and higher thresholds become exponentially computationally more expensive but provide almost no added resolution. We have however now made the number of multi-mapping pseudo-reads allowed by Bowtie2 an explicit parameter of the workflow which could improve the resolution in edge-cases (i.e. regions at extreme copy-number in the reference), please see the last argument here https://github.com/NikolayOskolkov/MCWorkflow.

* Lines 168-172: While this is the methods section, a very short description of why this PCA was computed as a means of validation, or a foreshadowing link to Fig 4, might help the reader here to understand the reason behind this here already.

Authors' response: We agree this requires additional explanation. We have now added the following sentence motivating the use of PCA for validation of our method:

"Because microbes and plants have distinct k-mer profiles, we used PCA to compare the k-mer composition of microbial-like segments identified by our method with that of

endogenous segments, aiming to confirm that these groups indeed form distinct clusters in the PCA plot"

* Lines 220ff: I needed several attempts to read and understand this sentence, and find it confusingly phrased. Might I suggest something along the lines of: "There are 81 primate genomes in the 566 assessed mammalian genomes. Yet, 37 of these are within the top 45 most contaminated genomes." Also, it might be interesting to report the range of contamination of these 37 genomes.

Authors' response: thank you for this excellent suggestion, we have now rephrased this sentence in the main text following the recommendation.

* Lines 295ff: The authors very thoroughly compute the expected percentage of overlap with regions identified as microbial-like in the Hippuris vulgaris reference. How much of that genome was reported as being microbial-like though? Maybe I've missed this, but that might be an interesting fraction to report here as well.

Authors' response: the *Hippuris vulgaris* reference genome was predicted by our method to contain 57% of microbial-like regions. This was mentioned in the manuscript, however thanks to your suggestion, we have now also emphasized it in the paragraph discussing the by-chance intersection of the mapped reads with the predicted microbial-like regions.

Reviewer #2:

Summary
=================

This manuscript addresses a significant issue in ancient metagenomics: how microbial contamination in reference genomes can influence taxonomic profiling and analytical outcomes. It is a well-written, timely, and highly relevant study. The structure is logical, the arguments are generally sound, and the results are important for the field. However, there are several major limitations that must be addressed to strengthen the study.

I believe this work could be of interest to the readers of GigaScience, but the current analysis and documentation require substantial improvement.

Authors' comment: we thank the reviewer for the very valuable comments, they have helped us to substantially improve the workflow and the manuscript. Below, we address in bold the reviewer's comments point-by-point.

Major Comments
=================

1. Handling of conserved and closely related genes/genomes

It is unclear how the workflow accounts for photosynthetic or ancestrally derived genomic regions in plants, which may be legitimate components of plant genomes. Classifying such regions as microbial contamination may not be appropriate, and masking them in downstream analyses could distort results. For example, GTDB includes over 5,000 cyanobacterial genomes, some of which are among the closest known relatives to plants. Given the potential for genuine sequence similarity, particularly under permissive mapping parameters, the

reported contamination may be overestimated. This concern is also relevant to conclusions drawn from single-sample tests.

Authors' response: thank you, we agree this is an important question to discuss further. The GTDB version r214, that was used in this study, contains 3,846 reference genomes (out of the total 394,932 genomes) belonging to the *Cyanobacteriota* phylum, representing only ~1% of the total number of reference sequences in GTDB. We now tested the enrichment of cyanobacterial pseudo-reads in the predicted microbial-like regions of *Hippuris vulgaris* and *Claytonia eschscholtzii* plants from the PhyloNorway dataset, which were predicted to contain the highest percentage of microbial-like regions, 57% and 70%, respectively. Based on the annotation from the GTDB r214, there were 622,336,764 cyanobacterial pseudo-reads out of total 26,089,195,106 pseudo-reads, i.e. 2.4%, pre-computed from the GTDB reference sequences. However, we observed only 2,739,015 cyanobacterial pseudo-reads of of total 481,618,468 (i.e. 0.6%) aligned to the *Hippuris vulgaris*, and only 1,688,444 cyanobacterial pseudo-reads out of 408,824,087 (i.e. 0.4%) aligned to *Claytonia eschscholtzii*, reference genomes from the PhyloNorway dataset. This implies that cyanobacterial pseudo-reads do not align more often to the two PhyloNorway plant references than would be expected by chance. Therefore, although potential over-masking of plant references following our method is plausible, it is not supported by our analysis of GTDB data. Nevertheless, because of ambiguity of the analysis, we cannot fully reject the hypothesis that due to the genuine sequence similarity between cyanobacterial and plant genomes, the fractions of microbial-like regions in some plant reference genomes predicted in this study may be overestimated. However, if such over-masking occurs and these regions represent genuine host genome sequences, microbial sequences in aeDNA samples can still align to them, potentially leading to erroneous taxonomic assignments. In this context, masking these regions remains beneficial, as it promotes a more conservative approach and thus a more reliable detection of true species present in ancient samples.

We have now added these results to the Discussion section of the revised manuscript.

2. Contamination in GTDB Database

The manuscript does not address possible contamination within the GTDB database itself, which appears to be used uncritically for cleaning eukaryotic reference genomes. Prior work has raised concerns about microbial genome contamination (e.g., https://pubmed.ncbi.nlm.nih.gov/38809778/). At a minimum, this limitation should be discussed; ideally, it should be assessed computationally. For instance, pseudo-reads generated from a well-annotated genome (e.g., human) could be mapped against microbial references to demonstrate the complexity and potential pitfalls in cross-superkingdom contamination detection.

Alternatively, I encourage the authors to develop or include a method to evaluate the specificity and sensitivity of the contaminated regions identified in the eukaryotic genomes. This would reduce reliance on external assumptions and provide stronger validation for their conclusions.

Authors' response: thank you, we agree this is an important consideration. We indeed assume GTDB to represent a microbial "ground-truth" and free from eukaryotic contamination as it is a highly curated database. We aimed to further validate this assumption by the additional analysis that we performed by aligning human pseudo-reads (prepared in the same way as the microbial pseudo-reads described in the manuscript) to >820 random GTDB and 25 RefSeq microbial reference sequences. In the whole experiment we observed only 3 human reads aligned to any microbial reference which represents a negligible breadth of coverage with median 0% and ensures that the microbial pseudo-reads used in this manuscript are very likely free from any substantial eukaryotic contamination.

In contrast, when we screen eukaryotic reference genomes for the presence of human-like sequences, we discover substantially higher coverage. For example, *Spirometra erinaceieuropaei* (parasitic tapeworm) reference genome GCA_000951995.1, indicated over 8 million aligned human pseudo-reads covering >0.1% of the reference genome with the total length of human-like sequences of 1.4 Mbp. We present an IGV visualization of one of 100% covered scaffolds of the parasitic tapeworm reference in the newly

included Supplementary Figure 15. In addition, screening the reference genome GCF_002220235.1 of *Bathycoccus prasinos* (green algae), we detected over 236,000 aligned human pseudo-reads covering ~0.2% of the reference with the total length of the potentially exogenous regions of 37 kbp.

To further address the sensitivity and specificity of our method, we ran it using a random subset of 6.5 million GTDB pseudo-reads and the screened reference genome represented concatenated hg38 human reference genome with 16 microbial reference genomes (corresponding to 726 reference sequences on chromosome and scaffold level) used in Pochon at al. Genome Biology 2023. We observed only 1 microbial pseudo-read mapped to one chromosome (chr 12) out of 24 canonical and 432 decoy chromosomes in the human hg38 reference genome, while 202,762 pseudo-reads were mapped to the microbial reference sequences. This demonstrates the high specificity of our method as well as the very low risk of Bowtie2 non-specific alignments (please see the next reviewer's comment regarding the risk of including non-specific alignments).

We have now added this analysis to the Methods, Discussion as well as Supplementary Material S5 sections of the revised manuscript.

3. Quantitative Analysis of Sequence Similarity

The manuscript lacks a detailed analysis of the similarity between microbial reads and the eukaryotic regions they map to. Given that the Bowtie2 setting --very-sensitive allows alignments with substantial mismatches (e.g., 60 bp reads with >10 bp differences), there is a risk of including non-specific alignments. A quantitative analysis of sequence identity across these alignments would be helpful, along with a justified similarity threshold to exclude highly divergent mappings that may not be of microbial origin.

Authors' response: thank you for this very valid point. Bowtie2 has a special non-trivial scoring system to determine whether a read will be mapped or not. The scoring system is not solely based on the exact number of mismatches but includes multiple other metrics such as base quality, gaps, clipping etc. Nevertheless, Bowtie2 prioritizes high-scoring (i.e. more similar) alignments, and heavily penalizes divergence. Empirically, we can see that a 60 bp read with more than ~6 mismatches (average nucleotide identity ANI=90%) will usually fail to align, even under --very-sensitive –end-to-end. For example, tested on RefSeq plants and PhyloNorway references with aligned GTDB pseudo-reads, we observe on average 4 ± 0.4 and 4 ± 0.2 mismatches per read, respectively, i.e. the similarity of ANI=93%. The comment that "Bowtie2 setting --very-sensitive allows alignments with substantial mismatches (e.g., 60 bp reads with >10 bp differences)" is not directly supported by our empirical testing. Taking into account that a typical ANI threshold for ancient metagenomics projects is set much lower than 93%, i.e. often down to 85% (Pochon et al. 2023, Huebler et al. 2019), due to DNA damage, we assume the risk of non-specific alignments with the Bowtie2 mapping parameters used in this study is low.

Also (please see the answer to the previous question), in the experiment when we aligned human pseudo-reads to microbial references and vice-versa microbial pseudo-reads to concatenated human + microbial references we discovered extremely few (3 and 1 respectively) miss-mapped reads, which highlights the high specificity of Bowtie2 alignments with –very-sensitive and –end-to-end arguments used in this study.

We have now added this additional analysis of sequence similarity threshold to the Supplementary Material S1 section.

4. Reproducibility and Workflow Usability

The computational workflow should be included in the GitHub repository to ensure reproducibility. I attempted to run the small test example provided, but encountered multiple issues on both a Unix-based laptop and a

Linux server. These included absolute paths in scripts, unresolved dependencies, and failures in execution. I recommend that the authors:

- Fix hardcoded paths and ensure portability across environments
- Provide a working bash script that runs successfully on clean systems
- Include a conda environment file or, at a minimum, document tool versions and dependencies

Here is a sample of the errors encountered:

```
xxxxxx@xxxxxxxxx MCWorkflow % ./micr_cont_detect.sh GCF_002220235.fna.gz data GTDB 4
GTDB_sliced_seqs_sliding_window.fna.gz GTDB_fna2name.txt

PREPARING FILES FOR ANALYSIS OF GCF_002220235.fna.gz REFERENCE GENOME
mkdir: GCF_002220235.fna.gz_GTDB: File exists

BUILDING BOWTIE2 INDEX FOR GCF_002220235.fna.gz REFERENCE GENOME
ALIGNING MICROBIAL READS WITH BOWTIE2 TO GCF_002220235.fna.gz REFERENCE GENOME
(ERR): "data/GCF_002220235.fna.gz" does not exist or is not a Bowtie 2 index
Exiting now ...
[main_samview] fail to read the header from "-".
[W::hts_set_opt] Cannot change block size for this format
samtools sort: failed to read header from "-"
samtools index: "MicrReads_aligned_to_GCF_002220235.fna.gz.bam" is in a format that cannot be usefully
indexed
RANKING GCF_002220235.fna.gz CONTIGS BY NUMBER OF MAPPED MICROBIAL READS
[main_samview] fail to read the header from "MicrReads_aligned_to_GCF_002220235.fna.gz.bam".
sed: 1: "contigs_abund_sorted_GT ...": command c expects \ followed by text
sed: 1: "contigs_abund_sorted_GT ...": command c expects \ followed by text
COMPUTING BREADTH OF COVERAGE FOR EACH CONTIG AND COORDINATES OF
MICROBIAL CONTAMINATION FOR GCF_002220235.fna.gz REFERENCE GENOME

AGGREGATING RESULTS FOR GCF_002220235.fna.gz REFERENCE GENOME AND CLEANING
paste: total_length_per_ref.txt: No such file or directory
sed: 1: "contigs_boc_sorted_GTDB ...": command c expects \ followed by text
sed: 1: "contigs_boc_sorted_GTDB ...": command c expects \ followed by text
rm: total_length_per_ref.txt: No such file or directory
rm: boc_per_ref.txt: No such file or directory
COMPUTING LIST OF MOST ABUNDANT MICROBES CONTMAMINATING GCF_002220235.fna.gz
REFERENCE GENOME
[main_samview] fail to read the header from "MicrReads_aligned_to_GCF_002220235.fna.gz.bam".

ANALYSIS FOR GCF_002220235.fna.gz REFERENCE GENOME FINISHED SUCCESSFULLY
xxxxxx@xxxxxxxxx MCWorkflow % ll data
total 103232
-rwxr-xr-x 1 xxxxxx staff 4941250 May 9 11:35 GCF_002220235.fna.gz
-rw-r--r-- 1 xxxxxx staff 13403961 May 9 11:50 GCF_002220235.fna.gz.1.bt2l
-rw-r--r-- 1 xxxxxx staff 7519068 May 9 11:50 GCF_002220235.fna.gz.2.bt2l
-rw-r--r-- 1 xxxxxx staff 709 May 9 11:50 GCF_002220235.fna.gz.3.bt2l
-rw-r--r-- 1 xxxxxx staff 3759531 May 9 11:50 GCF_002220235.fna.gz.4.bt2l
-rw-r--r-- 1 xxxxxx staff 13403961 May 9 11:50 GCF_002220235.fna.gz.rev.1.bt2l
-rw-r--r-- 1 xxxxxx staff 7519068 May 9 11:50 GCF_002220235.fna.gz.rev.2.bt2l
drwxr-xr-x 6 xxxxxx staff 192 May 9 11:50 GCF_002220235.fna.gz_GTDB
-rw-r--r-- 1 xxxxxx staff 67376 May 9 11:50 bowtie2-build.log
```

Authors' response: thank you very much for testing our workflow and your suggestions! We agree and we have now substantially improved the reproducibility and usability of the workflow. Specifically, first, we resolved the hard-coded paths issue which now makes it possible to run the workflow from the cloned repository without manually editing the paths. Second, we added the conda environment file, that assists installing the workflow tools, and corresponding clarifications to the README in the github repository page. Finally, we wrapped the workflow up via the Nextflow framework for further scalability and reproducibility. The workflow has been tested on one laptop, one stationary workstation and two computer clusters where it ran without errors. We invite the reviewer to test the workflow again following the command lines presented here:

https://github.com/NikolayOskolkov/MCWorkflow?tab=readme-ov-file#quick-start.
