# Supplementary material for: Improving taxonomic inference from ancient environmental metagenomes by masking microbial-like regions in reference genomes
Source: Gigascience. 2025 Oct 3;14:giaf108. doi: 10.1093/gigascience/giaf108 (PMC12491943; doi:10.1093/gigascience/giaf108)
Supplement: giaf108_Authors_Response_To_Reviewer_Comments_original_submission [file giaf108_authors_response_to_reviewer_comments_original_submission.pdf]

Author's Response To Reviewer Comments

Dear Nikolay Oskolkov,

Your manuscript "Disinfecting eukaryotic reference genomes to improve taxonomic inference from ancient environmental metagenomic data" (GIGA-D-25-00115) has been assessed by our reviewers. Although it is of interest, we are unable to consider it for publication in its current form. The reviewers have raised a number of points which we believe would improve the manuscript and may allow a revised version to be published in GigaScience. Their reports, together with any other comments, are below. Please also take a moment to check our website at https://www.editorialmanager.com/giga/ for any additional comments that were saved as attachments.

- I'd like to highlight the suggestion of reviewer #1 "to develop this into a proper workflow (e.g., Snakemake), taking a set of reference fasta files and a set of contaminant fasta files as input, and running through all steps automatically." As also reviewer #2 encountered errors while running the script, implementing the method in easily deployable form will greatly improve uptake and reproducibility. Computational workflows should also be registered in workflowhub.eu and the DOIs cited in the relevant places in the manuscript.

Authors' comment: Dear Dr. Zauner, we thank you for handling our manuscript and coordinating the review process. We are thankful for the insightful comments from the reviewers, and have now revised our manuscript according to their suggestion. Among the revisions we have improved our workflow, including correcting the workflow running errors encountered by the reviewer #2. We now also provide the conda-environment with the tools used, and rewrote the workflow into a Nextflow pipeline for scalability and reproducibility. Currently, the Nextflow pipeline, as suggested by reviewer #1, accepts a set of reference fasta files and potential exogenous (contaminant) fasta-files as input, and runs through all steps automatically. In addition to having the workflow registered at the SciLifeLab Figshare portal https://doi.org/10.17044/scilifelab.28491956, we have now registered the workflow at Zenodo https://doi.org/10.5281/zenodo.16788411, as well as, as advised by GigaScience, at workflowhub.eu https://doi.org/10.48546/workflowhub.workflow.1846.1. We now provide the corresponding DOI-numbers in the "Data Availability" and "Availability of source code and requirements" sections of the manuscript.

- As your manuscript presents a method, we will consider it for the "Technical Note" section of the journal. Please have a look at our guidelines for this article type and format the manuscript accordingly (however, we are quite flexible - e.g. you can keep a section titled "discussion", if you feel it's useful.https://academic.oup.com/gigascience/pages/technical_note)

Authors' comment: Thank you for the suggestion. We have reformatted the manuscript in line with the Technical Note guidelines, and we would like to retain the revised Discussion section, as we believe it provides important context and interpretation of our results.

- In addition, please register any new software application in the bio.tools and SciCrunch.org databases to receive RRID (Research Resource Identification Initiative ID) and biotoolsID identifiers, and include these in your manuscript. These will facilitate tracking, reproducibility and re-use of your tool.

Authors' comment: We have now registered the workflow at SciCrunch.org and received the Research Resource Identification Initiative ID (RRID) SCR_027305. We also registered the workflow at bio.tools and obtained biotools:genex_workflow as the biotools unique resource ID. We included these IDs to the "Data Availability" and "Availability of source code and requirements" sections of the manuscript.

If you are able to fully address these points, we would encourage you to submit a revised manuscript to GigaScience. Once you have made the necessary corrections, please submit online at:

https://www.editorialmanager.com/giga/

If you have forgotten your username or password please use the "Send Login Details" link to get your login information. For security reasons, your password will be reset.

Please include a point-by-point within the 'Response to Reviewers' box in the submission system. Please ensure you describe additional experiments that were carried out and include a detailed rebuttal of any criticisms or requested revisions that you disagreed with. Please also ensure that your revised manuscript conforms to the journal style, which can be found in the Instructions for Authors on the journal homepage. If the data and code has been modified in the revision process please be sure to update the public versions of this too.

The due date for submitting the revised version of your article is 11 Aug 2025.

I look forward to receiving your revised manuscript soon.

Best wishes,
Hans

Dr Hans Zauner
Editor, GigaScience
www.gigasciencejournal.com

Reviewer reports:

Reviewer #1:

Summary
=================

Oskolkov et al present an approach to identify microbial contamination in reference sequence databases. They use this approach to scrutinize two previous publications in the ancient environmental DNA field, and show that some species found in these papers are likely over-estimations due to contamination in the references. They further investigate which microbial references are mostly responsible for the contamination in the examined databases, and show that the PhyloNorway references are highly contaminated.

The problem that Oskolkov et al are tackling here is highly relevant for practitioners, not just in the aeDNA field, but in whole-genome eDNA studies in general. Their findings are worrying to say the least, and indicate that future research and development is needed to fully solve the problem of contamination. This manuscript is also a cautionary tale for data sanitation, and should be read by anyone working with aeDNA.

The methodological approach is straight-forward and sound, and to my understanding makes more sense than the two existing methods that are referred to in the manuscript. Mapping the contaminants against the target (Eukaryotes) seems intuitively more reasonable than the other way round, and allows the authors to produce masks for the genomes, which is interesting and relevant in practice in order to obtain masks.

The paper is very well written and easy to follow. Furthermore, all analyses are thorough, and the figures support the claims that the authors make. All scripts and datasets are made available, and seem well documented.

In summary, the paper is of high quality, and definitely should be published. I do have some suggestions on improvements, which I list below.

Best regards,
Lucas Czech

Authors' comment: we thank the reviewer for the very valuable comments, which have helped us to substantially improve the workflow and the manuscript. Below, we provide as response in bold the reviewer's comments point-by-point.

Major
=================

* One suggestion that we discussed internally after reading the preprint of the manuscript is the following: Using the presented method to map the pseudo-reads against GTDB itself, and/or against the RefSeq organelle or microbial sequences. This would serve as a further validation (everything should show up as "contaminated"), and can be used as a baseline for accuracy and other evaluations.

Authors' response: We thank the reviewer for this suggestion. We have now validated our method by aligning the GTDB pseudo-reads to >820 random GTDB reference sequences and observed the median of 99.1% of breadth of coverage. In addition, aligning the RefSeq pseudo-reads to 25 random RefSeq reference sequences resulted in median breadth of coverage of 97.2%. This confirms the expectation that GTDB and RefSeq reference sequences indeed seem to be close to fully consisting of microbial-like sequences. We have added this validation analysis to the Methods section and Supplementary Material S4 of the manuscript.

* Furthermore, would it make sense to include the human genome as a source of contamination, i.e., create pseudo-reads from it as well? The authors mention that references might be contaminated with human reads, and the workflow as presented should easily be able to accommodate for that, in order to catch that contamination.

Authors' response: Thank you very much for this excellent suggestion. Indeed, the workflow in its current state can easily accommodate the search for potential human contamination. We have pre-computed human pseudo-reads and made them publicly available via the SciLifeLab Figshre, https://doi.org/10.17044/scilifelab.28491956. We have also modified the parameters of the workflow, which now includes the option of using the pre-computed human pseudo-reads if one is interested in detecting "human-like" regions in prokaryotic or eukaryotic reference genomes. As a proof-of-concept we have run the workflow on the Spirometra erinaceieuropaei (parasitic tapeworm) reference genome GCA_000951995.1 which was suspected to contain human contamination in Jensen et al. Nature Communications 2019. We detected that >0.1% of the reference genome includes human-like sequences with over 50 scaffolds with length up to 1.7 kbp having 100% breadth of coverage by human pseudo-reads. The total length of all detected human-like sequences within the parasitic tapeworm reference genome is 1.4 Mbp. We present an IGV visualization of one of the fully covered scaffolds in the newly included Supplementary Figure 15. This analysis was also added to the Methods, Discussion and the new Supplementary Material S5 sections of the manuscript.

* The available scripts are great, however might make it a bit hard to use the approach in practice on other datasets or later versions. To fully leverage the method, and for practitioners to be able to use it on their own data, I suggest to develop this into a proper workflow (e.g., Snakemake), taking a set of reference fasta files and a set of contaminant fasta files as input, and running through all steps automatically. Given that the scripts already exist, this might be relatively easy to implement, and of great use for the community. I understand though if the authors feel that this is out of scope for this manuscript.

Authors' response: We agree with the reviewers suggestion. We have now made a major revision of the workflow codes and wrapped them into a Nextflow pipeline, which should ensure the reproducibility and dissemination of the method. Taking into account the previously mentioned implementation of human pseudo-reads, the pipeline follows the suggestion and is general now, i.e. it accepts a reference genome

(prokaryotic or eukaryotic) and microbial (GTDB or RefSeq) or human pseudo-reads and outputs the coordinates of predicted exogenous regions.

Minor
=================

* Line 62: "the presence of hippopotamus-like sequence" is syntactically incorrect. Either "the presence of a hippopotamus-like sequence", or "sequences" in plural would work.

Authors' response: We have now corrected this.

* Line 137, and Supplement S1: Using 60 bp pseudo-reads is yielding very specific hits, which might be what is needed here. Have the authors evaluated smaller pseudo-reads as well? As 30-ish is usually enough to identify a species, I am curious to hear the author's view on this - why 60, and not 30, 40, or 50? Wouldn't those potentially catch more contamination? Or would that be too sensitive and give too many false positives?

Authors' response: We chose a pseudo-read length of 60 bp to adopt a conservative approach in predicting microbial-like regions. While shorter pseudo-reads (e.g., 30–50 bp) could, in principle, increase the sensitivity and potentially detect more microbial-like sequences, they would also increase the risk of spurious matches due to shorter alignments and reduced sequence complexity, particularly in repetitive genomic regions. Using a length twice the conventional lower limit of ~30 bp prioritizes specificity, ensuring that predicted microbial-like sequences are robust and less likely to represent false positives. This approach minimizes the need for additional downstream validation while maintaining high confidence in our predictions. We acknowledge that this choice may miss some shorter microbial-like regions, but we consider this an acceptable trade-off to improve the reliability of the final masked reference genomes. This reasoning has now been added to the Methods section.

* Line 141, and Supp S1: Allowing for multi-mapping reads is certainly necessary for the reasons the authors have laid out. I am wondering however if 10 is enough here. Have the authors checked if that number is saturated for some genomes? If so, it might need increasing.

Addendum after getting to the supplement: This was indeed evaluated, great! But only on two genomes - might be good enough, but it might differ for other species, or not? I am lacking intuition here, and would like to hear the author's take on this.

Authors' response: we have indeed evaluated a range of allowed Bowtie2 multi-mappers, which we describe in Supplementary Figure 1. The main conclusion was that the number of discovered microbial-like regions was saturating after 5-10 allowed multi-mappers, and higher thresholds become exponentially computationally more expensive but provide almost no added resolution. We have however now made the number of multi-mapping pseudo-reads allowed by Bowtie2 an explicit parameter of the workflow which could improve the resolution in edge-cases (i.e. regions at extreme copy-number in the reference), please see the last argument here https://github.com/NikolayOskolkov/MCWorkflow.

* Lines 168-172: While this is the methods section, a very short description of why this PCA was computed as a means of validation, or a foreshadowing link to Fig 4, might help the reader here to understand the reason behind this here already.

Authors' response: We agree this requires additional explanation. We have now added the following sentence motivating the use of PCA for validation of our method:

"Because microbes and plants have distinct k-mer profiles, we used PCA to compare the k-mer composition of microbial-like segments identified by our method with that of endogenous segments, aiming to confirm

that these groups indeed form distinct clusters in the PCA plot"

* Lines 220ff: I needed several attempts to read and understand this sentence, and find it confusingly phrased. Might I suggest something along the lines of: "There are 81 primate genomes in the 566 assessed mammalian genomes. Yet, 37 of these are within the top 45 most contaminated genomes." Also, it might be interesting to report the range of contamination of these 37 genomes.

Authors' response: thank you for this excellent suggestion, we have now rephrased this sentence in the main text following the recommendation.

* Lines 295ff: The authors very thoroughly compute the expected percentage of overlap with regions identified as microbial-like in the Hippuris vulgaris reference. How much of that genome was reported as being microbial-like though? Maybe I've missed this, but that might be an interesting fraction to report here as well.

Authors' response: the Hippuris vulgaris reference genome was predicted by our method to contain 57% of microbial-like regions. This was mentioned in the manuscript, however thanks to your suggestion, we have now also emphasized it in the paragraph discussing the by-chance intersection of the mapped reads with the predicted microbial-like regions.

Reviewer #2:

Summary
=================

This manuscript addresses a significant issue in ancient metagenomics: how microbial contamination in reference genomes can influence taxonomic profiling and analytical outcomes. It is a well-written, timely, and highly relevant study. The structure is logical, the arguments are generally sound, and the results are important for the field. However, there are several major limitations that must be addressed to strengthen the study.

I believe this work could be of interest to the readers of GigaScience, but the current analysis and documentation require substantial improvement.

Authors' comment: we thank the reviewer for the very valuable comments, they have helped us to substantially improve the workflow and the manuscript. Below, we address in bold the reviewer's comments point-by-point.

Major Comments
=================

1. Handling of conserved and closely related genes/genomes

It is unclear how the workflow accounts for photosynthetic or ancestrally derived genomic regions in plants, which may be legitimate components of plant genomes. Classifying such regions as microbial contamination may not be appropriate, and masking them in downstream analyses could distort results. For example, GTDB includes over 5,000 cyanobacterial genomes, some of which are among the closest known relatives to plants. Given the potential for genuine sequence similarity, particularly under permissive

mapping parameters, the reported contamination may be overestimated. This concern is also relevant to conclusions drawn from single-sample tests.

Authors' response: thank you, we agree this is an important question to discuss further. The GTDB version r214, that was used in this study, contains 3,846 reference genomes (out of the total 394,932 genomes) belonging to the Cyanobacteriota phylum, representing only ~1% of the total number of reference sequences in GTDB. We now tested the enrichment of cyanobacterial pseudo-reads in the predicted microbial-like regions of Hippuris vulgaris and Claytonia eschscholtzii plants from the PhyloNorway dataset, which were predicted to contain the highest percentage of microbial-like regions, 57% and 70%, respectively. Based on the annotation from the GTDB r214, there were 622,336,764 cyanobacterial pseudo-reads out of total 26,089,195,106 pseudo-reads, i.e. 2.4%, pre-computed from the GTDB reference sequences. However, we observed only 2,739,015 cyanobacterial pseudo-reads of of total 481,618,468 (i.e. 0.6%) aligned to the Hippuris vulgaris, and only 1,688,444 cyanobacterial pseudo-reads out of 408,824,087 (i.e. 0.4%) aligned to Claytonia eschscholtzii, reference genomes from the PhyloNorway dataset. This implies that cyanobacterial pseudo-reads do not align more often to the two PhyloNorway plant references than would be expected by chance. Therefore, although potential over-masking of plant references following our method is plausible, it is not supported by our analysis of GTDB data. Nevertheless, because of ambiguity of the analysis, we cannot fully reject the hypothesis that due to the genuine sequence similarity between cyanobacterial and plant genomes, the fractions of microbial-like regions in some plant reference genomes predicted in this study may be overestimated. However, if such over-masking occurs and these regions represent genuine host genome sequences, microbial sequences in aeDNA samples can still align to them, potentially leading to erroneous taxonomic assignments. In this context, masking these regions remains beneficial, as it promotes a more conservative approach and thus a more reliable detection of true species present in ancient samples.

We have now added these results to the Discussion section of the revised manuscript.

2. Contamination in GTDB Database

The manuscript does not address possible contamination within the GTDB database itself, which appears to be used uncritically for cleaning eukaryotic reference genomes. Prior work has raised concerns about microbial genome contamination (e.g., https://pubmed.ncbi.nlm.nih.gov/38809778/). At a minimum, this limitation should be discussed; ideally, it should be assessed computationally. For instance, pseudo-reads generated from a well-annotated genome (e.g., human) could be mapped against microbial references to demonstrate the complexity and potential pitfalls in cross-superkingdom contamination detection.

Alternatively, I encourage the authors to develop or include a method to evaluate the specificity and sensitivity of the contaminated regions identified in the eukaryotic genomes. This would reduce reliance on external assumptions and provide stronger validation for their conclusions.

Authors' response: thank you, we agree this is an important consideration. We indeed assume GTDB to represent a microbial "ground-truth" and free from eukaryotic contamination as it is a highly curated database. We aimed to further validate this assumption by the additional analysis that we performed by aligning human pseudo-reads (prepared in the same way as the microbial pseudo-reads described in the manuscript) to >820 random GTDB and 25 RefSeq microbial reference sequences. In the whole experiment we observed only 3 human reads aligned to any microbial reference which represents a negligible breadth of coverage with median 0% and ensures that the microbial pseudo-reads used in this manuscript are very likely free from any substantial eukaryotic contamination.

In contrast, when we screen eukaryotic reference genomes for the presence of human-like sequences, we discover substantially higher coverage. For example, Spirometra erinaceieuropaei (parasitic tapeworm) reference genome GCA_000951995.1, indicated over 8 million aligned human pseudo-reads covering >0.1% of the reference genome with the total length of human-like sequences of 1.4 Mbp. We present an IGV visualization of one of 100% covered scaffolds of the parasitic tapeworm reference in the newly included Supplementary Figure 15. In addition, screening the reference genome GCF_002220235.1 of

Bathycoccus prasinos (green algae), we detected over 236,000 aligned human pseudo-reads covering ~0.2% of the reference with the total length of the potentially exogenous regions of 37 kbp.

To further address the sensitivity and specificity of our method, we ran it using a random subset of 6.5 million GTDB pseudo-reads and the screened reference genome represented concatenated hg38 human reference genome with 16 microbial reference genomes (corresponding to 726 reference sequences on chromosome and scaffold level) used in Pochon at al. Genome Biology 2023. We observed only 1 microbial pseudo-read mapped to one chromosome (chr 12) out of 24 canonical and 432 decoy chromosomes in the human hg38 reference genome, while 202,762 pseudo-reads were mapped to the microbial reference sequences. This demonstrates the high specificity of our method as well as the very low risk of Bowtie2 non-specific alignments (please see the next reviewer's comment regarding the risk of including non-specific alignments).

We have now added this analysis to the Methods, Discussion as well as Supplementary Material S5 sections of the revised manuscript.

3. Quantitative Analysis of Sequence Similarity

The manuscript lacks a detailed analysis of the similarity between microbial reads and the eukaryotic regions they map to. Given that the Bowtie2 setting --very-sensitive allows alignments with substantial mismatches (e.g., 60 bp reads with >10 bp differences), there is a risk of including non-specific alignments. A quantitative analysis of sequence identity across these alignments would be helpful, along with a justified similarity threshold to exclude highly divergent mappings that may not be of microbial origin.

Authors' response: thank you for this very valid point. Bowtie2 has a special non-trivial scoring system to determine whether a read will be mapped or not. The scoring system is not solely based on the exact number of mismatches but includes multiple other metrics such as base quality, gaps, clipping etc. Nevertheless, Bowtie2 prioritizes high-scoring (i.e. more similar) alignments, and heavily penalizes divergence. Empirically, we can see that a 60 bp read with more than ~6 mismatches (average nucleotide identity ANI=90%) will usually fail to align, even under --very-sensitive –end-to-end. For example, tested on RefSeq plants and PhyloNorway references with aligned GTDB pseudo-reads, we observe on average 4 ± 0.4 and 4 ± 0.2 mismatches per read, respectively, i.e. the similarity of ANI=93%. The comment that "Bowtie2 setting --very-sensitive allows alignments with substantial mismatches (e.g., 60 bp reads with >10 bp differences)" is not directly supported by our empirical testing. Taking into account that a typical ANI threshold for ancient metagenomics projects is set much lower than 93%, i.e. often down to 85% (Pochon et al. 2023, Huebler et al. 2019), due to DNA damage, we assume the risk of non-specific alignments with the Bowtie2 mapping parameters used in this study is low.

Also (please see the answer to the previous question), in the experiment when we aligned human pseudo-reads to microbial references and vice-versa microbial pseudo-reads to concatenated human + microbial references we discovered extremely few (3 and 1 respectively) miss-mapped reads, which highlights the high specificity of Bowtie2 alignments with –very-sensitive and –end-to-end arguments used in this study.

We have now added this additional analysis of sequence similarity threshold to the Supplementary Material S1 section.

4. Reproducibility and Workflow Usability

The computational workflow should be included in the GitHub repository to ensure reproducibility. I attempted to run the small test example provided, but encountered multiple issues on both a Unix-based laptop and a Linux server. These included absolute paths in scripts, unresolved dependencies, and failures in execution. I recommend that the authors:

- Fix hardcoded paths and ensure portability across environments
- Provide a working bash script that runs successfully on clean systems
- Include a conda environment file or, at a minimum, document tool versions and dependencies

Here is a sample of the errors encountered:

```
xxxxxx@xxxxxxxxx MCWorkflow % ./micr_cont_detect.sh GCF_002220235.fna.gz data GTDB 4
GTDB_sliced_seqs_sliding_window.fna.gz GTDB_fna2name.txt

PREPARING FILES FOR ANALYSIS OF GCF_002220235.fna.gz REFERENCE GENOME
mkdir: GCF_002220235.fna.gz_GTDB: File exists

BUILDING BOWTIE2 INDEX FOR GCF_002220235.fna.gz REFERENCE GENOME
ALIGNING MICROBIAL READS WITH BOWTIE2 TO GCF_002220235.fna.gz REFERENCE GENOME
(ERR): "data/GCF_002220235.fna.gz" does not exist or is not a Bowtie 2 index
Exiting now ...
[main_samview] fail to read the header from "-".
[W::hts_set_opt] Cannot change block size for this format
samtools sort: failed to read header from "-"
samtools index: "MicrReads_aligned_to_GCF_002220235.fna.gz.bam" is in a format that cannot be usefully
indexed
RANKING GCF_002220235.fna.gz CONTIGS BY NUMBER OF MAPPED MICROBIAL READS
[main_samview] fail to read the header from "MicrReads_aligned_to_GCF_002220235.fna.gz.bam".
sed: 1: "contigs_abund_sorted_GT ...": command c expects \ followed by text
sed: 1: "contigs_abund_sorted_GT ...": command c expects \ followed by text
COMPUTING BREADTH OF COVERAGE FOR EACH CONTIG AND COORDINATES OF MICROBIAL
CONTAMINATION FOR GCF_002220235.fna.gz REFERENCE GENOME

AGGREGATING RESULTS FOR GCF_002220235.fna.gz REFERENCE GENOME AND CLEANING
paste: total_length_per_ref.txt: No such file or directory
sed: 1: "contigs_boc_sorted_GTDB ...": command c expects \ followed by text
sed: 1: "contigs_boc_sorted_GTDB ...": command c expects \ followed by text
rm: total_length_per_ref.txt: No such file or directory
rm: boc_per_ref.txt: No such file or directory
COMPUTING LIST OF MOST ABUNDANT MICROBES CONTMAMINATING GCF_002220235.fna.gz REFERENCE
GENOME
[main_samview] fail to read the header from "MicrReads_aligned_to_GCF_002220235.fna.gz.bam".

ANALYSIS FOR GCF_002220235.fna.gz REFERENCE GENOME FINISHED SUCCESSFULLY
xxxxxx@xxxxxxxxx MCWorkflow % ll data
total 103232
-rwxr-xr-x 1 xxxxxx staff 4941250 May 9 11:35 GCF_002220235.fna.gz
-rw-r--r-- 1 xxxxxx staff 13403961 May 9 11:50 GCF_002220235.fna.gz.1.bt2l
-rw-r--r-- 1 xxxxxx staff 7519068 May 9 11:50 GCF_002220235.fna.gz.2.bt2l
-rw-r--r-- 1 xxxxxx staff 709 May 9 11:50 GCF_002220235.fna.gz.3.bt2l
-rw-r--r-- 1 xxxxxx staff 3759531 May 9 11:50 GCF_002220235.fna.gz.4.bt2l
-rw-r--r-- 1 xxxxxx staff 13403961 May 9 11:50 GCF_002220235.fna.gz.rev.1.bt2l
-rw-r--r-- 1 xxxxxx staff 7519068 May 9 11:50 GCF_002220235.fna.gz.rev.2.bt2l
drwxr-xr-x 6 xxxxxx staff 192 May 9 11:50 GCF_002220235.fna.gz_GTDB
-rw-r--r-- 1 xxxxxx staff 67376 May 9 11:50 bowtie2-build.log
```

Authors’ response: thank you very much for testing our workflow and your suggestions! We agree and we have now substantially improved the reproducibility and usability of the workflow. Specifically, first, we

resolved the hard-coded paths issue which now makes it possible to run the workflow from the cloned repository without manually editing the paths. Second, we added the conda environment file, that assists installing the workflow tools, and corresponding clarifications to the README in the github repository page. Finally, we wrapped the workflow up via the Nextflow framework for further scalability and reproducibility. The workflow has been tested on one laptop, one stationary workstation and two computer clusters where it ran without errors. We invite the reviewer to test the workflow again following the command lines presented here:

https://github.com/NikolayOskolkov/MCWorkflow?tab=readme-ov-file#quick-start.

Editorial Manager®

Cookie Preference Center

We use cookies which are necessary to make our site work. We may also use additional cookies to analyze, improve and personalize our content and your digital experience. For more information, see our Cookie Policy.

You may choose not to allow some types of cookies. However, blocking some types may impact your experience of our site and the services we are able to offer. See the different category headings below to find out more or change your settings.

Allow all

Manage Consent Preferences

Strictly Necessary Cookies

Always active

These cookies are necessary for the website to function and cannot be switched off in our systems. They are usually only set in response to actions made by you which amount to a request for services, such as setting your privacy preferences, logging in or filling in forms. You can set your browser to block or alert you about these cookies, but some parts of the site will not then work.

Functional Cookies

These cookies enable the website to provide enhanced functionality and personalisation. They may be set by us or by third party providers whose services we have added to our pages. If you do not allow these cookies then some or all of these services may not function properly.

Performance Cookies

These cookies allow us to count visits and traffic sources so we can measure and improve the performance of our site. They help us to know which pages are the most and least popular and see how visitors move around the site.

Targeting Cookies

These cookies may be set through our site by our advertising partners. They may be used by those companies to build a profile of your interests and show you relevant adverts on other sites. If you do not allow these cookies, you will experience less targeted advertising.

Cookie List

Clear

Apply Cancel
Consent Leg.Interest

Confirm my choices

Powered by onetrus
