# Supplementary material for: Improving taxonomic inference from ancient environmental metagenomes by masking microbial-like regions in reference genomes
Source: Gigascience. 2025 Oct 3;14:giaf108. doi: 10.1093/gigascience/giaf108 (PMC12491943; doi:10.1093/gigascience/giaf108)
Supplement: giaf108_Reviewer_1_Report_Original_Submission — Lucas Czech -- 4/15/2025 [file giaf108_reviewer_1_report_original_submission.pdf]

Reviewer Report

Title: Disinfecting eukaryotic reference genomes to improve taxonomic inference from ancient environmental metagenomic data

Version: Original Submission **Date:** 4/15/2025

Reviewer name: Lucas Czech

Reviewer Comments to Author:

Summary=================Oskolkov et al present an approach to identify microbial contamination in reference sequence databases. They use this approach to scrutinize two previous publications in the ancient environmental DNA field, and show that some species found in these papers are likely over-estimations due to contamination in the references. They further investigate which microbial references are mostly responsible for the contamination in the examined databases, and show that the PhyloNorway references are highly contaminated.The problem that Oskolkov et al are tackling here is highly relevant for practitioners, not just in the aeDNA field, but in whole-genome eDNA studies in general. Their findings are worrying to say the least, and indicate that future research and development is needed to fully solve the problem of contamination. This manuscript is also a cautionary tale for data sanitation, and should be read by anyone working with aeDNA.The methodological approach is straight-forward and sound, and to my understanding makes more sense than the two existing methods that are referred to in the manuscript. Mapping the contaminants against the target (Eukaryotes) seems intuitively more reasonable than the other way round, and allows the authors to produce masks for the genomes, which is interesting and relevant in practice in order to obtain masks.The paper is very well written and easy to follow. Furthermore, all analyses are thorough, and the figures support the claims that the authors make. All scripts and datasets are made available, and seem well documented.In summary, the paper is of high quality, and definitely should be published. I do have some suggestions on improvements, which I list below.Best regardsLucas CzechMajor=================* One suggestion that we discussed internally after reading the preprint of the manuscript is the following: Using the presented method to map the pseudo-reads against GTDB itself, and/or against the RefSeq organelle or microbial sequences. This would serve as a further validation (everything should show up as "contaminated"), and can be used as a baseline for accuracy and other evaluations.* Furthermore, would it make sense to include the human genome as a source of contamination, i.e., create pseudo-reads from it as well? The authors mention that references might be contaminated with human reads, and the workflow as presented should easily be able to accommodate for that, in order to catch that contamination.* The available scripts are great, however might make it a bit hard to use the approach in practice on other datasets or later versions. To fully leverage the method, and for practitioners to be able to use it on their own data, I suggest to develop this into a proper workflow (e.g., Snakemake), taking a set of reference fasta files and a set of contaminant fasta files as input, and running through all steps automatically. Given that the scripts already exist, this might be relatively easy to implement, and of great use for the community. I understand though if the authors feel that this is out of scope for this manuscript.Minor=================* Line 62: "the presence of hippopotamus-like sequence" is

syntactically incorrect. Either "the presence of a hippopotamus-like sequence", or "sequences" in plural would work.* Line 137, and Supplement S1: Using 60 bp pseudo-reads is yielding very specific hits, which might be what is needed here. Have the authors evaluated smaller pseudo-reads as well? As 30-ish is usually enough to identify a species, I am curious to hear the author's view on this - why 60, and not 30, 40, or 50? Wouldn't those potentially catch more contamination? Or would that be too sensitive and give too many false positives?* Line 141, and Supp S1: Allowing for multi-mapping reads is certainly necessary for the reasons the authors have laid out. I am wondering however if 10 is enough here. Have the authors checked if that number is saturated for some genomes? If so, it might need increasing. Addendum after getting to the supplement: This was indeed evaluated, great! But only on two genomes - might be good enough, but it might differ for other species, or not? I am lacking intuition here, and would like to hear the author's take on this.* Lines 168-172: While this is the methods section, a very short description of why this PCA was computed as a means of validation, or a foreshadowing link to Fig 4, might help the reader here to understand the reason behind this here already.* Lines 220ff: I needed several attempts to read and understand this sentence, and find it confusingly phrased. Might I suggest something along the lines of:"There are 81 primate genomes in the 566 assessed mammalian genomes. Yet, 37 of these are within the top 45 most contaminated genomes."Also, it might be interesting to report the range of contamination of these 37 genomes.* Lines 295ff: The authors very thoroughly compute the expected percentage of overlap with regions identified as microbial-like in the Hippuris vulgaris reference. How much of that genome was reported as being microbial-like though? Maybe I've missed this, but that might be an interesting fraction to report here as well.

Level of Interest

Please indicate how interesting you found the manuscript: Choose an item.

Quality of Written English

Please indicate the quality of language in the manuscript: Choose an item.

Declaration of Competing Interests

Please complete a declaration of competing interests, considering the following questions:

- Have you in the past five years received reimbursements, fees, funding, or salary from an organisation that may in any way gain or lose financially from the publication of this manuscript, either now or in the future?
- Do you hold any stocks or shares in an organisation that may in any way gain or lose financially from the publication of this manuscript, either now or in the future?
- Do you hold or are you currently applying for any patents relating to the content of the manuscript?
- Have you received reimbursements, fees, funding, or salary from an organization that holds or has applied for patents relating to the content of the manuscript?

- Do you have any other financial competing interests?
- Do you have any non-financial competing interests in relation to this paper?

If you can answer no to all of the above, write 'I declare that I have no competing interests' below. If your reply is yes to any, please give details below.

I declare that I have no competing interests

I agree to the open peer review policy of the journal. I understand that my name will be included on my report to the authors and, if the manuscript is accepted for publication, my named report including any attachments I upload will be posted on the website along with the authors' responses. I agree for my report to be made available under an Open Access Creative Commons CC-BY license (http://creativecommons.org/licenses/by/4.0/). I understand that any comments which I do not wish to be included in my named report can be included as confidential comments to the editors, which will not be published.

Choose an item.

To further support our reviewers, we have joined with Publons, where you can gain additional credit to further highlight your hard work (see: https://publons.com/journal/530/gigascience). On publication of this paper, your review will be automatically added to Publons, you can then choose whether or not to claim your Publons credit. I understand this statement.

Yes Choose an item.
