# Supplementary material for: Improving taxonomic inference from ancient environmental metagenomes by masking microbial-like regions in reference genomes
Source: Gigascience. 2025 Oct 3;14:giaf108. doi: 10.1093/gigascience/giaf108 (PMC12491943; doi:10.1093/gigascience/giaf108)
Supplement: giaf108_Reviewer_2_Report_Original_Submission — Mikkel Winther Pedersen -- 5/9/2025 [file giaf108_reviewer_2_report_original_submission.pdf]

Reviewer Report

Title: Disinfecting eukaryotic reference genomes to improve taxonomic inference from ancient environmental metagenomic data

Version: Original Submission **Date:** 5/9/2025

Reviewer name: Mikkel Winther Pedersen

Reviewer Comments to Author:

review GigaScienceThis manuscript addresses a significant issue in ancient metagenomics: how microbial contamination in reference genomes can influence taxonomic profiling and analytical outcomes. It is a well-written, timely, and highly relevant study. The structure is logical, the arguments are generally sound, and the results are important for the field. However, there are several major limitations that must be addressed to strengthen the study.I believe this work could be of interest to the readers of GigaScience, but the current analysis and documentation require substantial improvement.Major Comments1. Handling of conserved and closely related genes/genomesIt is unclear how the workflow accounts for photosynthetic or ancestrally derived genomic regions in plants, which may be legitimate components of plant genomes. Classifying such regions as microbial contamination may not be appropriate, and masking them in downstream analyses could distort results. For example, GTDB includes over 5,000 cyanobacterial genomes, some of which are among the closest known relatives to plants. Given the potential for genuine sequence similarity, particularly under permissive mapping parameters, the reported contamination may be overestimated. This concern is also relevant to conclusions drawn from single-sample tests.2. Contamination in GTDB DatabaseThe manuscript does not address possible contamination within the GTDB database itself, which appears to be used uncritically for cleaning eukaryotic reference genomes. Prior work has raised concerns about microbial genome contamination (e.g., https://pubmed.ncbi.nlm.nih.gov/38809778/). At a minimum, this limitation should be discussed; ideally, it should be assessed computationally. For instance, pseudo-reads generated from a well-annotated genome (e.g., human) could be mapped against microbial references to demonstrate the complexity and potential pitfalls in cross-superkingdom contamination detection.Alternatively, I encourage the authors to develop or include a method to evaluate the specificity and sensitivity of the contaminated regions identified in the eukaryotic genomes. This would reduce reliance on external assumptions and provide stronger validation for their conclusions.3. Quantitative Analysis of Sequence SimilarityThe manuscript lacks a detailed analysis of the similarity between microbial reads and the eukaryotic regions they map to. Given that the Bowtie2 setting --very-sensitive allows alignments with substantial mismatches (e.g., 60 bp reads with >10 bp differences), there is a risk of including non-specific alignments. A quantitative analysis of sequence identity across these alignments would be helpful, along with a justified similarity threshold to exclude highly divergent mappings that may not be of microbial origin.4. Reproducibility and Workflow UsabilityThe computational workflow should be included in the GitHub repository to ensure reproducibility. I attempted to run the small test example provided, but encountered multiple issues on both a Unix-based laptop and a Linux server. These included absolute paths in scripts, unresolved dependencies, and

failures in execution. I recommend that the authors:- Fix hardcoded paths and ensure portability across environments- Provide a working bash script that runs successfully on clean systems- Include a conda environment file or, at a minimum, document tool versions and dependenciesHere is a sample of the errors encountered:```xxxxxx@xxxxxxxxx MCWorkflow % ./micr_cont_detect.sh GCF_002220235.fna.gz data GTDB 4 GTDB_sliced_seqs_sliding_window.fna.gz GTDB_fna2name.txtPREPARING FILES FOR ANALYSIS OF GCF_002220235.fna.gz REFERENCE GENOMEmkdir: GCF_002220235.fna.gz_GTDB: File existsBUILDING BOWTIE2 INDEX FOR GCF_002220235.fna.gz REFERENCE GENOMEALIGNING MICROBIAL READS WITH BOWTIE2 TO GCF_002220235.fna.gz REFERENCE GENOME(ERR): "data/GCF_002220235.fna.gz" does not exist or is not a Bowtie 2 indexExiting now ...[main_samview] fail to read the header from "-".[W::hts_set_opt] Cannot change block size for this formatsamtools sort: failed to read header from "-"samtools index: "MicrReads_aligned_to_GCF_002220235.fna.gz.bam" is in a format that cannot be usefully indexedRANKING GCF_002220235.fna.gz CONTIGS BY NUMBER OF MAPPED MICROBIAL READS[main_samview] fail to read the header from "MicrReads_aligned_to_GCF_002220235.fna.gz.bam".sed: 1: "contigs_abund_sorted_GT ...": command c expects \ followed by textsed: 1: "contigs_abund_sorted_GT ...": command c expects \ followed by textCOMPUTING BREADTH OF COVERAGE FOR EACH CONTIG AND COORDINATES OF MICROBIAL CONTAMINATION FOR GCF_002220235.fna.gz REFERENCE GENOMEAGGREGATING RESULTS FOR GCF_002220235.fna.gz REFERENCE GENOME AND CLEANINGpaste: total_length_per_ref.txt: No such file or directorysed: 1: "contigs_boc_sorted_GTDB ...": command c expects \ followed by textsed: 1: "contigs_boc_sorted_GTDB ...": command c expects \ followed by textrm: total_length_per_ref.txt: No such file or directoryrm: boc_per_ref.txt: No such file or directoryCOMPUTING LIST OF MOST ABUNDANT MICROBES CONTMAMINATING GCF_002220235.fna.gz REFERENCE GENOME[main_samview] fail to read the header from "MicrReads_aligned_to_GCF_002220235.fna.gz.bam".ANALYSIS FOR GCF_002220235.fna.gz REFERENCE GENOME FINISHED SUCCESSFULLYxxxxxx@xxxxxxxxx MCWorkflow % ll datatotal 103232-rwxr-xr-x 1 xxxxxx staff 4941250 May 9 11:35 GCF_002220235.fna.gz-rw-r--r-- 1 xxxxxx staff 13403961 May 9 11:50 GCF_002220235.fna.gz.1.bt2l-rw-r--r-- 1 xxxxxx staff 7519068 May 9 11:50 GCF_002220235.fna.gz.2.bt2l-rw-r--r-- 1 xxxxxx staff 709 May 9 11:50 GCF_002220235.fna.gz.3.bt2l-rw-r--r-- 1 xxxxxx staff 3759531 May 9 11:50 GCF_002220235.fna.gz.4.bt2l-rw-r--r-- 1 xxxxxx staff 13403961 May 9 11:50 GCF_002220235.fna.gz.rev.1.bt2l-rw-r--r-- 1 xxxxxx staff 7519068 May 9 11:50 GCF_002220235.fna.gz.rev.2.bt2ldrwxr-xr-x 6 xxxxxx staff 192 May 9 11:50 GCF_002220235.fna.gz_GTDB-rw-r--r-- 1 xxxxxx staff 67376 May 9 11:50 bowtie2-build.log```

Level of Interest

Please indicate how interesting you found the manuscript: Choose an item.

Quality of Written English

Please indicate the quality of language in the manuscript: Choose an item.

Declaration of Competing Interests

Please complete a declaration of competing interests, considering the following questions:

- Have you in the past five years received reimbursements, fees, funding, or salary from an organisation that may in any way gain or lose financially from the publication of this manuscript, either now or in the future?
- Do you hold any stocks or shares in an organisation that may in any way gain or lose financially from the publication of this manuscript, either now or in the future?
- Do you hold or are you currently applying for any patents relating to the content of the manuscript?
- Have you received reimbursements, fees, funding, or salary from an organization that holds or has applied for patents relating to the content of the manuscript?
- Do you have any other financial competing interests?
- Do you have any non-financial competing interests in relation to this paper?

If you can answer no to all of the above, write 'I declare that I have no competing interests' below. If your reply is yes to any, please give details below.

I have no competing interests.

I agree to the open peer review policy of the journal. I understand that my name will be included on my report to the authors and, if the manuscript is accepted for publication, my named report including any attachments I upload will be posted on the website along with the authors' responses. I agree for my report to be made available under an Open Access Creative Commons CC-BY license (http://creativecommons.org/licenses/by/4.0/). I understand that any comments which I do not wish to be included in my named report can be included as confidential comments to the editors, which will not be published.

Choose an item.

To further support our reviewers, we have joined with Publons, where you can gain additional credit to further highlight your hard work (see: https://publons.com/journal/530/gigascience). On publication of this paper, your review will be automatically added to Publons, you can then choose whether or not to claim your Publons credit. I understand this statement.

Yes Choose an item.
